# Scrutinizing Coronaviruses Using Publicly Available Bioinformatic Tools: The Viral Structural Proteins as a Case Study

**DOI:** 10.3389/fmolb.2021.671923

**Published:** 2021-05-24

**Authors:** Sonia Beeckmans, Edilbert Van Driessche

**Affiliations:** Research Unit Protein Chemistry, Vrije Universiteit Brussel, Brussels, Belgium

**Keywords:** beta-coronavirus, COVID-19, SARS-CoV-2, SARS-CoV, MERS-CoV, spike protein, pandemic, DeepView molecular graphics program

## Abstract

Since early 2020, the world suffers from a new beta-coronavirus, called SARS-CoV-2, that has devastating effects globally due to its associated disease, Covid-19. Until today, Covid-19, which not only causes life-threatening lung infections but also impairs various other organs and tissues, has killed hundreds of thousands of people and caused irreparable damage to many others. Since the very onset of the pandemic, huge efforts were made worldwide to fully understand this virus and numerous studies were, and still are, published. Many of these deal with structural analyses of the viral spike glycoprotein and with vaccine development, antibodies and antiviral molecules or immunomodulators that are assumed to become essential tools in the struggle against the virus. This paper summarizes knowledge on the properties of the four structural proteins (spike protein S, membrane protein M, envelope protein E and nucleocapsid protein N) of the SARS-CoV-2 virus and its relatives, SARS-CoV and MERS-CoV, that emerged few years earlier. Moreover, attention is paid to ways to analyze such proteins using freely available bioinformatic tools and, more importantly, to bring these proteins alive by looking at them on a computer/laptop screen with the easy-to-use but highly performant and interactive molecular graphics program DeepView. It is hoped that this paper will stimulate non-bioinformaticians and non-specialists in structural biology to scrutinize these and other macromolecules and as such will contribute to establishing procedures to fight these and maybe other forthcoming viruses.

## Introduction

The year 2020 will always be remembered as “*the year of the pandemic.*” A new type of virus causing severe respiratory illness emerged in December 2019 in Wuhan, China. Since then, it has rapidly spread throughout the entire world, leaving a trail of destruction with high mortality. The pathogen was soon identified to belong to the *Coronaviridae* family, subfamily of the *Coronavirinae*, which is further subdivided in four genera called alpha, beta, gamma and delta (Belouzard et al., 2012; Fehr and Perlman, 2015; Fung and Liu, 2019; Li et al., 2020a). The new virus could be classified as a beta-coronavirus and was found to be closely related to other human beta-coronaviruses that emerged early in the 21-st century, i.e., SARS-CoV that died out after about one year and MERS-CoV that is still lingering. The new virus was officially named SARS-CoV-2 and the disease it causes is known as Covid-19 (Gorbalenya et al., 2020). The original SARS-CoV-2 virus, which evolved in bats (Andersen et al., 2020; Shereen et al., 2020) like many other alpha- and beta-coronaviruses (Li et al., 2005; Chan et al., 2013; Wang and Anderson, 2019), is easily transmitted from human to human, has an appreciably high reproductive number when no containment measures are taken (*R*_*o*_ = 2–4), a high infection fatality rate (IFR = 0.3–1.3%), and it remains infective for extensive periods of time outside the human body (Bar-On et al., 2020; Rabi et al., 2020). Moreover, it spreads already for several days before an infected person notices the first symptoms of disease because the virus developed several ways to thwart the immune system’s response (Astuti and Ysrafil, 2020; Banerjee et al., 2020; Kikkert, 2020). SARS-CoV-2, together with SARS-CoV and MERS-CoV (hereafter referred to as the SARS-CoV-s), are still of great concern because of their worldwide health threat to humans. For this reason, since its first appearance, numerous studies have been conducted to understand its structure, organization, ways of infection, multiplication and pathogenesis. These studies are anticipated to continue guiding us in the development of strategies using antivirals and/or immunomodulators to attenuate the severity of illness in case of infection, and/or to prevent infection through the development of vaccines (Abd Ellah et al., 2020; Capell et al., 2020; Callaway, 2020a; Dai et al., 2020; Dong et al., 2020; Graham, 2020; Hu et al., 2020a; Kaslow, 2020; Krammer, 2020; Li et al., 2020c; Liu et al., 2020; Poland et al., 2020; Riva et al., 2020; Tay et al., 2020), thereby trying to avoid serious problems that may show up such as cytokine storm development, suboptimal antibody response or immune enhancement (Tisoncik et al., 2012; de Alwis et al., 2020; Hotez et al., 2020; Iwasaki and Yang, 2020; Moore and June, 2020; Pedersen and Ho, 2020). Another point of attention should be the prevention of mutational escape of viral proteins that seems to occur following administration of single antibody species (Baum et al., 2020). Such studies are all the more important as it is envisaged that many more SARS-CoV/MERS-CoV-like coronaviruses might be lurking around the corner, ready to jump to and thereafter spread amongst humans following interspecies transmission in the years or decennia to come (Wang and Anderson, 2019; Valitutto et al., 2020).

## Aim of the Paper

This paper presents an overview of the current knowledge of the SARS-CoV-s’ structural proteins, on their spatial organization and functional properties, with emphasis on the spike protein. Also, the involvement of the host’s own proteins in the development of Covid-19 is considered. Moreover, attention is paid to how antibodies and peptides may help to overcome infections. In the Supplementary Material to this paper, we will explore and demonstrate how bioinformatic tools that are freely available on the internet may help students and researchers who are neither trained bioinformaticians nor structural biologists, to understand and visualize macromolecules such as those from the beta-coronaviruses. In view of the overwhelming numbers of structural studies and the continuous increasing availability of data in the protein databank (wwPDB consortium, 2019; Berman et al., 2000), it is definitely an asset to be able to visualize (macro)molecules on a personal computer screen. Although authors do their utmost to present structures they show in publications in optimal orientations and with the most instructive coloring, it is essential to be able to walk around in these structures yourself to gain a much better understanding of these molecules and appreciate their 3D structure and flexibility. Therefore, the Supplementary Material will guide the reader within this exciting area, which steadily continues to grow in importance. Playing around with the structural data that are amply available nowadays is becoming a prerequisite to understand complex particles such as the SARS-CoV-s and helps us to deal with them. In fact, looking in detail to the structures of the respective viral components allows us to understand the whole sequence of events that occur during infection and pathogenesis of the virus.

## The Genome of the SARS-CoV-s

Coronaviruses are (+)ssRNA (positive-sense single-stranded RNA) viruses with a very large RNA genome, typically around 30 kb. The viral RNA is packaged inside a spherical membrane (roughly 100–125 nm in diameter) with the help of soluble nucleocapsid proteins (N). Other structural proteins comprise three membrane proteins, i.e., the spike protein (S), the membrane protein (M) (occasionally also called matrix protein) and the envelope protein (E). These four structural proteins occur at a ratio of roughly (E:S:N:M) = (1:5:50:100), according to estimations done for SARS-CoV (Bar-On et al., 2020).

**FIGURE 1:**
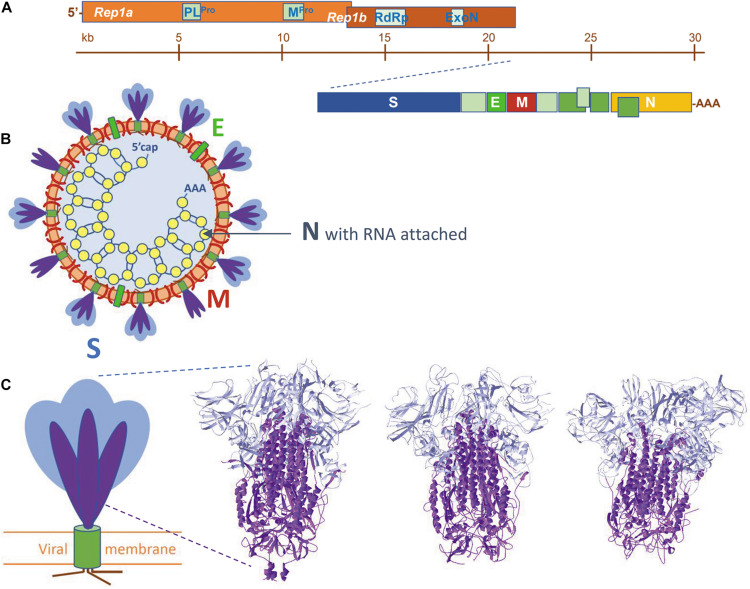
**(A)** The organization of the viral RNA, coding for, amongst others, the four structural proteins and additional non-structural proteins, e.g., enzymes mentioned in the text. **(B)** Schematic representation of a SARS-coronavirion with its four structural proteins S, M, E, and N. The spike proteins S are present as a mixture of intact proteins (S1 plus S2), but some having already lost their S1 portion. **(C)** To the left: schematic view of a SARS-CoV spike protein. S proteins are homotrimers, each subunit is built of two portions, S1 (blue) and S2 (purple), followed by a transmembrane helix (olive) and a small cytoplasmic tail (brown). To the right: the spike protein trimer ectodomains of (from left to right) SARS-CoV-2 (from 6VXX.pdb), SARS-CoV (from 5XLR.pdb) and MERS-CoV (from 5X5C.pdb). Peptide segments S1 and S2 are colored as in the schematic view. Literature references for structural codes: 6VXX.pdb (Walls et al., 2020); 5XLR.pdb (Gui et al., 2017); 5X5C.pdb (Yuan et al., 2017).

At the 5′ end, the viral RNA contains two large so-called replicase genes (*rep1a*, *rep1b*) organized as two extended open reading frames (5′-ORF-1a/1b), followed by genes that code for the four structural and some accessory proteins (Fehr and Perlman, 2015; Tang et al., 2020; Figure 1). As soon as the viral RNA enters a host cell, it acts as an mRNA molecule and hijacks not only the host translational machinery to make all its encoded proteins, but also the host’s intricate post-translational modification systems. The open reading frames, 5′-ORF-1a/1b, encode a series of non-structural proteins (Nsps), some of which are enzymes, while others have yet unknown functions (Fehr and Perlman, 2015; Chen et al., 2020). Translation of these two ORFs in the cytoplasm of the host cell results in the synthesis of two long polyproteins (pp1a, pp1b), which are autoproteolytically cleaved by viral proteases. One of the enzymes (Nsp12) is a unique RNA-dependent RNA polymerase (RdRp), responsible for replication of the viral RNA genome (Jiang et al., 2020). Another one (Nsp14) also has an essential role in replication and transcription: it is a bifunctional enzyme with an exoribonuclease domain (ExoN) that, extraordinary for a virus, has proofreading activity and, as such, limits the occurrence of lethal mutations in the viral RNA (Ferron et al., 2017; Romano et al., 2020). Moreover, two important viral proteases are produced from its RNA, i.e., the main protease (M^*Pro*^, also called 3CL^*Pro*^) and a papain-like protease (PL^*Pro*^), both implicated in processing of the polyproteins (Zhang et al., 2020a). Besides other components, numerous copies of the nucleocapsid protein N are also made in the cytoplasm.

## From RNA to Mature Virions

Upon infection, (+)ssRNA viruses usurp host cell membranes from certain organelles. In SARS-CoV-s, ER membranes are captured from which a complex reticulovesicular network forms that contains double membrane vesicles (DMVs, 200–300 nm) and which remains connected to the ER (Snijder et al., 2020). DMVs act as little factories in which ssRNA is first transformed into dsRNA (an intermediate in multiplication), from which new viral (+)ssRNA molecules are generated. In this way, viral RNA is isolated and shielded from innate immune sensing. This entire process also relies on several viral Nsps that form complexes, many of which have yet to be unraveled. Some Nsps assemble to form pores in the DMV membranes, through which newly made ssRNA molecules are exported into the host cytoplasm (Wolff et al., 2020). During this process they collect N proteins that bind in a “beads-on-a-string” fashion to stabilize the RNA (Yao et al., 2020). These RNP complexes then travel to virus assembly sites at the ERGIC and/or Golgi complex.

Meanwhile, the viral structural membrane proteins (S, M, E) follow the export route. Only protein S is equipped with a signal peptide to access the ER in the classical way. How the other two reach the ER is not yet clear. However, it is known that some membrane proteins also face the same problem in various organisms (Ott and Lingappa, 2002). The three viral membrane proteins follow the normal flow from ER towards the Golgi apparatus. In this process they are decorated with N-linked glycans, which is essential for proper folding and maturation of the molecules (Zhao et al., 2015). The three proteins assemble in the ERGIC/Golgi membrane and leading to its invagination. RNA-(protein N) complexes enter the pro-virions (Wolff et al., 2020), driven by N-M protein interactions, after which new virions containing S, M, and E proteins, as well as RNA-N complexes, pinch off by ERGIC or Golgi membrane fission. Finally, mature virions are released from the host cell in a non-classical manner. Instead of using the secretory exocytosis pathway, the virus utilizes lysosomes that are deacidified (possibly through the action of the viral protein ORF3a), concomitantly inactivating lysosomal degradative enzymes and disturbing cellular processes including autophagy, pathogen degradation and antigen presentation (Ghosh et al., 2020). During trafficking, the virions are continuously accompanied by KDEL-containing ER-chaperones GRP78/BiP and calreticulin and by the KDEL-receptor, which are also co-released. Hundreds of new virions may be excreted from an infected lung cell, which dies from exhaustion or is eliminated by the host’s immune system. The new virions can then infect other cells of the same host or be expelled in the air in droplets or aerosols that may invade another human host.

## The Spike Protein (S) Attaches the Virus to Host Cells and Mediates Internalization

The spike protein is an integral single-pass type-I membrane protein that protrudes in many copies from the outer surface of the virus, contributing its characteristic appearance. Spikes are responsible for binding the virus to a human or animal cell by recognizing specific receptors and, thereafter, for entry of the virus into the host cell. Spike proteins are the major antigenic determinants of the virus and the main targets in numerous active and passive immunization studies (Amanat and Krammer, 2020; de Alwis et al., 2020; Wrapp et al., 2020a). Indeed, there is an urgent need to generate neutralizing antibodies to fight Covid-19 (as Klasse (2014) explains: “*neutralization*“ is defined as “*the reduction in viral infectivity by the binding of antibodies to the surface of virions, thereby blocking a step in the viral replication cycle that precedes virally encoded transcription or synthesis*”). Detailed structural studies on the SARS-CoV-s have paved the way to understanding the complexity of the spike proteins and their way of action. Schematically, the spikes are built up as shown in Figure 1. Spike proteins are homotrimers. Each monomer has a large ectodomain that consists of several subdomains, followed by a transmembrane domain and an endodomain that contains a series of cysteine residues with palmitoyl chains attached (Petit et al., 2007; McBride and Machamer, 2010; Veit, 2012). S-palmitoylation is a well-known reversible protein post-translational modification (Charollais and van der Goot, 2009; Blaskovic et al., 2013). The ectodomain as well undergoes extensive post-translational modification and becomes heavily glycosylated by the host’s N-/O-glycosylation machinery (Fung and Liu, 2018; Watanabe et al., 2020a, b; Yao et al., 2020; Shajahan et al., 2020).

**FIGURE 2:**
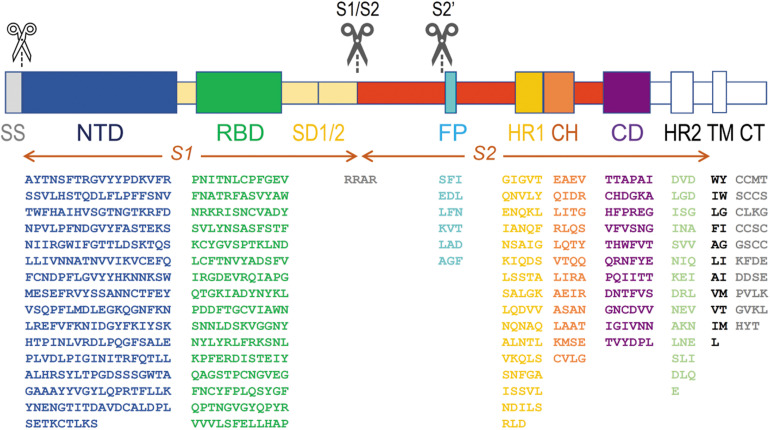
Architecture of SARS-CoV-s’ spike proteins. On top, the two S protein ectodomain halves (S1 and S2) with their different subdomains are depicted. Below, the amino acid sequence of the most important domains is shown for the most recent SARS-CoV-2 virus (sequence taken from the NCBI protein database, accession number YP_009724390). The subdomains from which the structure was resolved (PDB database, accession number 6VXX) are put in color, others are shown in white (or in gray, for the signal peptide). A white scissor indicates cleavage of the signal peptide in the ER of the host cell during biosynthesis of the protein. The two black scissors indicate the position of the consecutive cleavage steps occurring during viral infection. SS, signal sequence; NTD, N-terminal domain A^27^–S^305^; RBD, receptor-binding domain P^330^–P^521^; SD1, SD2 structural subdomains 1 and 2; S1/S2, place where cleavage occurs R^682^–R^685^; FP, fusion peptide S^816^–F^833^; HR1, heptad repeat 1 G^908^–D^985^; CH, central helix E^988^–G^1035^; CD, connector domain T^1076^–L^1141^; HR2, heptad repeat 2 D^1163^–E^1202^; TM, transmembrane domain W^1214^–L^1234^; CT, cytoplasmic tail C^1235^–T^1273^. Some residues are not seen in the structure in model 6VXX. In the NTD, the position of 71 residues (in 5 stretches, i.e., V^16^-P^26^, V^70^-F^79^, Y^144^-N^164^, Q^173^-N^185^ and R^246^-A^262^) is missing, and in the RBD, 30 residues remained undetermined (i.e., V^445^-G^446^, L^455^-L^461^, S^469^-C^488^ and residue G^502^). In the SD subdomain, residues P^621^-S^640^ are not seen. The peptide in which the S1/S2 cleavage occurs (containing the furin cleavage sequence RRAR) is also missing from Q^677^ till A^688^. From the small fusion peptide, the first twelve residues are presented in the structure (S^816^-T^827^), but the end of the peptide (L^828^ADAGF^833^) is missing as well till Q^853^. The last residue in the structure is S^1147^, somewhat before the HR2 subdomain.

The overall architecture of the SARS-CoV-s is well-understood and has been conserved during evolution of the viruses. It is summarized in Figure 2. Although the spike glycoproteins of the three SARS-CoV-s are structurally quite similar, their primary structures differ substantially (Table 1, and Supplementary Material: Supplementary Table 1, Supplementary Figure 2). Both the S1 and the S2 domains of the spike protein consist of a series of subdomains, each having a well-defined function. The N-terminal S1 is responsible for receptor binding, while the C-terminal S2 mediates membrane fusion to facilitate entry of the virus into a host cell.

**TABLE 1:** Properties of the three human Sars-CoV-s’ structural proteins S (ectodomain), M, E and N.

	SARS-CoV-2	SARS-CoV	MERS-CoV
**Membrane spike protein S**			
Identity (NCBI accession number)	YP_009724390	P59594	ASY99778
Ectodomain	V^16^–P^1213^	S^14^–V^1198^	Y^18^–W^1300^
Molecular mass (kDa)	132.924	131.577	141.544
Theoretical pI	6.30	5.56	5.65
Aliphatic index	83.23	82.63	81.61
Protein sequence SARS-CoV-2	–	76.0% *Id* – 17.0% *Si*	26.7% *Id* – 34.3% *Si*
SARS-CoV	76.0% *Id* – 17.0% *Si*	–	27.3% *Id* – 33.9% *Si*
MERS-CoV	26.7% *Id* – 34.3% *Si*	27.3% *Id* – 33.9% *Si*	–
**Membrane protein M**			
Identity (NCBI accession number)	QIC53216	AAP13444	AGH58718
Molecular mass (kDa)	25.146	25.070	24.552
Theoretical pI	9.51	9.63	9.27
Aliphatic index	120.86	115.06	103.70
Protein sequence SARS-CoV-2	–	89.2% *Id* – 8.1% *Si*	39.1% *Id* – 31.8% *Si*
SARS-CoV	89.2% *Id* – 8.1% *Si*	–	41.8% *Id* – 30.9% *Si*
MERS-CoV	39.1% *Id* – 31.8% *Si*	41.8% *Id* – 30.9% *Si*	–
**Membrane envelope protein E**			
Identity (NCBI accession number)	P0DTC4	AAP13443	AGH58723
Molecular mass (kDa)	8.365	8.361	9.354
Theoretical pI	8.57	7.01	7.64
Aliphatic index	144.00	145.92	111.59
Protein sequence SARS-CoV-2	–	96.0% *Id* – 4.0% *Si*	34.1% *Id* – 30.5% *Si*
SARS-CoV	96.0% *Id* – 4.0% *Si*	–	34.1% *Id* – 32.9% *Si*
MERS-CoV	34.1% *Id* – 30.5% *Si*	34.1% *Id* – 32.9% *Si*	–
**Soluble nucleocapsid protein N**			
Identity (NCBI accession number)	P0DTC9	AAP13445	AGG22549
Molecular mass (kDa)	45.625	46.025	44.857
Theoretical pI	10.07	10.11	10.05
Aliphatic index	53.52	49.81	56.08
Protein sequence SARS-CoV-2	–	89.3% *Id* – 8.1% *Si*	46.1% *Id* – 26.3% *Si*
SARS-CoV	89.3% *Id* – 8.1% *Si*	–	44.8% *Id* – 25.8% *Si*
MERS-CoV	46.1% *Id* – 26.3% *Si*	44.8% *Id* – 25.8% *Si*	–

*Values on the molecular properties of the viral structural proteins in this table were calculated using the program ProtParam (Gasteiger et al., 2005). For the spike proteins, only the ectodomains are considered. The aliphatic index is calculated as: X(Ala) + a^∗^X(Val) + b^∗^[X(Ile) + X(Leu)], in which X is the mole fraction of each of the 3 amino acids, and a = 2.9 and b = 3.9, i.e., the relative volumes with respect to Ala of Val (a) and Leu/Ile (b), respectively (Ikai, 1980). The primary structures of the proteins were compared using Clustal omega (Sievers et al., 2011) and resemblance between them is indicated under “protein sequence” as percentages identity (Id) and similarity (Si).*

### Overall Appearance of the SARS-CoV-2 Spike Glycoprotein Trimer: The “Pre-Fusion State”

**FIGURE 3:**
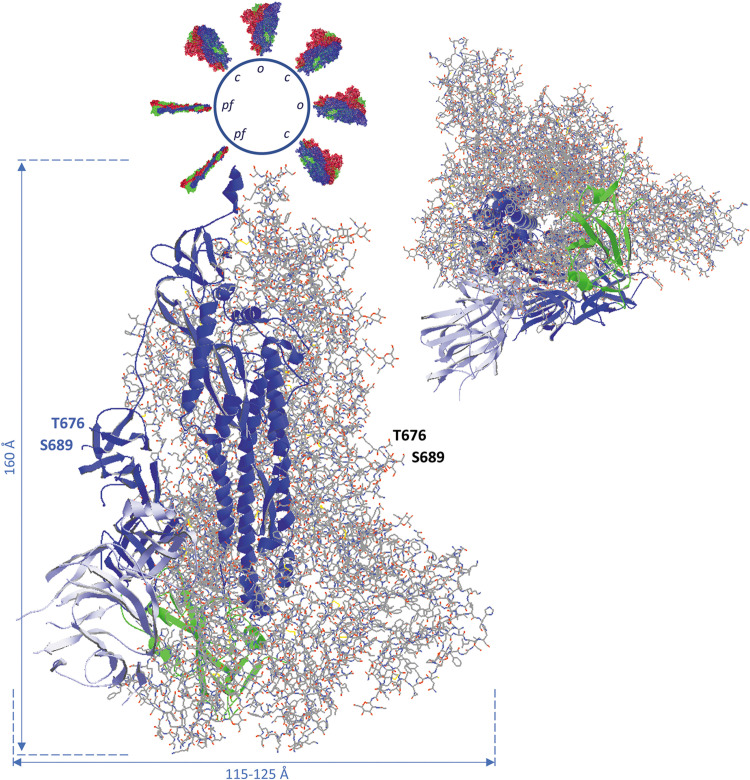
Side view (left) and bottom-to-top view with the virus particle being behind the spike protein (right) of the complete SARS-CoV-2 spike glycoprotein trimer, using coordinates from 6VXX.pdb. All subunits are in the closed (down) position. The B-chain is represented as ribbons, while A and C-chains are shown as backbone with side chains, in CPK colors (explained in the Supplementary Material). The B-chain ribbons are colored blue, with the NTD light blue and the RBD green. The location of the position where the activating proteolytic cleavage in the spike protein occurs is indicated for the subunit B (left, in blue) and for subunit A (to the right). The peptide of 12 residues in which cleavage takes place to generate spike protein molecules S1 and S2 (i.e., Q^677^TNSPRRARSVA^688^) is missing in the structure, but the flanking residues T^676^ and S^689^ are shown with their side chains and (manually) labeled. In the side view, the virion is at the top. It is represented as a sphere from which other spike proteins emerge. The latter are represented with their surface, either in the pre-fusion state with all subunits in closed state (*c*) and some of them with one subunit in open configuration (*o*), each subunit colored differently (chain A red, chain B blue, chain C green), or in the post-fusion state (*pf*; colored in the same way). Approximate spike dimensions were measured on the model and are indicated. In the open state, the spike protein length increases from 160 to about 175 Å. Literature reference for structural codes: 6VXX.pdb (Walls et al., 2020).

**FIGURE 4:**
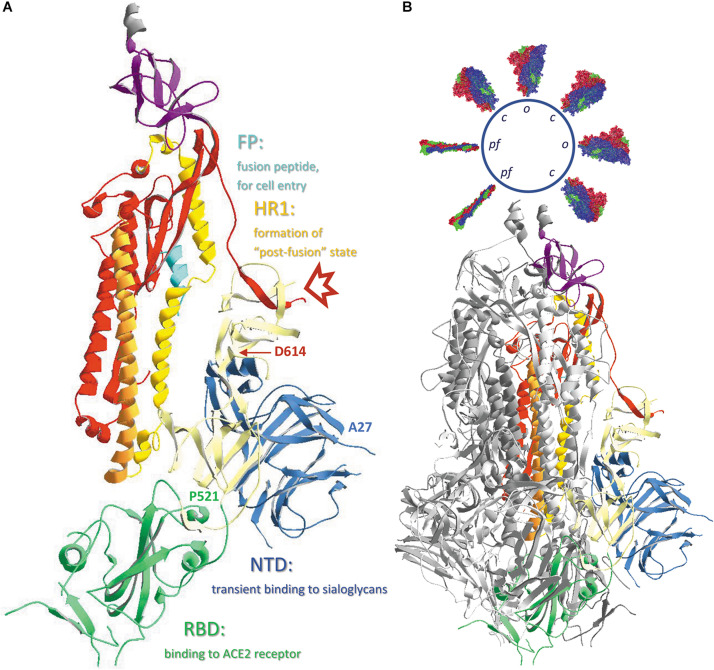
**(A)** The different subdomains of the SARS-CoV-2 spike protein, with their function. Chain B in model 6VXX.pdb is represented as ribbons colored according to the code used in Figure 2, i.e., the N-terminal domain (NTD) is blue, the receptor-binding domain (RBD) is green, the structural subdomains SD1 and SD2 are ivory colored, S2 is colored red, except from the fusion peptide (FP), which is turquoise, the first heptad repeat (HR1) that is in ochre and is immediately followed by the central helix (CH) in orange, and the connector domain (CD) is purple. The position where the cleavage S1/S2 occurs is marked with a red arrow and the positions of the N-terminal residue (A^27^) as well as the last residue of the RBD (P^521^) are indicated. Also, the position of residue D^614^ is indicated. **(B)** The same B-chain incorporated in the complete spike protein trimer. Chains A and C are shown as ribbons colored dark and light gray, respectively. Literature reference for structural codes: 6VXX.pdb (Walls et al., 2020).

A model of the complete SARS-CoV-2 spike glycoprotein using coordinates from 6VXX.pdb is shown in Figure 3. This structure starts at the twelfth residue of the mature polypeptide chain (A^27^) and ends at residue S^1147^. A spike protein subunit comprises 1173 residues (including the signal peptide of 15 residues). In the S1 half of the spike protein ectodomain we mostly find beta-strands, but the S2 part mainly consists of long alpha-helices. This figure (Figure 3) also indicates the end with which a spike protein trimer is attached to the virion (schematically drawn on top and decorated with multiple spike proteins). The organization of each of the spike protein subunits in different subdomains can be appreciated in Figure 4. In this figure, the B-subunit in model 6VXX is colored following the color code used in Figure 2. The last residue seen in the structure (S^1147^) lies just before the second heptad repeat (HR2). The position of an aspartate residue (D^614^) that very early spontaneously mutated to glycine (see section “The Much-d ebated lucrative spike protein mutant D^614^G”) is indicated as well in chain B.

### The Closed (Down) and Open (Up) Conformation of the Spike Protein

The viral spike protein responsible for binding to the host cell is initially in a “pre-fusion” conformation, in search for a receptor on a host cell. Thereby, each of the three subunits exists a certain period of time in a closed (or “down”) configuration, a state that is more stable but unable to bind the receptor (see section “A Viral Spike Protein Cannot Bind to the Ace2 Receptor When All Its Subunits Are in Closed Conformation”), and some time in a more unstable open (or “up”) configuration, which is receptor accessible. This hinge-like open ↔ closed transition is described in a publication by Wrapp et al. (2020b) and two very instructive videos of the internal movements in the SARS-CoV-2 spike trimer are included in the same publication on-line. When S1 successfully binds to its host cell receptor, the S protein structure becomes unstable and proteolysis may easily occur, resulting in shedding of the N-terminal S1 half of the molecule (S1/S2 cleavage). A second proteolytic cleavage (S2’) then takes place in the SARS-CoV-s, which further removes a long peptide up to just before the fusion peptide (FP), thereby fully exposing this small peptide. The virus is now ready to fuse its own membrane with the host cell membrane (as will be described in section “Events Causing Virus Entry Into Host Cells: The Spike Protein “Post-Fusion” State”).

In Figures 3, 4, all spike protein monomers are in the closed conformation. For the SARS-CoV-2 spike protein, a structure is available in which chain B exists in the open form. The two models 6VXX.pdb (all chains closed) and 6VYB.pdb (B-chain open) were uploaded in the database exactly in the same orientation, allowing direct comparison of both structures. Such a superposition of the B-chain in both models is seen in the Supplementary Material (Supplementary Figure 3). Similar events occur in the SARS-CoV and the MERS-CoV spike proteins. Coordinates may be found as well for MERS-CoV (5X5C.pdb: all chains down, and 5X5F.pdb: B-chain in up conformation).

### Comparison of the SARS-CoV-2, SARS-CoV and MERS-CoV Spike Protein Structures

**FIGURE 5:**
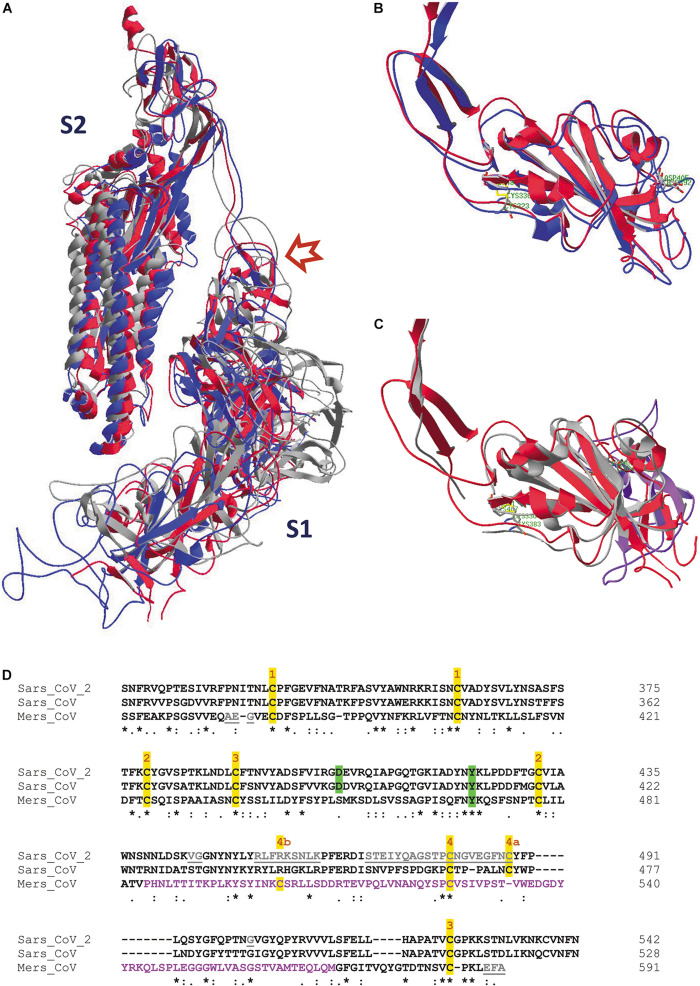
**(A)** One single chain is displayed of each of the spike protein trimer ectodomains from SARS-CoV-2 (6VXX.pdb), SARS-CoV (5XLR.pdb) and MERS-CoV (5X5C.pdb), after superposition of the three models (as explained in the Supplementary Material). They have been given different colors, i.e. red (SARS-CoV-2), blue (SARS-CoV) and gray (MERS-CoV). The upper left part shows the S2 halves of the chains, at the lower right are the S1 halves. The red arrow points to the place where the cleavage S1/S2 occurs. **(B)** The RBDs of one subunit of the SARS-CoV-2 (red) and SARS-CoV (blue) spike proteins were superposed, starting from the previous picture (Figure 5A). Side chains of residues C^336^ and C^361^ in SARS-CoV-2 (forming a disulfide bridge) and of residue D^405^ are also shown. They correspond with residues C^323^ and C^348^ (also forming a disulfide bridge) and D^392^ in SARS-CoV. These residues are labeled in green. **(C)** The RBDs of one subunit of the SARS-CoV-2 (red) and MERS-CoV (gray) spike proteins were superposed. Side chains of residues C^336^ and C^361^ in SARS-CoV-2 (forming a disulfide bridge) and of residue Y^423^ are shown as well. They correspond with residues C^383^ and C^407^ (also forming a disulfide bridge) and Y^469^ in MERS-CoV. These residues are labeled in green. The ribbons in the RBD stretch where major structural differences are observed with the SARS-CoV-2 protein (i.e., from V^484^ till M^569^ in the MERS-CoV sequence) are colored pink. The SARS-CoV-2 RBD is shown in figures B and C in roughly the same orientation. The SARS-CoV-2 RBD is shown from residue S^316^ till N^544^ in both figures, the SARS-CoV RBD from N^304^ till N^528^, and the MERS-CoV RBD from S^364^ till C^585^. A limited number of residues is missing in the three structures. **(D)** Multiple sequence alignment of the RBDs of the three spike protein sequences using Clustal omega (Sievers et al., 2011). The sequence where the structure of the MERS-CoV RBD diverts from that of the SARS-CoV and SARS-CoV-2 RBDs is shown in pink letters. The tyrosine that is conserved in the three RBDs, as well as the aspartate residue conserved in SARS-CoV and SARS-CoV-2, are highlighted in green. Disulfide bond cysteine residues are highlighted in yellow and numbers above them indicate their covalent interaction; 4a and 4b refer to different disulfide bond formation in SARS-CoV/SARS-CoV-2 and in MERS-CoV, respectively. Residues that are missing in the structures are in gray and underlined. Literature references for structural codes: 6VXX.pdb (Walls et al., 2020); 5XLR.pdb (Gui et al., 2017); 5X5C.pdb (Yuan et al., 2017)

High quality structural details of all three SARS-CoV spike proteins are available. However, each of them was uploaded in the PDB database in a different orientation, so they first need to be superimposed for comparison. After superposition of the individual RBDs we see that, especially for SARS-CoV-2 and SARS-CoV, the structures are very similar (Figure 5B). But, also from SARS-CoV-2 and MERS-CoV, the RBDs can be nicely superposed over an extended part of the domain (Figure 5C). Towards the end of the RBD, the protein fold in MERS-CoV starts diverting from the structure seen in SARS-CoV-2. This becomes also understandable when looking at a multiple sequence alignment of the three RBDs (Figure 5D). In the first part of the alignment (S^364^ till V^484^, MERS-CoV numbering), identities and similarities in the sequences are 19.5% and 37.4%, while in the second part (P^485^ till M^569^) the value for identities and similarities drops to 9.4% and 27.2%, respectively. Moreover, long gaps needed to be introduced to optimize the alignment. However, most of the disulfide bonds in the RBD are conserved in all three SARS-CoV-s (Figures 5B,C). In SARS-CoV-2, the stretch I^468^ till Y^489^ is missing, so the disulfide bond 4 is not evident from this structure. However, it is visualized in other structures of the SARS-CoV-2 RBD (see Supplementary Material, Supplementary Figures 4, 5). In MERS-CoV, the corresponding cysteine residue (in between T^533^ and V^534^) is absent in the sequence. But in this spike protein RBD, C^526^ forms an alternative disulfide bond with C^503^ instead.

### Spike Protein Glycosylation

N-glycosylation is a very ancient process that is fully conserved in all eukaryotes. The N-glycosylation process starts in the ER, during biosynthesis of the protein and its co-translational import in the ER lumen, by the covalent attachment of a pre-synthesized GlcNAc_2_-Man_9_-Glc_3_ precursor chain (Marth and Grewal, 2008; Aebi et al., 2009, Aebi, 2013; Stanley et al., 2017; Watanabe et al., 2019). After an elaborate quality control procedure in the ER (Ruddock and Molinari, 2006; Słomińska-Wojewódzka and Sandvig, 2015), during which the three glucose residues are removed, the protein that is now decorated with high-mannose chains is transported to the Golgi apparatus by ERGIC (Cop-II vesicles called ‘ER-Golgi Intermediate Compartment’). In the subsequent Golgi stacks, which move on by cisternal progression (Luini, 2011), all or some of the high-mannose chains may be enzymatically modified into complex-type or hybrid-type glycans (Strasser et al., 2014; Stanley et al., 2017; Wang et al., 2017).

For SARS-CoV-2 (Watanabe et al., 2020a), as well as for SARS-CoV and MERS-CoV (Watanabe et al., 2020b) the extent of N-glycosylation, as well as the maturation of the glycans in each of the NxT/S glycosylation sites, was thoroughly investigated. All three SARS-CoV-s’ spike proteins are heavily glycosylated over the whole length of their subunits. A SARS-CoV-2/SARS-CoV subunit has 22 potential N-glycosylation sites, while a MERS-CoV subunit has 23. All potential sites are also effectively glycosylated (though some scarcely not to their full extent), as is described in two publications by Watanabe et al. (2020a, b). In these two papers, the authors investigated the extent of glycan maturation in all these sites by analyzing glycopeptides with mass spectrometry. In each of the glycosylation positions, the glycan chains turned out to have been enzymatically modified in the Golgi apparatus to different extents, leading to very heterogeneous combinations of high-mannose-type (Man_9_GlcNAc_2_ to Man_5_GlcNAc_2_) and hybrid- and complex-type of glycan chains containing different numbers of antennae (A1 to A4), some of which are sialylated, with or without core fucosylation. It is worthwhile mentioning that most of the structural data available on the SARS-CoV-s’ spike trimers used proteins that were expressed in insect cells (details in Supplementary MaterialSupplementary Table 3), which produce glycan structures that differ from those in mammalian cells (Marth and Grewal, 2008). However, none of the pdb files shows any trace of covalently linked glycans. Besides being very heterogeneous, N-glycans are also extremely flexible structures, so that, except in a very few exceptional cases, they can only be modeled into a protein structure. Such modeling studies have been done for the SARS-CoV-2, SARS-CoV and MERS-CoV spike glycoproteins (Walls et al., 2019, 2020; Casalino et al., 2020; Grant et al., 2020; Vankadari and Wilce, 2020; Watanabe et al., 2020a, b; Zhou et al., 2020).

**FIGURE 6:**
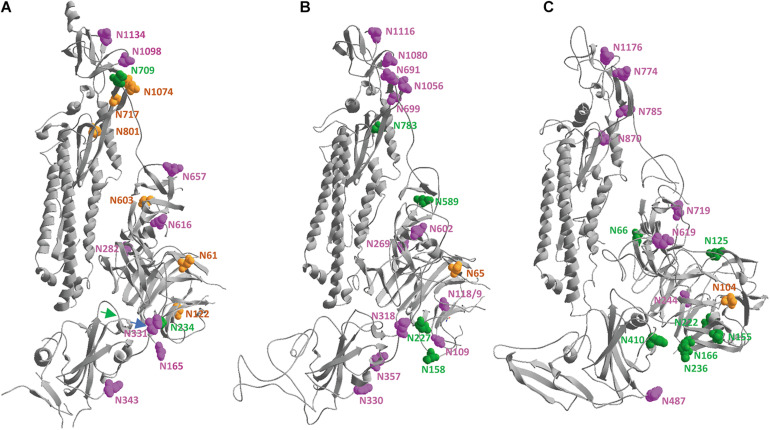
In the same spike protein ectodomains from SARS-CoV-2 **(A)**, SARS-CoV **(B)** and MERS-CoV **(C)**, after superposition of the three models as shown in Figure 5, the asparagine residues that are carrying N-glycans are highlighted by showing their vander Waals surfaces. They are given a color depending on the kind of glycan chain that is attached and following the color code introduced by Watanabe et al. (2020a, b), i.e., green, orange or magenta when the sugars are of high-mannose type in 80–100%, 30–79%, or 0–29% of the cases, respectively. The asparagine residues were numbered manually. A blue and a green arrow indicate the beginning and the end of the RBD, respectively.

In order to at least visualize where the N-glycans are attached to the spike proteins, the same subunits from SARS-CoV-2, SARS-CoV and MERS-CoV that were superposed and displayed in Figure 5A were used, to make sure that we are looking to the three subunits in the same orientation for comparison. Figure 6 shows that the glycosylation sites are nicely spread over the whole surface of the three SARS-CoV-s. Keeping in mind that N-glycans are very voluminous but also very flexible structures, it is obvious that the spike glycoproteins will be extremely well covered by an extensive glycan coat that may act as a real shield. Besides being important for protein folding and/or stability, this glycan coat might also hide epitopes and prevent antibodies from binding. Therefore, it may interfere with the host’s immune defense mechanisms. Finally, the published observations (Watanabe et al., 2020a, b) that many of the N-glycan chains are complex-type in nature tell us that the spike glycoproteins should have passed through the different stacks of the Golgi apparatus, where the glycan modifying enzymes are located in the correct order.

### SARS-CoV/SARS-CoV-2 and MERS-CoV Bind to Different Host Cell Receptors

Upon infection with SARS-CoV-s, the skin and mucosal membranes form the first layer of defense. Through the nose, eyes or mouth, the virus reaches the respiratory system where it may recognize a receptor on the surface of lung cells. The spike protein RBD (receptor-binding domain) is responsible for recognition and attachment to a host cell. The receptor for both the SARS-CoV-2 and the SARS-CoV spike proteins was identified to be ACE2 (angiotensin-converting enzyme-2), while the receptor for MERS-CoV is DPP4 (dipeptidyl-peptidase-4) (Belouzard et al., 2012; Fehr and Perlman, 2015; Li, 2016; Skariyachan et al., 2019; Hoffmann et al., 2020; Letko et al., 2020; Matheson and Lehner, 2020; Walls et al., 2020). DPP4, also known as CD26, is a single-pass type-II transmembrane protein of 766 residues with a very extended (738 residues) C-terminal ectodomain that is N-glycosylated. Thanks to its dipeptidyl-peptidase activity, it acts as a regulator of numerous physiological processes.

Angiotensin-Converting Enzyme-2 (ACE2) on the other hand is a proteolytic enzyme acting on angiotensin-I and -II, as well as on some other vasoactive peptides, and it is a regulator of blood pressure. It is a single-pass type-I membrane protein (805 residues) with an extended N-terminal ectodomain of 723 residues. Human ACE2 has six potential N-glycosylation sites and is heavily N-glycosylated (Warner et al., 2004). ACE2 is expressed in many different organs (Xu et al., 2020a), which might contribute to the damage to organs other than lungs in some Covid-19 patients. For SARS-CoV-s, it was observed that co-expression of a plasma cell membrane-anchored surface protease, TMPRSS2 (transmembrane protease-serine-2), highly facilitates cellular uptake of the virus (Hofmann and Pöhlmann, 2004; Heurich et al., 2014). Co-expression of ACE2 and the protease TMPRSS2 occurs not only in lung cells but in many different other cell types as well (Sungnak et al., 2020; Ziegler et al., 2020). TMPRSS2 is a single-pass type-II membrane protein of 492 residues with a C-terminal serine protease domain and seems to be involved in various physiological and pathological processes (Thunders and Delahunt, 2020). Its expression is developmentally regulated and increases with aging, which may contribute to the enhanced susceptibility of the elderly to SARS-CoV-2.

For SARS-CoV-s, a correlation has been observed between the grade of infection and the virus load received (Magleby et al., 2020), but also with the affinity of the viral RBD for the host receptor (Ou et al., 2020). It was shown that for SARS-CoV-2 the affinity is very high (with K_*D*_-values in the nM range), which contributes to the severity of the symptoms (Wrapp et al., 2020b). These affinities cannot be assessed from structural data but need to be measured by other experimental techniques, mostly based on ELISA or biosensor type of technologies (e.g., SPR or BLI) (for some examples: Huo et al., 2020; Ju et al., 2020; Pinto et al., 2020; Shang et al., 2020b; Shi et al., 2020; Walls et al., 2020; Wrapp et al., 2020a).

### Binding of SARS-CoV-2 to Its ACE2 Receptor

**FIGURE 7:**
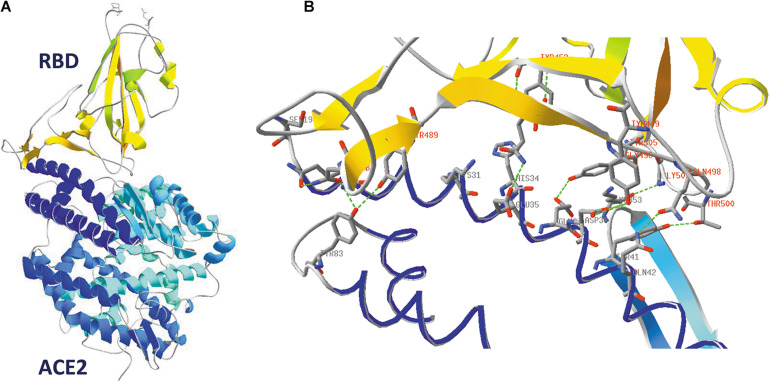
**(A)** The SARS-CoV-2 receptor-binding domain from 6VW1.pdb (chain E) on top (from N^334^ till P^521^; the side chains of both residues are added to the figure), with the ACE2 receptor protein (chain A) underneath. Ribbons are colored for secondary structure succession. **(B)** Interface between A-chain (ACE2 receptor) and E-chain (the SARS-CoV-2 RBD) in model 6VW1.pdb. A-chain and E-chain residues were selected that are within a distance of 3.2 Å from the opposite chain. Most of the selected A-chain residues belong to a long ACE2 helix (i.e., residues S^19^, Q^24^, K^31^, H^34^, E^35^, E^37^, D^38^, Y^41^, Q^42^), plus Y^83^ and K^353^. The E-chain residues are all located in the region between residue 449 and 505 (i.e. residues Y^449^, Y^453^, N^487^, Y^489^, Q^493^, G^496^, Q^498^, T^500^, G^502^ and Y^505^). Residues from the receptor are labeled in gray, while those from the viral RBD are labeled in red. Hydrogen bonds are shown as green dashed lines. The picture is shown in the same orientation as in A, but to make it clearer, the width of the α-helical structures was reduced in this figure to 1 Å. Literature reference for structural codes: 6VW1.pdb (Shang et al., 2020b).

A picture of SARS-CoV-2 RBD binding to its human receptor ACE2 can be made using the structure coordinates in model 6VW1.pdb. Figure 7A shows the SARS-CoV-2 RBD on top, with the ACE2 receptor molecule underneath. This figure shows both chains as ribbons colored for secondary structure succession, which enables to easily follow the progression in both chains from N- to C-terminus (alpha-helices and beta-strands are colored following rainbow colors and starting from blue to red, while loops are left gray).

When looking in detail to the interface between the two molecules (Figure 7B), it is clear that the RBD residues making contact with ACE2 are all located in the region from residue Y^449^ till Y^505^. This is precisely that part of the RBD that is very different in MERS-CoV (Figure 5) and explains why MERS-CoV is not using ACE2 as receptor molecule. All the residues lining the contact surface between SARS-CoV-2 and its ACE2 receptor are shown in Figure 7B (and Supplementary Figure 6, see Supplementary Material), together with hydrogen bonds that are formed between them. It is striking that it is precisely the RBD residue Y^489^, which forms a hydrogen bond with H^83^ from the ACE2 receptor, that was tentatively identified as one of the most mobile residues in the viral spike protein (see section “Flexibility in the Spike Glycoprotein”).

### A Viral Spike Protein Cannot Bind to the ACE2 Receptor When All Its Subunits Are in Closed Conformation

**FIGURE 8:**
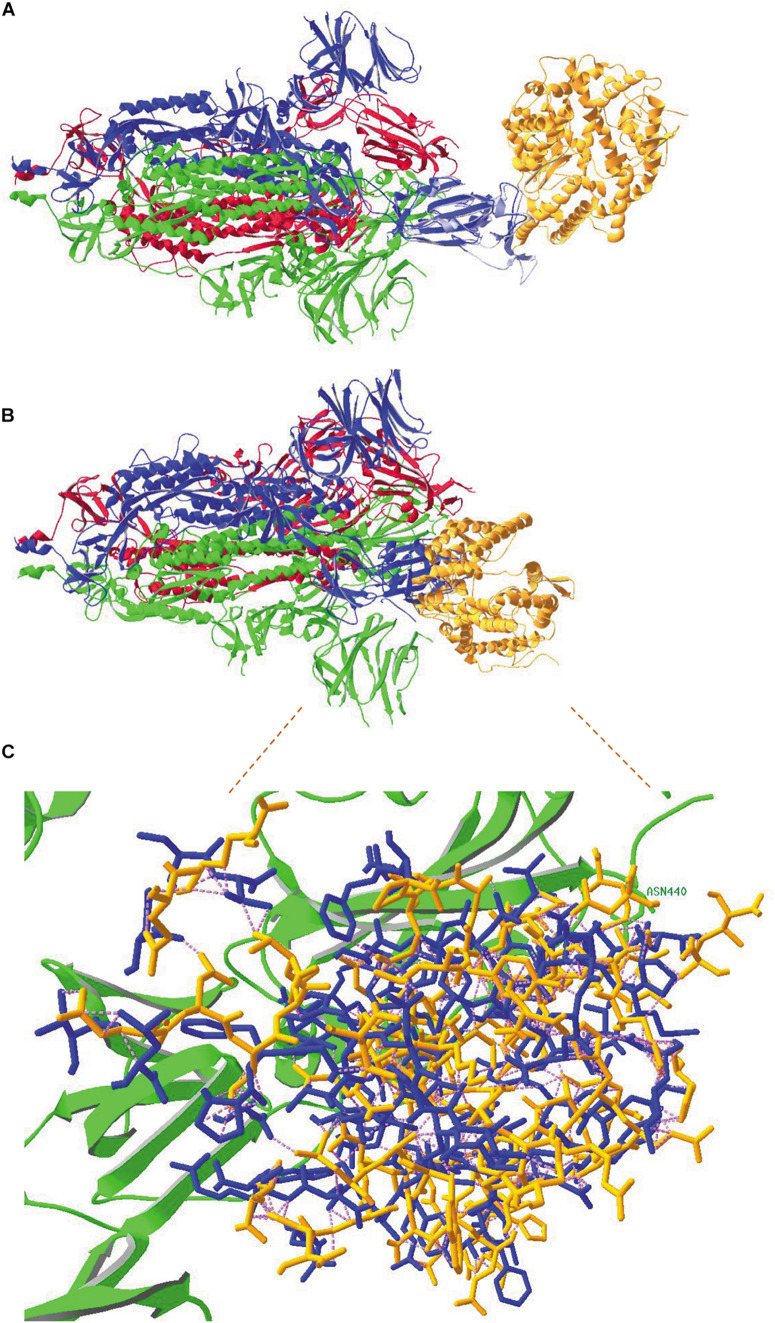
**(A)** An overlay was made between the SARS-CoV-2 RBD in model 6VW1.pdb (which shows binding of the virus to its ACE2 receptor) and the viral RBD of subunit B in model 6VYB.pdb (showing the spike protein trimer, subunits A and C in closed conformation and subunit B in open conformation). This figure shows the complete spike protein trimer (chains A, B and C with ribbons colored red, blue and green, respectively). The ribbons of the RBD in model 6VW1 are colored light blue, to demonstrate the overlay with the RBD from chain B in 6VYB. The ACE2 receptor protein is colored orange. There are obviously no clashes here. **(B)** An overlay was made between the SARS-CoV-2 RBD in model 6VW1.pdb (which shows binding of the virus to its ACE2 receptor) and the viral RBD of subunit A in model 6VXX.pdb (showing the spike protein trimer, all subunits in closed conformation; ribbons of chains A, B and C colored red, blue and green, respectively). Extensive clashes occur between the ACE2 receptor (colored orange), especially with the RBD of chain B. **(C)** A more detailed view of the clashes between the ACE2 receptor and the spike protein. The residues that are clashing are shown with their backbones and side chains, colored blue when they belong to chain-B and green when belonging to the C-chain of the spike protein (only one residue: N^440^, labeled), and orange when they belong to the ACE2 receptor. Clashes appear as pink dashed lines. How to make figures demonstrating the occurrence of clashes is explained in the Supplementary Material (Supplementary Figure 13). Literature references for structural codes: 6VW1.pdb (Shang et al., 2020b); 6VYB.pdb (Walls et al., 2020); 6VXX.pdb (Walls et al., 2020).

In order to visualize that a spike protein subunit needs to be in open conformation before it can bind the ACE2 receptor, an overlay was made between the spike protein RBD in model 6VW1.pdb and the RBD of one of the subunits of the spike protein trimer in model 6VYB.pdb. Figure 8A shows the full spike protein trimer that has one of its subunits in open state (chain B, of which the ribbons are colored blue), with a ACE2 receptor molecule (colored orange) bound to it. No clashes occur between the receptor and any of the spike protein subunits in this conformation. However, when the same exercise was performed using model 6VXX.pdb, which has all its subunits in closed state, obviously many clashes occur between the receptor molecule and spike protein subunits, as evidenced in Figure 8B. Clashing residues are highlighted in more detail in Figure 8C, where they are made visible as pink dashed lines.

### Flexibility in the Spike Glycoprotein

Another way of representing the spike protein is by coloring the model for “B-factor”. As discussed by T.E. Creighton already in 1993, B-factors (alternatively called temperature factors, or atomic displacement parameters, or Debye-Waller factors) describe the displacement of an atomic position from its average or mean position (Sun et al., 2019). B-factors (expressed in Å^2^) tell us, for each of the atoms in the model, how well determined and steady their position is. Several studies suggested that, in high quality models, B-factors might be used to identify flexibility and mobility in proteins, proposing that high B-factors indicate higher than average flexibility as opposed to low B-factors, which are believed to occur at more rigid positions.

Supplementary Figure 7 (see Supplementary Material) shows the SARS-CoV-2 spike protein trimer colored for B-factor. The region where we find residues having the highest B-factors corresponds to the receptor-binding domain, i.e., from residue P^330^ till P^521^ (from light green up to orange). The residue with the highest B-factor in each subunit is Y^489^. This high flexibility in the RBD may be thought to greatly assist the spike protein in finding a receptor on a host cell.

Spectacular flexibility in the spike protein is indeed described in two papers. One study (Ke et al., 2020) is based on cryo-EM and tomography to investigate the distribution of spike protein trimers and their flexibility using virus-infected VeroE6 and Calu-3 cells. Roughly 24 spike trimers were seen per virion, of which 97% in pre-fusion (about 31% of them with all monomers in closed state) and only 3% in post-fusion state (this state is described in section “Events Causing Virus Entry Into Host Cells: The Spike Protein “Post-Fusion” State”). The study showed that protruding spike proteins can extensively be tilted (up to 90°) towards the viral membrane. They seem to be rather sparsely but evenly distributed, without clustering, occurring at a density of about 1 trimer per 1,000 nm^2^ of membrane surface. Based on these calculations, it was hypothesized that multiple binding to ACE2 receptors, leading to avidity, will be an exception rather than the rule. A second study (Turoňová et al., 2020) is based on cryo-electron tomography, combined with molecular dynamics simulation. It shows that, in the pre-fusion state, the spike protein is extremely mobile and its stalk contains three hinges that were coined hip, knee and ankle (with estimated flexibilities of 16.5° ± 8.8°, 23° ± 11.7 and 28° ± 10.2°, respectively). This is assumed to give the head of the spike protein a lot of freedom and helps it to accurately scan the host cell for ACE2 receptors. Contrarily, in the post-fusion state the structure is apparently inflexible. Linked to this publication, a video demonstrates the pronounced flexibility in the pre-fusion state^1^. The extreme structural adaptability of the SARS-CoV-2 spike protein is also visualized in a publication presenting at least ten structures and transition phases occurring over the course of ACE2 binding and priming of the protein for membrane fusion (Benton et al., 2020).

### Endocytosis Is an Alternative Way to Enter Host Cells

SARS-CoV-s (but also other coronaviruses) may also invade a host cell by an alternative mechanism based on clathrin-mediated endocytosis. In this case, not only is the virus internalized but also the ACE2 receptor protein, which may lead to serious secondary effects due to reduced ACE2 activity (Delpino and Quarleri, 2020; Gheblawi et al., 2020; Lanza et al., 2020; Magalhaes et al., 2020; Ni et al., 2020; Samavati and Uhal, 2020; Saponaro et al., 2020). This occurs when there is no protease available nearby at the cellular surface to perform the necessary proteolytic cleavage into S1 and S2 (Heurich et al., 2014; Fung and Liu, 2019). Endocytosis is then followed by delivery of the virus inside an early endosome, which evolves towards a late endosome and finally towards a lysosome (Neefjes et al., 2017). The required proteolysis of their spike proteins then takes place in the context of either of these organelles, depending on which protease is able to perform the cleavage. Proteolysis leads to fusion of the virion (which is now inside the organelle) with the membrane of the respective organelle, followed by delivery of the viral RNA in the host cell’s cytoplasm by the same mechanism, involving the regions HR1 and HR2, together with the FP, as is explained in section “Events Causing Virus Entry Into Host Cells: The Spike Protein “Post-Fusion” State” (Burkard et al., 2014). Host proteolytic enzymes of the cathepsin family, comprising aspartic as well as cysteine and serine proteases with a broad substrate specificity, are occasionally mentioned to help priming SARS-CoV-s for membrane fusion, though this is sometimes disputed (Turk et al., 2012; Patel et al., 2018). Also, other viruses have been reported to rely on cathepsins at some stages of their life cycle (Brix, 2018).

### The Role of Spike Protein Cleavage by Furin and of Neuropilin-1 for SARS-CoV-2 Cellular Uptake

Contrary to all other coronaviruses known so far, SARS-CoV-2 acquired a furin-cleavage sequence, right within the peptide where cleavage occurs in the spike protein to remove the S1 half of the subunits (Figure 2). Furin is a single-pass type-I membrane protein that is ubiquitously expressed in vertebrates and has serine endoprotease activity. It cleaves at doublets or clusters of basic amino acids (e.g., KR↓ and RR↓), Rx(K/R)R↓ being the canonical cleavage sequence (Thomas, 2002). It occurs in the trans-Golgi network (where it cycles between sorting compartments), but also at the cell surface and in early endosomes, i.e., at all locations where the virus might pass by at the onset of infection.

Proteolytic cleavage of SARS-CoV-2 spike by furin results in exposure of the R^682^RAR^685^ sequence at the C-terminus of S1, thereby converting S1 into a so-called C-end-Rule (CendR) peptide. CendR peptides (conform to a R/KxxR/K motif, where the spacing of the basic residues is important), but not their cryptic motifs, are known to bind to neuropilin-1 (NRP1) and then get internalized, together with molecular structures that are attached to them, by a mechanism similar to but different from endocytosis (Teesalu et al., 2009). NRP1 is an essential pleiotropic surface receptor, present on endothelial and epithelial cells, acting as co-receptor molecule (Parker et al., 2012; Wild et al., 2012; Kumanogoh and Kikutani, 2013; Guo and Vander Kooi, 2015). It is a single-pass type-I membrane protein with five ectodomains essential for ligand-binding (two CUB domains, followed by two coagulation factor-homology domains, the first of which has a binding pocket for peptides with C-terminal arginine, and one MAM domain) and its short cytoplasmic domain interacts with PDZ-domain proteins. It is believed that, after furin cleavage but before further priming the spike by the secondary S2’ proteolytic step, S1 and S2 temporarily remain associated, giving time to the cleaved spike subunit to bind to NRP1. Two publications describe experiments showing that mAbs directed against NRP1, as well as a small molecule binding in the CendR pocket of NRP1, reduce SARS-CoV-2 infectivity, and also a mutant lacking the original furin cleavage site is less infective (Cantuti-Castelvetri et al., 2020; Daly et al., 2020). NRP1 can thus be considered as an important host factor facilitating cell entry of SARS-CoV-2 and explaining its enhanced infectivity when compared to SARS-CoV and MERS-CoV.

### The Potential Implication of the Glycocalyx and/or Host Lectins at the Onset of a Coronavirus Infection

When a virus invades a host, the first structure it encounters is the glycocalyx, a 50–200 nm thick layer made up as an intricate network of glycoproteins (N- and/or O-glycosylated) and proteoglycans (containing glycosaminoglycans, or GAGs) that are covalently attached to the outer surface of the plasma membrane, either by means of transmembrane domains or through GPI-anchors (Koehler et al., 2020). The glycan chains of these glycoproteins are often decorated with terminal sialic acids, while the GAGs contain extended chains of heparan-, chondroitin- or keratan-sulfate, these building blocks being heavily negatively charged. Numerous viruses are known to interact with these charged glycans, though in general with low affinity (K_*D*_-values in the mM range), leading to substantial binding strengths through multivalency. It is assumed that those initial interactions, mostly electrostatic in nature, bring the virions in close proximity and in elevated concentrations to the cell surface, increasing their chance to find their true receptors (Cagno et al., 2019; Koehler et al., 2020).

In some coronaviruses, the NTD, preceding the RBD, displays lectin activity and recognizes glycan ligands (Belouzard et al., 2012; Li, 2016). A finding that often goes unnoticed is that the MERS-CoV spike trimer as well binds to sialoglycans with the NTD proven to be responsible for that (Li et al., 2017). This binding is highly selective but of low affinity, and a multivalent sialoglycan presentation is required for interaction. It was argued that sialoglycans may guide MERS-CoV search for its true receptor, the DPP4, on the host cell surface. A similar mechanism of sialic acid recognition acting as an infection facilitator was proposed for other coronaviruses (Qing et al., 2020), including SARS-CoV and SARS-CoV-2 (Morniroli et al., 2020).

It was observed (Clausen et al., 2020) that the SARS-CoV-2 spike protein also binds heparan-sulfate (HS), which consists of linear chains of disaccharide building blocks comprising D-glucuronic acid (some of them modified to D-iduronic acid) and N-acetyl-D-glucosamine, extensively substituted with sulfate groups. Through modeling, the HS binding was pinpointed to the RBD, close to the ACE2 binding site, and both molecules bind independently from each other. Heparan sulfate (HS) binding is hypothesized to result from electrostatic interactions between the highly negative HS molecule and the overall positively charged RBD surface (see Supplementary Material, Supplementary Figure 8), the latter being able to accommodate a HS chain of up to 20 monosaccharides. Importantly, binding of HS promotes the RBD open conformation, thereby stimulating binding to ACE2 (Clausen et al., 2020). It was further discussed that the SARS-CoV-2 RBD surface is more electropositive than the one from SARS-CoV, mainly as the result of two mutations, i.e., T^431^→K^444^ and E^341^→N^354^.

Since not only do the SARS-CoV-s’ receptor molecules have covalently attached N-glycans, but also the SARS-CoV-s’ spike proteins are heavily glycosylated, it would not be unthinkable that the host’s own cellular surface lectins might be involved in capturing virions, or at least act as binding facilitators. C-type lectins (CLRs) are important receptors on patrolling myeloid cells that recognize glycans at the surface of foreign invaders, leading to the induction of immune responses. However, certain viruses have “learnt” how to modulate the response of macrophages and dendritic cells, and how to (mis)use them for promoting infection instead. Although detailed knowledge of mechanisms is still missing, it was hypothesized that capture by host lectins on myeloid cells does not always lead to normal antigen processing in the lysosomes followed by peptide presentation at the cell surface. Instead, the virions are temporarily contained, leading to their release at a later stage, followed by trans-infection of other susceptible target cells expressing the genuine ACE2 receptors (Geijtenbeek and van Kooyk, 2003). In case of SARS-CoV-2, preliminary reports using pseudovirus particles show that both DC/L-SIGN and MGL on antigen-presenting cells bind to spike protein glycan chains and promote virus transfer to permissive ACE2-containing cells (Thépaut et al., 2020). Moreover, the sialic acid-binding immunoglobulin-type lectins Siglec-3, -9 and -10 that are present on myeloid and/or B-cells were also found to bind to spike glycans (Chiodo et al., 2020).

### Binding of the SARS-CoV-2 RBD to the ACE2 Receptor Does Not Extensively Affect the RBD Conformation

**FIGURE 9:**
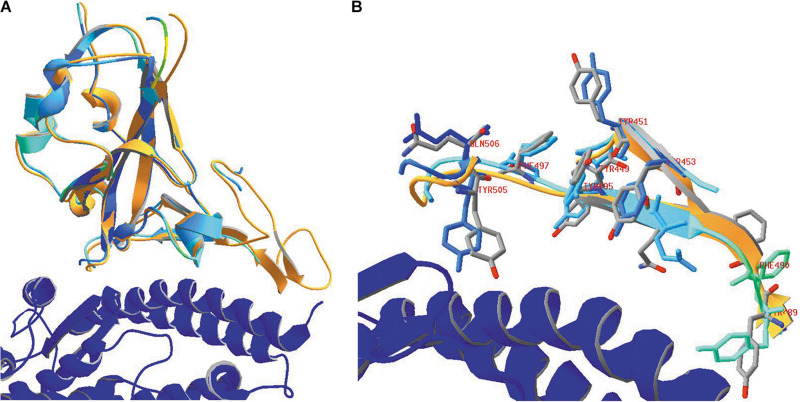
**(A)** The SARS-CoV-2 RBD (orange) in 6VW1.pdb (which was chosen as the reference model) is shown, together with the ACE2 receptor (blue). After superposing the RBD from 6VXX.pdb to the previous one, this domain is colored for RMS to analyze how well both RBD structures coincide. **(B)** Looking in more detail to some side chains in the vicinity of the ACE2 receptor. Side chains of the residues Y^449^, Y^451^, Y^453^, Y^489^, F^490^, Q^493^, Y^495^, F^497^, Y^505^, and Q^506^ are shown, labeled. They were given CPK colors in the reference model 6VW1, while they were colored for RMS in model 6VXX. Ribbons were colored orange in model 6VW1 and for RMS in model 6VXX. Literature references for structural codes: 6VW1.pdb (Shang et al., 2020b); 6VXX.pdb (Walls et al., 2020).

To compare the structure of the SARS-CoV-2 RBD in the absence (model 6VXX, chain B) and the presence of the ACE2 receptor molecule (model 6VW1, chain E), an overlay needs first to be made between the RBDs in both models. The result is shown in Figure 9A, in which the RBD of model 6VW1 is colored orange, while the ACE2 receptor is colored blue. Superposed is the RBD of model 6VXX, of which the ribbons are colored for RMS (RMS coloring, because the root-mean-square distances were calculated between corresponding backbone atoms to arrive at the color assignment for a group). RMS coloring for a model means that groups (backbones/side chains/atoms) in that model are colored according to how far they lie from corresponding groups in the other model that is considered the reference model (in this example, 6VW1 is taken as the reference). Regions that superimpose exactly are colored dark blue, with colors farther up the visible spectrum assigned for greater distances from corresponding atoms in the reference model. Figure 9A shows that the overall conformation in both models does not change dramatically.

In Figure 9B, we look in more detail to some amino acid side chains. In model 6VXX.pdb, the stretches N^450^-L^455^ plus Y^489^-Q^506^ from chain A are made visible (other residues are missing here). These peptides are very close to the ACE2 receptor (see above, Figure 7). In model 6VW1.pdb, the same stretches are added to the picture. The backbone plus side chains of these residues are shown in both models, with the aromatic residues and two glutamines, labeled. Finally, ribbons were added for the stretches on display. We may conclude that, locally, the orientation of some side chains is slightly modified in the model where the RBD is bound to the ACE2 receptor, which is not surprising, but the backbone is hardly affected.

### Only ACE2 but Not ACE Can Act as a Receptor for SARS-CoV-2

An homolog of ACE2 exists in humans, i.e., angiotensin-I-converting enzyme, or ACE. This enzyme is a peptidyl-dipeptidase, cleaving a dipeptide at the C-terminus of angiotensin, and is ubiquitously expressed throughout the human body (Riordan, 2003). Just as ACE2, ACE is also a membrane-bound protein. It consists of two very similar domains that originated by duplication, and of which the amino acid sequences can also easily be aligned with ACE2 (see Supplementary Figure 9 in Supplementary Material). Therefore, one could speculate on the possibility that ACE might also act as a receptor for SARS-CoV and SARS-CoV-2.

**FIGURE 10:**
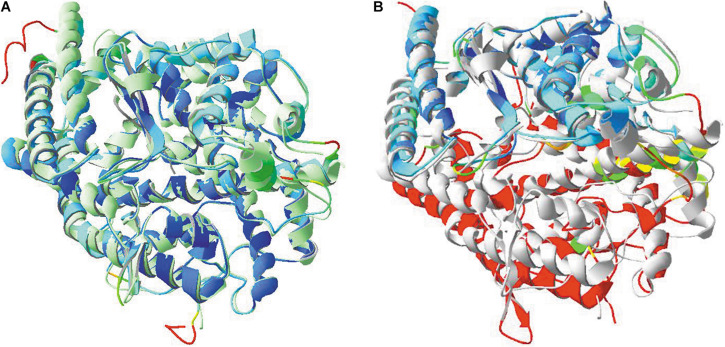
**(A)** Structural similarities between the two domains of ACE. The N-terminal domain of model 4BXK.pdb was used, together with the C-terminal domain of model 4APH.pdb. An overlay was made between both. Model 4APH was used as reference layer and ribbons in this model were colored light green. The ribbons of model 4BXK were colored for RMS. **(B)** Structural similarities between ACE2 (model 1R42.pdb) and the C-terminal ACE domain (model 4APH.pdb) and an overlay was made as well. Model 4APH was used as reference layer and ribbons in this model were colored light gray. The ribbons of model 1R42 were colored for RMS.Literature references for structural codes: 4BXK.pdb and 4APH.pdb (Masuyer et al., 2012; Douglas et al., 2014), 1R42.pdb (Towler et al., 2004).

In Figure 10, the overall structural similarities between both ACE domains and with ACE2 are visualized. The two domains of ACE are strikingly similar in structure, but also, the ACE2 structure is very similar to that of an ACE domain. However, though the three domains are structurally very similar, there are important differences to note in their primary structure (see Supplementary Material, Supplementary Figure 9). Particularly in the region of the contact surface of the ACE2 receptor protein with the SARS-CoV and SARS-CoV-2 RBDs, the sequences differ greatly from each other. Therefore, ACE is very unlikely to be able to act as receptor molecule for both viruses.

### Proteolytic Events Occurring in the Spike Glycoprotein Upon Binding to Its Receptor

**FIGURE 11:**
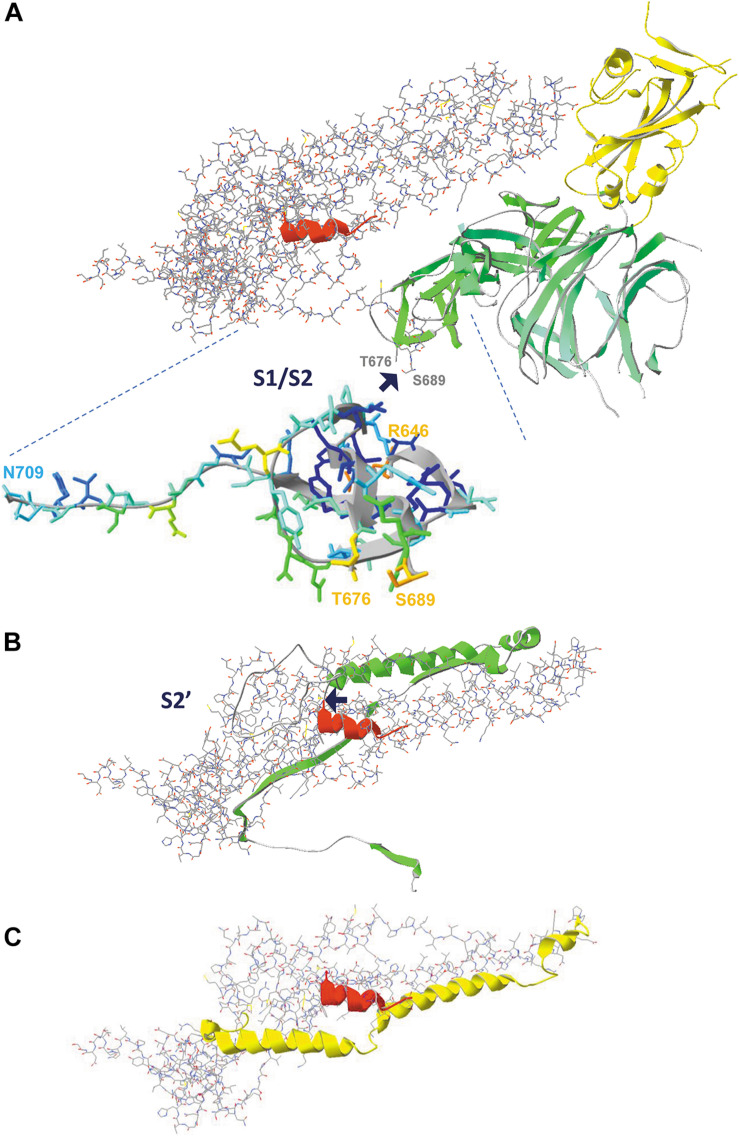
The consecutive proteolytic steps that occur upon binding of the spike glycoprotein to its ACE2 receptor. Pictures are made from model 6VXX.pdb. **(A)** The complete spike protein subunit (chain B), with the S1 half that is cleaved first shown as ribbons, colored for secondary structure succession, except for the RBD, which is colored yellow. The S2 half of the protein is shown as backbone and sidechains, except for the fusion peptide that is shown as ribbons and colored red. The peptide in which the cleavage occurs is missing in the structure, but the two flanking residues (T^676^ and S^689^) are labeled (manually, in gray). The enlargement shows the two peptides R^646^-T^676^ and S^689^-N^709^, colored for accessibility, with their start and end residues labeled (manually). The place where cleavage S1/S2 occurs is indicated with a blue arrow. **(B)** The figure in the middle shows the peptide that is removed by the second cleavage (proteolytic reaction S2’) as green ribbons. The place where cleavage occurs is indicated with a blue arrow. **(C)** The figure below shows what is left, with the HR1 domain (the heptad repeat 1, i.e. peptide G^908^-D^985^) now shown as ribbons (no backbone and side chains) and colored yellow. The HR2 domain is not part of the structure. After both cleavage reactions (S1/S2, followed by S2’), the remainder of the spike protein undergoes dramatic conformational changes highlighted in Figure 12. Literature reference for structural codes: 6VXX.pdb (Walls et al., 2020).

Soon after binding of the SARS-CoV-2 virus to its ACE2 receptor, a first proteolytic step occurs in the spike glycoprotein to split S1 (the N-terminal portion of the spike protein, containing the RBD) from S2 (the central and more rigid portion of the spike protein). The peptide of 12 residues in which cleavage takes place (i.e., T^676^ QTNSPRRARSVA S^689^) is missing in the structure, but the flanking residues T^676^ and S^689^ are clearly located at the outer surface of the spike protein trimer (Figures 3, 11). It can easily be imagined that this very hydrophilic peptide will be exposed and readily available for proteolysis.

In Figure 11A, we see the domains that are removed by the first cleavage as ribbons, colored for secondary structure succession, with the remainder of chain B in model 6VXX shown as backbone with side chains, and the fusion peptide (as far as its structure is available in the model) overlaid as red ribbons with the RBD domain at the top right (and colored yellow). After the first proteolytic cleavage, a second cleavage step (indicated as S2’) occurs just before the fusion peptide. In this step, the peptide shown as green ribbons in Figure 11B will be removed. Finally, what is left from the spike protein subunit is shown in Figure 11C. The heptad repeat (HR1) is important for the next events, which will lead to fusion of viral and host membranes, to allow entry of the viral RNA into the host cell.

### Events Causing Virus Entry Into Host Cells: The Spike Protein “Post-Fusion” State

After both proteolytic cleavage events (S1/S2, followed by S2’), the remaining S2 domain undergoes an instantaneous and dramatic change in conformation to adopt the “post-fusion” state. In this state, the coiled coil-forming heptad repeats (HR1 and HR2) of each of the three subunits in the trimeric S protein form a strong and extended six-helix bundle, which prepares the virion for membrane fusion with the host cell plasma membrane (Cai et al., 2020). Towards one end of this bundle, the three fusion peptides, one in each subunit, are now brought juxtaposed to the host cell membrane and catapulted into it, after which HR2 domains fold back to bring FP and the TM domain segments together, leading to fusion of the viral and the host membranes. This results in release of the viral RNA, decorated with N proteins, into the host cell (Shulla and Gallagher, 2009; Li, 2016; Cai et al., 2020; Shang et al., 2020a; Tang et al., 2020). It was shown that, due to differences in the HR1 domain sequences, SARS-CoV-2 has a significantly higher capacity for membrane fusion than SARS-CoV, which might also contribute to its higher infectivity (Xia et al., 2020).

**FIGURE 12:**
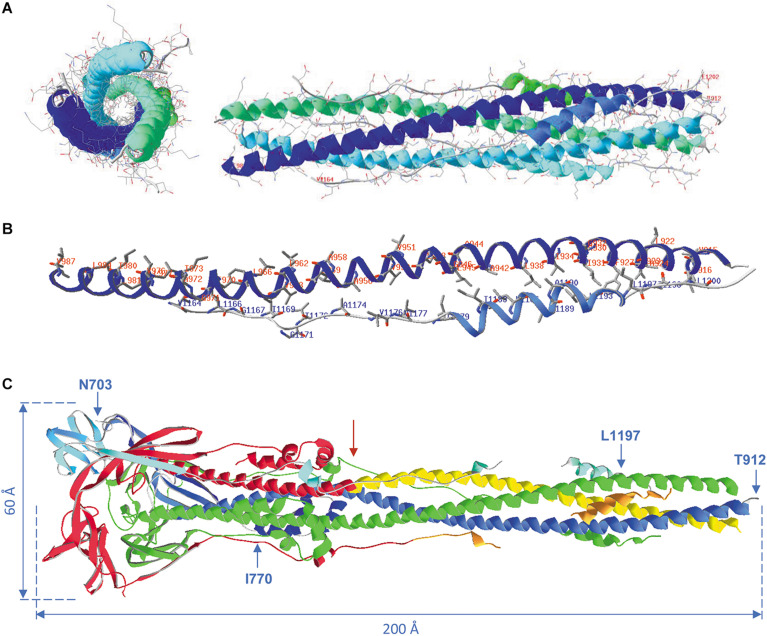
**(A)** Formation of the 6-helix bundle structure with the remaining heptad repeats HR1 and HR2. Pictures were made from model 6LXT.pdb. The structure is seen as a side view (right) and as a top view (left) with all side chains displayed. All backbones and side chains are displayed in CPK colors, plus ribbons colored for secondary structure succession. In the side view, the terminal residues of the long helices are labeled for both heptad repeats of the first spike protein subunit (i.e., HR1: T^912^-E^988^; HR2: V^1164^-E^1202^). **(B)** The HR1 and HR2 regions from the first subunit are shown with only the hydrophobic side chains, labeled in red for HR1 and in blue for HR2. The width of the ribbons was reduced to 1 Å to make the side chains on display better visible. **(C)** The post-fusion S2 trimer from model 6XRA.pdb. Residues available in this model are N^703^ till I^770^, T^912^ till N^1173^ (comprising HR1: T^912^-E^988^) and Q^1180^ till L^1197^ (the latter two stretches comprising part of HR2: V^1164^-E^1202^). The model is shown as ribbons, chain A colored green, chain B colored red, except the regions HR1 (yellow) and HR2 (orange) and chain C colored for secondary structure succession. The virion is at the right. The positions of residues N^703^, I^770^, T^912^ and L^1197^ in chain B are shown. Approximate spike dimensions were measured on the model and are indicated. Literature references for structural codes: 6LXT.pdb (Xia et al., 2020); 6XRA.pdb (Cai et al., 2020).

Figure 12A shows the formation of a 6-helix bundle structure, obtained for SARS-CoV-2 peptides, as a side and a top view. Hydrophobic interactions are the major force driving the formation of this helix bundle. Figure 12B shows HR1 and HR2, with only the side chains of the hydrophobic residues. In the peptide T^912^-E^988^, 34 residues out of 77 are hydrophobic in nature and in the peptide V^1164^-E^1202^, 18 out of 39 are hydrophobic (44% and 46%, respectively). They form two lines of hydrophobicity on these peptides that slowly twist around the long helices, which results in wrapping both HR regions around each other through the formation of an antiparallel coiled coil in each of the spike protein subunits. These regions are further assembled to form the 6-helix bundle structure. Figure 12C shows the post-fusion state, after removal of the S1 half of the spike protein. The right half of this structure, from residue T^912^ till the red arrow, is very similar to the structure of Figure 12A (as a side view). At one end, this structure is still attached to the virion (which is to the right), and somewhere, in between I^770^ and T^912^, are the fusion peptides (the peptides S^816^ till F^833^, missing in this structure) that will integrate in the host membrane, resulting in fusion.

### The Much-Debated Lucrative Spike Protein Mutant D^614^G

From February 2020 onwards, a point mutation (D^614^G) in the SARS-CoV-2 spike protein emerged (Korber et al., 2020). Within no time it supplanted the original protein worldwide and it was, and still is, wondered why this mutation spread at such an incredible speed. The apparently successful mutation was said to confer not only increased transmissibility to the virus, but also increased mortality. Studies using different kinds of pseudoviruses equipped with SARS-CoV-2 spike proteins indicated that spikes having the G^614^ mutation infect cells far more competently than the original D^614^ ones (summarized in Callaway, 2020b). Whether this will also be the case with the real virus in humans has yet to be confirmed, though it was further observed that genuine SARS-CoV-2 viruses were also more infectious in lab experiments on human lung cell lines and were present in increased concentrations in the upper airways of infected hamsters (Plante et al., 2020). These puzzling observations could neither to be attributed to a difference in numbers of virions produced, nor to an increased affinity of the variant to the ACE2 receptor (Daniloski et al., 2020). Certain studies ascribe the increased effectivity of the mutant to a decrease in premature S1/S2 cleavage of the G^614^ variant during assembly of new virions in the host (Daniloski et al., 2020; Zhang et al., 2020b), though an increased susceptibility to proteases was suggested as well from other experiments (Eaaswarkhanth et al., 2020; Hu et al., 2020b). The reason behind an alleged greater or lesser susceptibility to proteolysis remains unclear. Nevertheless, it needs to be kept in mind that mutations such as this one could influence the antigenic properties of the protein and might reduce the efficacy of vaccines that are currently under development using the original spike protein as it was isolated in Wuhan. Additionally, simple and seemingly harmless mutations may also have a pronounced effect on how the host immune system does recognize and react to the virus.

It was inferred from molecular modeling that the G^614^ mutation would destabilize the open conformation, thus promoting the closed state, which is unable to bind to the RBD (Becerra-Flores and Cardozo, 2020). Why then would the G^614^ mutant display a much higher fatality rate when compared to the original D^614^ protein? The authors suggested two possible hypotheses for these seemingly contradictory observations, i.e., the now more prevalent closed form might (i) be better shielded from attack by the host immune system, and/or (ii) elicit a harmful immune response, e.g., through the production of detrimental antibodies. Other studies, on the contrary, suggest that the D^614^G mutant rather loosens the spike protein and brings its subunits more easily in the open state, which should facilitate the binding to its ACE2 receptor (Mansbach et al., 2020). Either way, many contradictory conclusions are still circulating and the final word on this mutation has clearly not yet been said. A paper expressed the stand of affairs (August 2020) in its title as follows: “*Making sense of mutation: what D^614^G means for the Covid-19 pandemic remains unclear*” (Grubaugh et al., 2020) and that statement is surely still true today. Supplementary Figure 10 (see Supplementary Material) gives an impression of the surroundings of residue D^614^.

It needs to be stressed that mutations in the spike protein continue to emerge and by early May 2020, 329 naturally occurring variants were already reported (Li et al., 2020b), some of which make the virus resistant to certain monoclonal antibodies. Moreover, certain glycosylation deletions were found to reduce viral infectivity.

### The Problem of Antibody-Dependent Enhancement

There are clear indications that SARS-CoV-s also may infect certain cell types of the PBMC (collection of peripheral blood mononuclear cells) that do not express the ACE2 receptor, i.e., those belonging to the immune system (such as monocytes and macrophages, the former potentially also leading to productive virus replication). The immune response, which is specifically designed to clear infections, sometimes shows a dysregulated response leading to the opposite outcome (Taylor et al., 2015). This kind of response is due to the presence of anti-spike protein antibodies and known as antibody-dependent enhancement (ADE). This immunopathological situation is mediated by antibody Fc domains and occurs when virus-antibody immune complexes interact with cells carrying receptors for Fc. The ADE pathway is very complex with virus- as well as host-dependencies, and not all details are fully understood. Nevertheless, it is an important issue to be taken into consideration during development of vaccination strategies.

## Strategies to Prevent Binding of the Virus to Its Receptor

### Soluble Mutated ACE2 Analogs as a Decoy Receptor

The possibility of fooling the SARS-CoV-2 virus by administering high-affinity soluble ACE2 analogs as decoy receptors, thereby preventing virus binding to and entry in host cells by competition, was launched as an interesting idea to combat Covid-19 (Chan et al., 2020). The authors created an extensive library of 2,340 human sACE2 (soluble receptor) coding mutants that were expressed in human Expi293F cells (each cell expressing only one type of single mutant), which were then tested for SARS-CoV-2 RBD-binding using *in vitro* assays. Based on the results obtained, single mutants were combined and a series of sACE2 molecules with triple up to septuple mutations were generated for more detailed analysis. Several most interesting findings resulted from this study: (i) a mutation modifying residue T^92^, resulting in a sACE2 mutant that is not glycosylated anymore in position N^90^, favors RBD-binding, suggesting that the glycan at N^90^ hinders (but does not prevent) RBD-binding; (ii) a number of sACE2 mutants at the interface with RBD enhance binding, which opens perspectives for the aforementioned type of approach in fighting Covid-19; (iii) the variant called sACE2.v2.4 (carrying mutations T^27^Y, L^79^T and N^330^Y, thus still leaving the N-glycosylation site at N^90^ intact, and which is very well expressed and shows enzymatic activity on angiotensin II, albeit reduced), was purified and extensively analyzed: it was found to display a 65-fold higher affinity for immobilized SARS-CoV-2 RBD than the soluble wild type (using biosensor and ELISA technology) and also efficiently competes with antibodies from serum of Covid-19 patients for binding to the RBD. In Supplementary Figure 11 (see Supplementary Material) we are looking to the result of a proposed triple mutation T^27^Y, L^79^T, N^330^Y.

In another study, part of the sACE2 receptor (Q^18^-A^614^) was engineered after computational design and experimental affinity maturation, fused to the ACE2-collectrin domain (D^615^-S^740^) and dimerized by adding a human antibody Fc, resulting in avidity as well as long half-life times *in vivo*. A variant with seven amino acid changes (Q^18^R/K^31^F/N^33^D/H^34^S/E^35^Q/W^69^R/Q^76^R), and of which ACE2 enzyme activity was destroyed by a H^345^L mutation, was found to bind the spike RBD 170-fold more tightly than the wild-type ACE2. This (or some alternative) construct was proposed to be potentially useful as “trap” to neutralize SARS-CoV-2 and prevent viral entry into host cells (Glasgow et al., 2020).

In small-scale clinical studies, a human recombinant sACE2 molecule has already been used as a potential drug candidate with promising results (Zoufaly et al., 2020). These studies were based on earlier research using non-mutated hrsACE2 (human recombinant soluble ACE2) (Monteil et al., 2020).

### ‘Mini-Protein Inhibitors’ as Prophylactic Molecules and/or for Use in Therapeutic Treatments

Another interesting avenue in the search for prophylactic and/or therapeutic treatments of Covid-19 was published (Cao et al., 2020). In this study, researchers intended to find high affinity and thermostable mini-binders to the SARS-CoV-2 spike RBD that would compete with ACE2 receptor binding. Such molecules were devised both by incorporating the ACE2 long α-helix that interacts with the RBD (see above, Figure 7) in small proteins that were further designed to make additional interactions with the spike protein to enhance the affinity, as well as modeled from scratch. Such molecules would (i) not require obligatory storage at low temperatures, (ii) circumvent possible side effects inherent to using antibodies (e.g., ADE: see above), (iii) those peptides, being 20-fold smaller than antibodies, have a much higher binding site density per weight, (iv) potentially be applicable for internasal administration, e.g., as a gel or an aerosol, (v) make viral mutational escape very unlikely when being used in combinations. Promising peptides (56-64 amino acid residues long) were created that display excellent stability as well as high affinity for the spike protein (K_*D*_-values ranging from 100 pM to 10 nM) and they were found to prevent infection of Vero cells with an IC_50_ between 24 pM and 35 nM (Cao et al., 2020).

### Binding of Antibodies to the Spike Glycoprotein

Ever since the onset of the pandemic, numerous efforts have been made to track down neutralizing antibodies against SARS-CoV-2 that would help to combat the infection by using them in passive immunization. Initially, already available antibodies against SARS-CoV were tested for their potency against SARS-CoV-2 and later on, new antibodies were specifically generated and analyzed. A number of structural data have been made available in the PDB database. Antibodies in the pipeline are either of the conventional IgG type (or their Fab fragments), but also of the camelid type (heavy chain-only, or VHH, alternatively called single-domain antibodies). Because their concept is very different, both types of antibodies are conceived by Nature to recognize different types of epitopes: while classical antibodies are designed to grasp smaller groups or peptides sticking out from proteins’ surfaces by using their two extended antigen-binding regions as two scoops, camelid antibodies (from which “nanobodies” are derived) form rather “finger-like” structures to penetrate in cavities of the antigen (Romão et al., 2016; Jovčevska and Muyldermans, 2020). Nanobodies have several advantages, one being that, because of their limited size (only 15 kDa, which is ten times smaller than classical H_2_L_2_ antibodies), they can be administered as inhalable drugs, which for Covid-19 is an indisputable asset.

**FIGURE 13:**
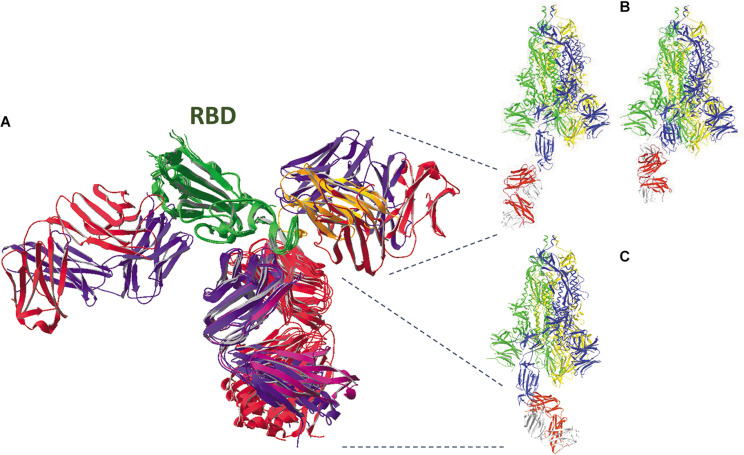
**(A)** An overlay was made of the SARS-CoV-2 spike protein RBD (from residues N^334^ till P^527^, colored dark green) with six antibody Fab fragments (all H- and L- chains are colored bluish and reddish, respectively) and one nanobody (colored orange). The Fab fragments in models 6XC2, 6XC4, 7BZ5, and 7C01 are pointing down, while Fab from model 6W41 (antibody CR3022) is pointing left, and the Fab from model 7BWJ is pointing right, which is overlapping with the nanobody from model 6Z2M.pdb. All these SARS-CoV-2-binding H_2_L_2_ antibodies, having neutralizing capacity, were cloned and expressed from memory-B cells present in PBMCs either isolated from Covid-19 recovered patients [mAb cc12.1 (CXC2.pdb) and mAb cc12.3 (6XC4.pdb): Rogers et al., 2020; mAb B38 (7BZ5.pdb): Wu et al., 2020; mAb CB6 (7C01.pdb): Shi et al., 2020); mAb P2B-2F6 (7BWJ.pdb): Ju et al., 2020] or from a convalescent SARS-CoV patient [mAb CR3022 (6W41.pdb): Yuan et al., 2020b]. The nanobody (H11-D4) was developed earlier against SARS-CoV and analyzed for its SARS-CoV-2 binding capacity (Huo et al., 2020). **(B)** Binding of antibody Fab fragment 7BWJ to the SARS-CoV-2 full spike trimer with chain B in open state (model 6VYB.pdb, left) and with all subunits in closed state (model 6VXX.pdb, right). An overlay was made between the structures (using the RBD of chain B) and no clashes were detected. **(C)** Binding of antibody Fab fragment 6XC4 to the SARS-CoV-2 full spike trimer with chain B in the open state (model 6VYB.pdb). An overlay was made between both structures (using the RBD of chain B) and no clashes were found. However, when chain B is also in the closed state, extensive clashes are seen with chain C residues (figure not shown; see Supplementary Material about how to detect clashes). In B and C, the ribbons of the spike protein subunits are colored yellow, blue and green for chains A, B and C, respectively, and red and gray for the Fab H- and L-chains, respectively.

Structures of SARS-CoV-2 RBD with various Fab fragments are available in the PDB database and they were used for making overlays. In Figure 13, six such Fab fragments and one nanobody are seen bound to the SARS-CoV-2 RBD. This figure was made after superposing all structures using the RBD available in model 6VW1.pdb as the reference chain.

Most of the antibodies analyzed compete for binding to the ACE2 receptor, as can be seen from Supplementary Figures 12, Supplementary Figures 13 (see Supplementary Material). When an antibody binds to the RBD, several clashes are seen with the ACE2 receptor. This is true for Fab fragments in models 6XC2, 6XC4, 7BZ5, and 7C01, which all bind to the same region of the RBD. This is also seen for the Fab fragment in model 7BWJ where some, though less prominent, clashes are observed. These five antibodies were described in literature to be neutralizing (Ju et al., 2020; Shi et al., 2020; Wu et al., 2020; Yuan et al., 2020b). Of course, to really compete with the ACE2 receptor for binding, the affinity of such an antibody for the spike protein is of utmost importance: when the affinity of the antibody is too low, the spike glycoprotein might nevertheless preferentially bind to the ACE2 receptor, leading to delivery of the viral RNA into the host cell’s cytoplasm.

Some structures are also available of a complete SARS-CoV-2 spike protein trimer with antibody Fab fragments. The Fab fragment of antibody S309 was determined to potently neutralize both SARS-CoV and SARS-CoV-2 (Pinto et al., 2020). Supplementary Figures 14A,B (see Supplementary Material) shows binding of three Fab molecules to the RBDs of each of the subunits of the SARS-CoV-2 spike protein trimer.

Another interesting antibody is CR3022, which was previously isolated from a SARS-CoV patient. It is directed against the RBD, but the epitopes to which it binds are different compared to the other antibodies (Figure 13). Using an *in vitro* assay, CR3022 proved to be neutralizing for SARS-CoV but not for SARS-CoV-2, though it is able to bind to its RBD, albeit with 100-fold lower affinity (Yuan et al., 2020a). The neutralizing effect of this antibody for SARS-CoV was explained through structural modeling: it was envisaged that the epitope to which CR3022 binds can only be reached by the antibody molecules when at least two RBDs are in the open conformation and, moreover, they need to be slightly rotated (Yuan et al., 2020a). Otherwise, there would be clashes with other parts (e.g., the NTDs) of the spike protein trimer (Supplementary Figure 16D, see Supplementary Material). It was further discussed in the same paper that, enigmatically, antibodies not having an *in vitro* neutralizing effect may nevertheless display *in vivo* protection for reasons that need to be further explored (Yuan et al., 2020a). Figure 13 also shows how some Fabs are only able to bind to a ‘one-up’ (6XC4), while another binds a ‘one-up’ as well as a ‘none-up’ spike trimer (7BWJ), and another needs more than one subunit in open state (6W41).

**FIGURE 14:**
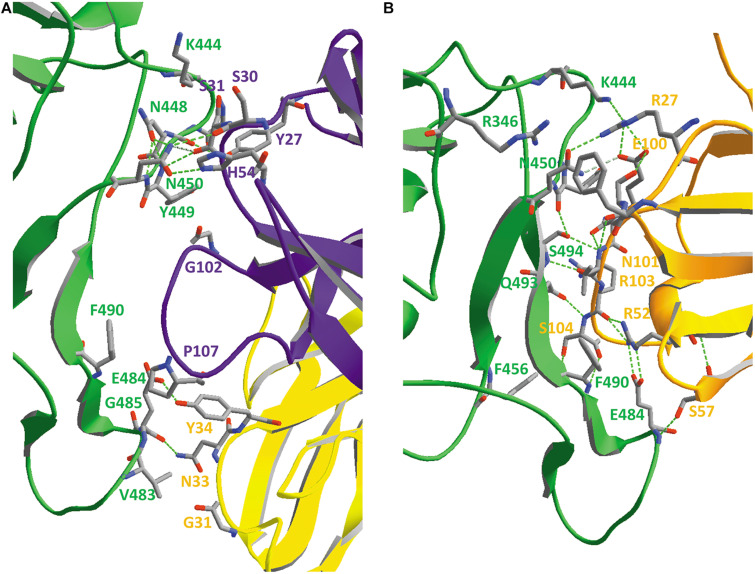
**(A)** Contact surface between the SARS-CoV-2 RBD (chain E, colored green) and an antibody Fab fragment from model 7BWJ.pdb, with the H- and L-chains colored purple and yellow, respectively. Amino acid residues that are within a distance of 3.5 Å from the opposing protein are shown with their backbone and side chains and were manually labeled. Hydrogen bonds are shown as green dashed lines; one hydrogen bond is colored gray because the distance between hydrogen donor and acceptor (3.32 Å) is slightly above the default maximal value of 3.20. **(B)** Contact surface between the SARS-CoV-2 RBD (chain E, colored green) and a nanobody (chain F, colored orange) from model 6YZ5.pdb. Amino acid residues that are within a distance of 3.5 Å from the opposing protein are shown with their backbone and side chains and were manually labeled. Hydrogen bonds are shown as green dashed lines; one hydrogen bond is colored gray because the distance between hydrogen donor and acceptor (3.33 Å) is again slightly above the default maximal value of 3.20. Literature references for structural codes: 7BWJ.pdb (Ju et al., 2020); 6YZ5.pdb (Huo et al., 2020).

Finally, when comparing binding of the Fab fragment to the SARS-CoV-2 RBD in model 7BWJ.pdb with that of a nanobody in model 6YZ5.pdb, the difference in the principle of antigen recognition between both antibodies catches the eye. As shown in Figure 13, both antibodies bind to the same region of the antigen. A more detailed picture of the binding is shown in Figure 14. Binding by the Fab fragment is due to residues belonging to two RBD loops, i.e., K^444^ till N^450^ and V^483^ till F^490^, which are grasped by the binding sites formed by the antibody H- and L-chains, respectively (Figure 14A). On the other hand, binding of the nanobody occurs because essentially two VHH loops, i.e., R^27^ till S^30^ and E^100^ till L^106^, fit into a shallow depression that is formed on the RBD between residues K^444^ till F^456^ and E^484^ till Y^495^ (Figure 14B). In the example of the Fab binding, seven hydrogen bonds are formed between the RBD and the antibody (five with the H- and two with the L-chain), while in the example of the nanobody the interaction is stabilized by eleven hydrogen bonds.

### Neutralizing Antibodies That Bind to the NTD Prevent Required Conformational Changes in the Spike Protein Trimer

Monoclonal antibodies with neutralizing activity were isolated from convalescent Covid-19 patients and characterized, some of which do not bind to the RBD, but rather to the NTD instead. From one of them (mAb 4A8), which binds with high (nM) affinity, the structure of the Fab in complex with the spike trimer was intensively analyzed (Chi et al., 2020). The potent neutralizing activity of this mAb was speculatively ascribed to its restraining effect on the conformational changes in the spike trimer, which are essential for activation of the spike leading to invasion of the host cell. In the Supplementary Material, Supplementary Figures 14C,D,E show a structure of the SARS-CoV-2 spike trimer in complex with three Fab fragments, each of them obviously binding to a different NTD and Supplementary Figure 15 visualizes the interface between the spike protein’s NTD and the Fab fragment.

## Other Viral Membrane Proteins

### The Abundant Membrane Protein M

Protein M, with a molecular mass of 24–28 kDa in various coronaviruses, is the most abundant protein in the viral membrane. It is known to be involved in the organization of viral assembly and binds to the nucleocapsid (Nal et al., 2005; Dhama et al., 2020). It is a multi-pass trans-membrane protein with three TM helices that are connected by short peptides, and with a long C-terminal endodomain. In the SARS-CoV and MERS-CoV, the N-terminal ectodomain is N-glycosylated at one single position (Fung and Liu, 2018). It has been suggested from electron microscopy and statistical analyses that protein M occurs in two conformations, which is supposed to regulate virus particle shape and size: an elongated structure that makes the membrane more rigid, with less curvature and high spike density, and a more compact one that renders the membrane more flexible and with less spike density (Neuman et al., 2011). However, there are no structural data available as yet for protein M from any of the coronaviruses. Therefore, we have to rely exclusively on predictions (see Supplementary Material, Supplementary Figures 17, 18). Properties of the protein are summarized in Table 1.

### The Minor Membrane Component Protein E

Protein E is the smallest of the SARS-CoV-s’ structural proteins, being 8.5–12 kDa in size, and its properties are summarized in Table 1. It has several functions, acting as an ion channel that is formed by homopentameric assembly of protein E subunits, but it is also involved in virus assembly and release, and interaction with the host (Yuan et al., 2006; Surya et al., 2018; Schoeman and Fielding, 2019; Dhama et al., 2020). Protein E is predicted to be a single-pass membrane protein with a short N-terminal peptide, followed by a TM helix and a longer C-terminal domain. However, the exact location of N- and C-termini is still a matter of debate (see Supplementary Material, Supplementary Figure 19). Some studies indicate that N- and C-termini might be located in the same compartment and it was also proposed that this protein might adopt different conformations in the viral membrane. Protein E from SARS-CoV was found to be S-palmitoylated at central cysteine residues (see Supplementary Material, Supplementary Figure 20). Very shortly after these cysteines are two potential N-glycosylation sites, N^48^ and N^66^, which were shown to be partially occupied (Fung and Liu, 2018). This would mean that, at least during biosynthesis, this part of the protein must face the ER lumen. MERS-CoV protein E, on the other hand, does not have N-glycosylation sites.

The structure of part (E^8^ till L^65^) of protein E from SARS-CoV embedded in LMPG (lyso-myristoyl phosphatidylglycerol) micelles was unraveled by NMR technology. The cysteine residues C^40^, C^43^ and C^44^ were replaced in the protein by alanines. All 16 models nicely coincide showing that there are no very flexible regions in the pentamer. The predicted transmembrane helices seem to form a central structure with the potential N-glycosylation sites at the outskirts (see Supplementary Material, Supplementary Figure 20). The side views suggest that, at least when protein E is taken up in micelles, the N- and C-terminal amino acid residues are located at the same side of the membrane, with the potential N-glycans and the palmitoyl chains at the opposite side.

## Wrapping Up the Viral RNA: The Soluble Nucleocapsid Protein N

Protein N (45–50 kDa) is the only soluble structural protein in the SARS-CoV-s. It is used by the virions to wrap up their RNA molecules (Chang et al., 2014; Dhama et al., 2020). It consists of two major domains that each contribute to RNA-binding: an N- and a C-terminal domain, the latter of which is used by the protein for dimerization. Both domains are linked to each other by a serine-arginine-rich peptide. A third domain at the C-terminus is important for interacting with protein M.

SARS-CoV and MERS-CoV protein N molecules are phosphorylated on serine and threonine residues at multiple sites, especially within the SR-rich peptide, by host kinases (Fung and Liu, 2018). Moreover, SARS-CoV protein N was proven to be modified by sumoylation (on residue K^62^) but the effect of this reaction needs further investigation (Fung and Liu, 2018). Finally, ADP-ribosylation also seems to occur in both SARS-CoV and MERS-CoV (Fung and Liu, 2018).

Supplementary Figure 21 (see Supplementary Material) shows that, despite the rather modest sequence similarity, both domains of protein N are structurally very similar in all three SARS-CoV-s. The dimeric C-terminal domains are attached with their N-terminal residues to the C-termini of two non-interacting N-terminal domains, making an extended overall structure. The C-terminal tails of the N-protein point in opposite directions. Both domains as well as the linker region have many basic residues, explaining the elevated theoretical pI value of 10, and the number of hydrophobic residues is very limited (the aliphatic index of protein N is very small: see Table 1). The excess positive charges on this protein are considered essential for wrapping up the polyanionic viral RNA. Protein-RNA interactions are proposed to be guided further by base stacking interactions using the protein’s aromatic residues that are amply present in the two RNA-binding domains: the YWF content amounts to 10.4% and 11% in the N- and C-terminal domains, respectively (see Supplementary Material, Supplementary Figure 22). Curiously, protein N is also predicted to have extended disordered regions (see Supplementary Material, Supplementary Figure 23), despite the fact that well-ordered structures were determined by X-ray crystallography. Only three regions (roughly residues G^99^-P^142^, A^217^-L^230^ and W^301^-Y^360^ in SARS-CoV-2) are predicted by the program IUPred to be ordered, i.e., some parts of the N- and C-terminal domains and part of the SR-rich peptide. It has been discussed (Chang et al., 2014) that inclusion of disordered regions (IDRs) within the structured regions of protein N not only increases the binding affinity for nucleotides, but also its binding cooperativity (making a next domain binding better and stronger). This may be explained in the light of the following known IDR’s properties: enhanced binding/speed of interaction; promiscuity in binding partners; enabling larger interaction surfaces with partners upon complex formation (the IDR is wrapping itself tightly around its binding partner); facilitating introduction/removal of post-translational modifications (Tompa, 2012; Habchi et al., 2014).

## Discussion

This paper summarizes and discusses the current knowledge of the structural proteins that make up the coronaviruses in general, and the beta-coronaviruses SARS-CoV-2, SARS-CoV and MERS-CoV in particular. We demonstrate how these proteins are well-designed by Nature for their function, how they cooperate with each other to make very successful virions, and how these viruses mislead and hijack the host for their own benefit. Certain aspects are well-known since they are explained and illustrated in other papers, but some others remain often unnoticed or their importance underestimated, such as, for instance, the observation that coronaviruses might transiently interact with sialoglycans/heparan-sulfate prior to binding to their true receptors, thereby facilitating and speeding up invasion of a host cell. Another point is that these beta-coronaviruses developed very different ways of entering a host cell, i.e., either by directly releasing their RNA after membrane fusion, or after invading the host cell making use of the endocytotic pathway (occasionally with the help of NRP1), and in all routes they rely on the action of a plethora of host proteases that are ubiquitously available. These invasion routes exist side-by-side, and some virions may take one route, while others, at the same time and in the same host, may take the other. The way by which new virions leave a host cell through de-acidified lysozomes is also peculiar. Furthermore, it is of the utmost importance to keep an eye on new mutants that may develop in the future, which might turn these SARS-CoV-s into even smarter particles than they already are today, possibly rendering our developed defense strategies ineffective. Finally, although already a lot is known about these SARS-CoV-s, at several points knowledge and essential details are still missing. It is hoped that the near future will see these gaps being filled in, and that smart solutions, maybe still not been considered today, will emerge that will put an end to the pandemic that is currently straining the health systems globally. Very promising strategies to combat Covid-19 are in the pipeline, amongst others, the development of decoy receptors and mini-protein inhibitors, monoclonal antibodies and nanobodies that might find applications in nasal sprays, new and repurposed antivirals, and, of course, vaccines. Today, and after only one single year of development and clinical trials, four vaccines have already received approval in EU/United Kingdom and United States and are now being successfully applied. Two of them are viral vector vaccines in which recombinant DNA is packaged in a harmless adenovirus, either from chimpanzee (Oxford-AstraZeneca) or of human origin (Johnson and Johnson). Two others apply newer technology, based on a synthetic piece of mRNA packaged in lipid nanoparticles (Pfizer/BioNTech and Moderna) (Kaur and Gupta, 2020; Silveira et al., 2021). All four use coding sequences for the spike protein. The latest developments will certainly also contribute to our fight, not only against other types of viral infections, but also against cancer (Pardi et al., 2018; Zhang et al., 2019; Xu et al., 2020b; Miao et al., 2021).

The problematic spreading of human coronaviruses early in this century, with SARS-CoV, MERS-CoV and the most recently developed SARS-CoV-2 as known culprits, unmistakably ushered a huge variety of structural studies dealing with all aspects of these viruses. This will be even more so if new pandemics emerge in the near or more distant future, a situation that is predicted by many researchers and healthcare workers to occur. Consequently, it is of the utmost importance to understand these structures and to be able to look at them in detail, using a combination of a series of bioinformatic tools, most of which are freely available these days through the internet. It is hoped that this publication will stimulate more researchers and students to visualize the available structures on their computers and to use the bioinformatic tools that become available, which will help advance science in this and related fields. With this goal in mind, we present in the Supplementary Material a set of guidelines, using the interactive program DeepView (Guex and Peitsch, 1997; Guex et al., 2009), that allows non-specialists in structural biology to upload protein structures and scrutinize them. This program was also used to make the figures in this paper.

## Epilog: Recent Developments

Circulating SARS-CoV-2 lineages were estimated to accumulate nucleotide mutations, mostly synonymous, at a rate of about 2.7 per month (Duchene et al., 2020). However, at least three examples of much faster mutation rates have recently emerged, one in United Kingdom (Rambaut et al., 2020), another one in South Africa (Tegally et al., 2020) and a third one in Brazil (Faria et al., 2021). The United Kingdom variant has nine amino acid mutations in the spike protein when compared to the original Wuhan strain, one of which in the RBD (N^501^Y), while the SA and Brazilian variants display each ten non-synonymous mutations in the spike protein, three of which in the RBD (K^417^N, E^484^K, N^501^Y in the South African variant and K^417^T, E^484^K, N^501^Y in the Brazilian one). Although the reason for this rapid development remains enigmatic so far, intra-host evolution in an immune-deficient or immune-suppressed individual suffering from long-term infection was suggested, possibly leading to an accumulation of “immune-escape” mutants. A major problem is that such mutations might affect the efficacy of vaccines being developed to combat Covid-19. The variant strains are analyzed in Supplementary Figures 24–26 (see Supplementary Material).

## Author Contributions

SB and EV performed in-depth literature searches on the topic. SB wrote the first draft of the manuscript and made the analyses and the figures and tables in this manuscript, which is partially based on a 30-h course “Bioinformatic Tools” that the first author has been teaching at Vrije Universiteit Brussel in the “Master of Science in Molecular Biology” study program since 2010. Both authors contributed to manuscript revision and fine-tuning and approved the submitted version.

## Conflict of Interest

The authors declare that the research was conducted in the absence of any commercial or financial relationships that could be construed as a potential conflict of interest.

## Footnotes

1 https://www.pei.de/EN/newsroom/press-releases/year/2020/14-new-findings-spike-protein-sars-cov-2.html

## Abbreviations

ACE: Angiotensin-I-Converting Enzyme
ACE2: Angiotensin-Converting Enzyme-2
ADE: Antibody-Dependent Enhancement
BLI: Bio-Layer Interferometry
CD: Connector Domain
CD26: Cluster of Differentiation-26
CendR: C-end Rule
CH: Central Helix
CLR: C-type Lectin Receptor
Covid-19: Coronavirus disease-2019
CPK: “Corey-Pauling-Koltun”, type of atom coloring
CT: Cytoplasmic Tail
CUB: C1r/C1s/Uegf/Bmp1 kind of protein domains
DC/L-SIGN: Dendritic Cell/Liver-Specific ICAM, *inter-cellular adhesion molecule)*-3-Grabbing Non-integrin
DMV: Double Membrane Vesicle
DPP4: DiPeptidyl-Peptidase-4
dsRNA: double-stranded RNA
ELISA: Enzyme-Linked Immuno-Sorbent Assay
EM: Electron Microscopy
EMBL-EBI: the European Molecular Biology Laboratory–European Bioinformatics Institute
ER: Endoplasmic Reticulum
ERGIC: ER-Golgi Intermediate Compartment
ExPASy: Expert Protein Analysis System, the bioinformatics resource portal operated by the Swiss Institute of Bioinformatics
Fab: antibody’s antigen-binding Fragment
Fc: antibody’s crystallizable Fragment
FP: Fusion Peptide
GAG: Glycos-Amino-Glycan
Glc: D-Glucose
GlcNAc: N-Acetyl -D-Glucosamine
GRP78/BiP: Glucose-Regulated Protein, 78 kDa/Binding immunoglobulin Protein: two alternative names for an ER-Hsp70 chaperone
HR1, HR2: first, second Heptad Repeat
HS: Heparan-Sulfate
IDR: Intrinsically Disordered Region
K_*D*_: Dissociation constant
mAb: monoclonal Antibody
MAM: Meprin/A5-antigen/ptp-Mu kind of protein domains
Man: D-Mannose
MERS-CoV: Middle-east respiratory syndrome Corona Virus
MGL: Macrophage Galactose-type Lectin
NCBI: National Center for Biotechnology Information, U.S. resource for bioinformatics tools and services
NRP1: Neuropilin-1
Nsp: viral Non-structural protein
NTD: N-Terminal Domain
NxT/S: consensus sequence for N-glycosylation : Asn-x-Thr or Asn-x-Ser
ORF: Open Reading Frame
PBMC: Peripheral Blood Mononuclear Cells
PDB: Protein Data Bank
PDZ: PSD-95/Dlg/ZO-1 kind of protein domains
RBD: Receptor-Binding Domain
RdRp: RNA-dependent RNA polymerase
RMS: Root Mean Square
RNP: Ribonucleoprotein
S1: N-terminal half of Spike protein
S1/S2: first cleavage site
S2: C-terminal half of Spike protein
S2’: second cleavage site
SARS-CoV: Severe acute respiratory syndrome Corona Virus
SARS-CoV-2: Severe acute respiratory syndrome Corona Virus-2
SD1, SD2: Structural sub-Domains 1 and 2
Siglec: Sialic acid-binding ImmunoGlobulin-type LECtins
SPR: Surface Plasmon Resonance
SS: Signal Sequence
ssRNA: single-stranded RNA
TM: Trans-Membrane domain
TMPRSS2: Trans-Membrane Protease-Serine-2), UniProtKB, Universal Protein Knowledge Base, database of protein sequences and functional information).
