# Supplementary material for: Scrutinizing Coronaviruses Using Publicly Available Bioinformatic Tools: The Viral Structural Proteins as a Case Study
Source: Front Mol Biosci. 2021 May 24;8:671923. doi: 10.3389/fmolb.2021.671923 (PMC8181738; doi:10.3389/fmolb.2021.671923)
Supplement: Supplementary file 1 [file Data_Sheet_1.pdf]

Scrutinizing Coronaviruses Using Publicly Available Bioinformatic Tools: The Viral Structural Proteins as a Case Study

Sonia Beeckmans and Edilbert Van Driessche*

Research unit Protein Chemistry, Vrije Universiteit Brussel,
Pleinlaan 2, 1050 Brussels, Belgium
* Sonja.Beeckmans@vub.be

Supplementary Material

Illustration of the use of bioinformatic tools,
with reference to the interactive molecular graphics program DeepView

An overwhelming amount of new data in the field of molecular biology/biotechnology and health sciences research relies on structural data that accumulates at an incredible speed. As such, mastering bioinformatic tools, as well as being able to look 'inside' macromolecular structures using a personal computer became essential for planning new experiments and understanding science in all its aspects. Several open-source programs are available that allow visualizing structures on a computer/laptop screen. The program chosen here is Swiss-PdbViewer, also called DeepView. It is a strong interactive molecular graphics program for viewing, as well as for analyzing, biological (macro)molecules (proteins, polynucleotides and smaller or larger heterogroups) on a computer/laptop screen. DeepView was developed at the Swiss Institute of Bioinformatics and is available at ExPASy (https://www.expasy.org/) for a variety of computer platforms (Windows, Mac-OS-X or Linux) (Guex and Peitsch, 1997, Guex et al., 2009). The DeepView program offers several advantages:

- it generates structures from different data files from the PDB (protein data bank) simultaneously and offers the possibility of superimposing them;
- it allows to measure distances and angles, compute energies and perform energy minimization;
- it is very good in simulating the effect of mutations/modifying structures;
- it allows coloring of structural elements in many different ways to emphasize interesting molecular features;
- it produces very good (publication-quality) images;
- it can do sophisticated modeling (being linked to the Swiss-Model program);
- it computes molecular surfaces and electrostatic potentials, and detects cavities;
- it performs homology modeling;
- it allows at all times to save the current working environment;
- very easy-to-use instructive manuals and tutorials for the program are available.

The current Supplementary Material accompanies the main paper, which reviews the present knowledge on the structural proteins of the human coronaviruses SARS-CoV-2, SARS-CoV and MERS-CoV. Here we explain how figures such as the ones shown in the paper and those below can be generated. It is most instructive to read this tutorial while sitting at the computer and using the DeepView program. Pdb files can be downloaded from the NCBI website at: https://www.ncbi.nlm.nih.gov/protein/. Most entries contain multiple polypeptide chains and in the following sections, the chains used to make a figure are indicated. Besides

DeepView, reference is also made to other open-source bioinformatic tools that help understand structures and properties of macromolecules involved.

Literature references for all pdb files used to make the figures are mentioned in **Table 3** at the end of this Supplementary Material.

THE PROGRAM DEEPVIEW

The DeepView program may be downloaded from the ExPASy site at: http://www.expasy.org/spdbv/ (Version 4.10). In the different sections below, its use will be explained for getting a better understanding of the structural proteins from the beta-coronaviruses SARS-CoV-2, SARS-CoV and MERS-CoV. For basic manipulations to use this program the reader is referred to two instructive manuals that are available at:
https://spdbv.vital-it.ch/Swiss-PdbViewerManualv3.7.pdf
https://spdbv.vital-it.ch/TheMolecularLevel/SPVTut/

The DeepView program uses, by default, a dark blue background, which is perfect for scrutinizing the models on a screen but is not very convenient for publications and for printing. Therefore, some of the default parameters (in the Toolbar, under 'Prefs' > 'Molecule Color Settings') were modified to make the figures. Besides making the background color white, the color code used here is as follows: carbon atoms grey (R/G/B: 128/128/128) and sulphur atoms somewhat darker yellow (223/208/0). Colors for all other atoms were kept as they were by default, i.e., nitrogen blue (57/81/255), oxygen red (255/0/0), hydrogen (in case it is shown) turquoise (70/204/255), and phosphorous orange (255/168/19). Helices are made blue (128/0/255) and Strands, green (0/128/64), with side colors for ribbons made grey (128/128/128). In a normal view, the width of helices and strands are set by default at 3.0 Å and 2.0 Å, respectively, but occasionally this is changed to smaller values to increase the visibility of amino acid side chains that might otherwise be hidden; this is done under 'Prefs' > 'Ribbons'. A structure gets a worm-like shape when 'Helices', 'Sheets' and 'Coils' are given a 'Height' and a 'Width' of 1.0 Å and a spherical (●) 'Shape'.

Other settings may be modified as well in the Toolbar under 'Prefs'. Under 'Rendering' > 'Line width' was changed to 2.0 pixels, and under 'Display' the dot densities for 'VDW' (van der Waals surfaces) and 'Surface' (this is the accessibility surface) were changed to their maximal value (12).

It is possible to make the model continuously rotate to get a clear impression on its overall appearance. Therefore, under 'Prefs' > 'Rock and Roll' define a 'Max Rotation Angle' of 360° and tick off the 'Reverse rotation when max angle has been reached'. Rotation is started by clicking '>' and stopped by 'Esc'. This feature, together with the option to choose a 'Front', 'Top', 'Bottom', 'Left', 'Right' or 'Back' view (under 'Display' > 'Rotate View to see from') allows to obtain a clear 3D picture in all directions of the model on display. Also, rotating a figure is an exquisite way to investigate possible clashes in overlay pictures.

It should be noted that an image needs to be made as large as possible on the laptop screen before saving it as a '*.png' file to optimize the quality. It is useful at that time to remove the link between the Toolbar and the graphic window (under 'Wind' > untick the 'Link Tool and Graphic Windows'), so that the window can be maximized.

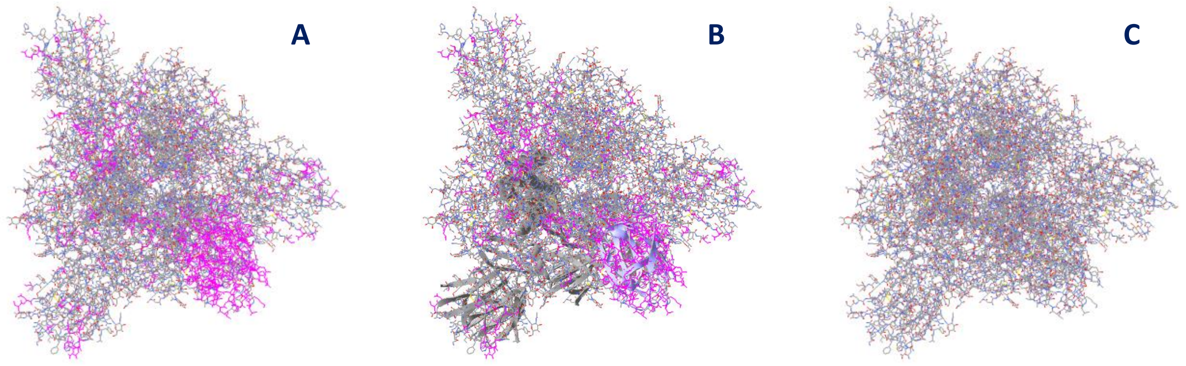

Figure 1. The SARS-CoV-2 spike protein trimer, using coordinates from 6VYB.pdf. (**A**) The model at the moment of uploading. (**B**) A high concentration of purple (modeled) amino acids is found in the RBD of the subunit in open state (chain B), where ribbons are shown colored light blue. (**C**) After changing the colors of backbones and side chains to 'CPK', the complete model shows up in CPK colors.

It occurs regularly that in a pdb file with structural coordinates, some amino acids are missing. The DeepView program then often attempts to make a reconstruction of the complete amino acids including their side chain(s). These modeled residues automatically appear in purple (255/0/255) when the pdb file is uploaded.

A warning shows up and a list of all missing atoms is given as well. An example of a file containing a high number of modeled residues is found in 6VYB.pdb, the SARS-CoV-2 spike glycoprotein ectodomain with two subunits in closed (chains A and C) and one subunit in open state (chain B). When uploading this pdb file, the view is as in **Figure 1A** of this Supplementary Material. The same is shown in **Figure 1B** of this Supplementary Material, where ribbons are added in chain B, colored grey except for the RBD that is colored light blue. A majority of the modeled amino acids belong to the RBD in the open state. After changing the colors for backbone and side chains to 'CPK', all purple colors disappear (**Figure 1C** of this Supplementary Material). 'CPK' coloring, from "Robert Corey, Linus Pauling and Walter Koltun", means that colors are used for the different atoms according to the conventions made in the program (see above). CPK coloring in DeepView for helices and strands makes them all grey.

Peptides and residues that are not available in a model may be localized in DeepView under 'Select' > 'Chain Breaks', after which the bounding amino acids of the missing stretches appear in red in the control panel.

THE SPIKE PROTEIN (S): ONE OF THE MOST IMPORTANT STRUCTURAL MOLECULES OF CORONAVIRUSES

Overall appearance of the SARS-CoV-2 spike glycoprotein trimer

The overall multiple sequence alignment of SARS-CoV-2, SARS-CoV and MERS-CoV spike glycoproteins isolated from human hosts (proteins with accession numbers YP_009724390, P59594 and ASY99778, respectively) is shown in **Figure 2** of this Supplementary Material. Comparison of identities (*), similarities (combination of **:** and **.**) and differences, in percentages, are given in **Table 1** of this Supplementary Material for each of the domains of the spike glycoproteins when the alignments are made with all three virus sequences together, or with only SARS-CoV and SARS-CoV-2.

SARS-CoV-2 and SARS-CoV sequences are far more similar (in % identities plus similarities) with each other than with the sequence of MERS-CoV. Also, similarities within the S2 half of the spike protein are higher than within the S1 half of the molecule for all the different domains. Especially within the NTD, sequence similarities are rather limited, even when comparing only SARS-CoV and SARS-CoV-2 sequences, which indicates that the mutational selective pressure on this domain is fairly low.

Table 1. Analysis of the multiple sequence alignments of the SARS-CoV spike proteins, S1 and S2 regions and their different domains.

		SARS-CoV-2 + SARS-CoV + MERS-CoV			SARS-CoV-2 + SARS-CoV		
	Domain*	Identities, %	Similarities, %	Differences, %	Identities, %	Similarities, %	Differences, %
	Whole protein	*23.4*	*29.0*	*47.6*	*76.0*	*16.1*	*7.9*
	S1 (without SS)	*14.9*	*26.9*	*58.2*	*64.7*	*22.3*	*13.0*
	S2	*34.7*	*31.4*	*33.9*	*90.6*	*8.5*	*0.9*
**S1:**	**NTD**	11.6	25.3	63.1	49.5	31.7	18.8
	**RBD**	14.0	27.5	58.5	73.4	17.7	8.9
	**SD1/SD2**	19.3	28.9	51.8	80.9	14.6	4.5
**S2:**	**SD** (including **FP**)	38.6	32.3	29.1	85.3	12.0	2.7
	**HR1**	53.9	35.5	10.6	88.2	10.5	1.3
	**CH**	50.0	31.3	18.7	100	0	0
	**CD**	21.6	28.4	50.0	87.9	9.1	3.0
	**HR2**	37.5	40.0	22.5	100	0	0
	**TM/CT**	41.7	25.0	33.3	95.8	4.2	0

*The different domains are explained in the legend of **Figure 2** of this Supplementary Material.

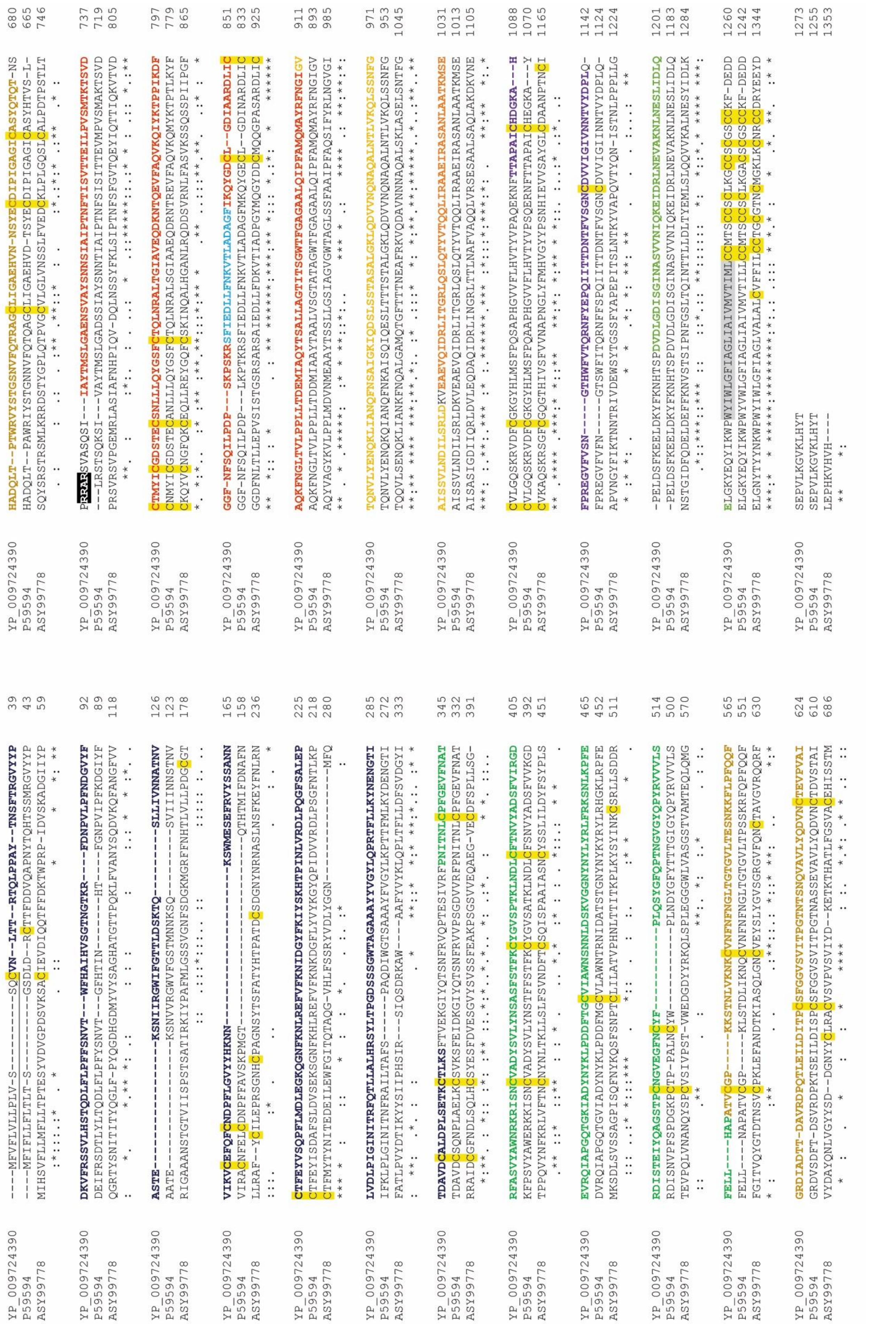

Figure 2 (previous page)

Multiple sequence alignment of SARS-CoV-2, SARS-CoV and MERS-CoV spike glycoproteins. The alignment was done using Clustal omega (Sievers et al., 2011) at EMBL-EBI (https://www.ebi.ac.uk/Tools/msa/clustalo/). In the SARS-CoV-2 sequence, the **SS** (signal peptide) is underlined, the sequence of the **NTD** (N-terminal domain) is colored blue, the **RBD** (receptor-binding domain) green, **SD1/SD2** (structural domains of S1) brown, the **SD** (structural domain of S2) red, with the **FP** (fusion peptide) turquoise, the **HR1** (first heptad repeat) dark yellow, the **CH** (central helix) orange, the **CD** (connector domain) pink, the **HR2** (second heptad repeat) light green, while the furin cleavage sequence is highlighted in black. The **TM** (transmembrane helix), just in front of the **CT** (C-terminal tail), is highlighted in grey. All cysteine residues are highlighted in yellow.

The closed (down) and open (up) conformation of the spike protein

Spike protein subunits regularly alternate between a closed ('down') and open ('up') state. Only in the open configuration is a spike protein subunit able to bind to a receptor in the host. This conformational change is visualized in **Figure 3** of this Supplementary Material for SARS-CoV-2 (**A**) and for MERS-CoV (**B**).

Two models exist for SARS-CoV-2, i.e., 6VXX.pdb with all three subunits in closed state and 6VYB.pdb in which chain B is in open state. Both models are uploaded together in DeepView and found to be in exactly the same orientation. Each model is placed by the program in a different layer and may be worked on individually upon switching between both layers, but they rotate and translate always together (unless their movement is prevented in the 'Layers info' window, by ticking off in the 'mov' column). In **Figure 3A** of this Supplementary Material, only B-chains are shown. The open state is colored red, while the subunit in closed state is colored blue, with the RBD (the peptide P^{330}-P^{521}) being made light blue.

The total length of the SARS-CoV-2 spike protein structure, as it is available in model 6VXX (all subunits in closed state), is about 160 Å, measured from S^{1147}(O) to T^{500} (Cγ2), and about 175 Å in model 6VYB (with subunit B in open state), measured from S^{1147}(O) to Q^{493}(Nε2) in chain B. At the bottom, the spike protein diameter is about 115 Å, from H^{245}(O) till N^{532}(Nδ2), and about 125 Å, from H^{245}(O) till H^{69}(Nδ1), i.e., measured along the height line or along the sideline of the approximately equilateral triangle (as seen in **Figure 1** of this Supplementary Material), respectively. Distances from one atom to another are very easily measured in DeepView by activating the distance button in the Toolbar and picking both atoms one after the other. When measuring the whole length of a molecule, it is sometimes advantageous to check in slab view whether one is indeed looking from one edge of the molecule to the opposite edge (the slab view command reduces the display to a thin slice, 10 Å by default, which is centered at the middle of the molecule; the program then shows only the groups that lie within this slice, and all groups that lie in front of or behind this slice are hidden).

For MERS-CoV as well, two models are found in the PDB database, i.e., 5X5C.pdb with two subunits in closed state (A and B) and one in open state (C), and 5X5F.pdb that has two subunits in open state (A and C) and one in closed state (B). To analyze the difference between the open and closed configuration, chains A were made visible in both models, after uploading them both together in DeepView. Because they were apparently not uploaded in the database in exactly the same orientation, they were first superposed using the 'Magic Fit' option of the program. Only stretches from L^{600} till A^{1206} (i.e., from behind the RBD till the end of the structure) were selected in both models to perform the superposition. Upon then clicking 'Magic Fit', defining 5X5C.pdb to be the reference model, both chains become well superposed, except for the RBDs. The resulting image is shown as **Figure 3B** of this Supplementary Material. Occasionally, the 'Magic Fit' instruction needs to be followed by the 'Improve Fit' function to optimize the quality of the overlay.

Making an overlay in DeepView manually

The automatic 'Magic Fit' function of DeepView cannot always successfully superimpose two models. This was the case when overlaying the RBDs of SARS-CoV and MERS-CoV with that of SARS-CoV-2 (**Figures 5B-C** of the main paper). However, it is even then still possible to superimpose them manually. To superimpose two models, three residues are selected, preferentially in regions that are rather separated in space and where the amino acid sequences are better conserved. In this case, residues N^{360}, D^{398} and Y^{423} (SARS-CoV-2), N^{347}, D^{385} and Y^{410} (SARS-CoV) and N^{406}, D^{444} and Y^{469} (MERS-CoV) were chosen. To superimpose the RBDs from SARS-CoV-2 and MERS (**Figure 5C** in the main paper), both models are uploaded in DeepView and the checkmark of the SARS-CoV-2 model in the 'mov' column of the 'Layer Info' window is removed, to

keep this model fixed. Then, after activating the overlay button in the Toolbar, the Cα atom of N^{406} is picked, followed by the Cα atom of N^{360}, and the same procedure is repeated for the other two residues (D^{444} followed by D^{398}, and Y^{469} followed by Y^{423}). When done correctly, the two models are neatly overlaid, and each layer can eventually be saved separately as a new pdb file.

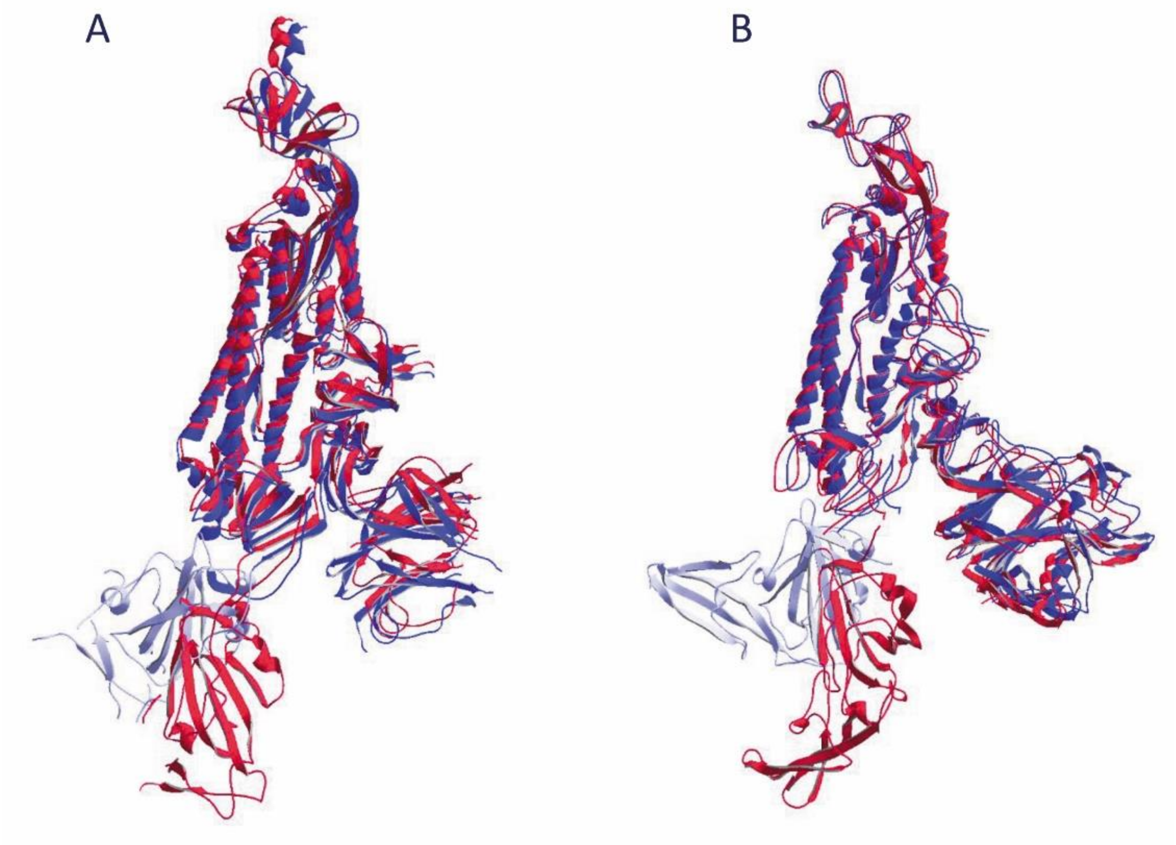

Figure 3. Open and closed states in SARS-CoV-2 (**A**) and MERS-CoV (**B**) spike glycoprotein subunits. In both figures, the ribbons of the subunit in closed configuration are colored blue, with the RBD made light blue, and the subunit in open configuration is colored red. In both models the chain on display is put in roughly the same orientation.

RMS coloring after making an overlay

It can be very instructive to perform an RMS coloring after two models have been superimposed. Two such examples are shown in the main paper (**Figures 9** and **10**). **Figure 9A** shows the RBD of model 6VW1, which is colored orange, while the ACE2 receptor is colored blue. Superposed is the RBD of model 6VXX, of which the ribbons are colored for RMS. RMS coloring for a model means that groups (backbones/side chains/atoms) in that model are colored according to how far they lie from corresponding groups in the other model that is considered the reference model (in this example, 6VW1 is taken as the reference). Regions that superimpose exactly are colored dark blue, with colors farther up the visible spectrum assigned for greater distances from corresponding atoms in the reference model. This is called RMS coloring because DeepView calculates the root-mean-square distances between corresponding backbone atoms to arrive at the color assignment for a group. It was concluded from **Figure 9A** of the main paper that the overall conformation of the RBD in both models does not change dramatically. In the other example (**Figure 10** of the main paper), the overall structural similarities between both ACE domains (**A**) and with ACE2 (**B**) are visualized. The two domains of the ACE enzyme are strikingly similar in structure. Few red peptides are stretches that are missing in the model of the C-terminal domain. But also, the ACE2 structure is very similar with that of an ACE domain. Some of the helices are getting red upon RMS coloring (indicating major structural differences), but solely because they are somewhat displaced in space, though they still run in a very similar way in both molecules.

Spike protein RBD structure in SARS-CoV-2 and MERS-CoV

A more detailed view of the receptor-binding domains (RBDs) of SARS-CoV-2 and MERS-CoV is seen in **Figure 4** of this Supplementary Material. The structures are quite different from each other, especially at the bottom right part of the domain. Both RBDs contain four disulfide bonds, but one of them uses an alternative cysteine residue in the MERS-CoV RBD. These differences in RBD structure and amino acid sequences explain why SARS-CoV-2 (as well as SARS-CoV) binds to ACE2 as receptor molecule in the host, while MERS-CoV uses DPP4.

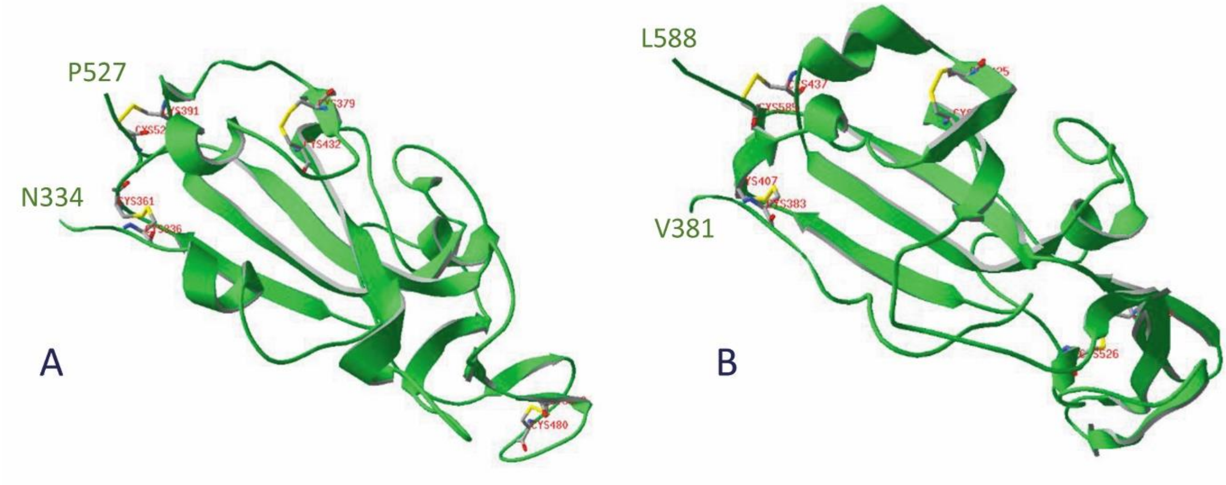

Figure 4. The SARS-CoV-2 receptor-binding domain from 6VW1.pdb (**A**) and the MERS-CoV RBD from 4KR0.pdb (**B**), showing the four disulfide bonds in this part of the spike proteins. Their side chains are added to the pictures, labeled in red in DeepView. The position of the first and the last residue of the RBDs are indicated manually. Both models are presented in roughly the same orientation.

Cysteine residues in the SARS-CoV spike protein

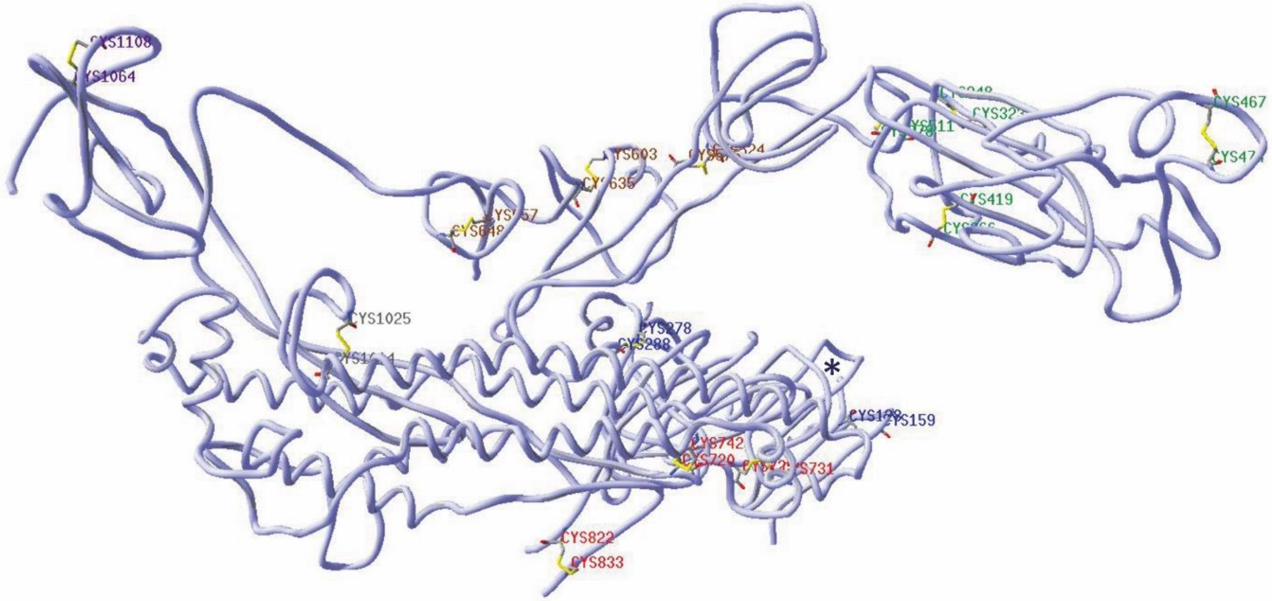

Figure 5. The SARS-CoV spike glycoprotein chain B from 5XLR.pdf, showing the position of the 14 disulfide bonds. The cysteines are labeled in different colors depending on the domain to which they belong, i.e., those in the NTD are labeled blue (C^{128}-C^{159} and C^{278}-C^{288}), those in the RBD green (C^{323}-C^{348}, C^{366}-C^{419}, C^{378}-C^{511} and C^{467}-C^{474}), those in the SD1/SD2 region are brown (C^{524}-C^{576}, C^{603}-C^{635} and C^{648}-C^{657}), those in the SD of the S2 half of the molecule red (C^{720}-C^{742}, C^{725}-C^{731} and C^{822}-C^{833}), the one immediately after the CH black (C^{1014}-C^{1025}), and the one of the CD purple (C^{1064}-C^{1108}). The single cysteine residue C^{133} is located in the vicinity of the star but missing in the structure. The whole chain is given a worm-like shape.

Figure 5 of this Supplementary Material shows the 14 disulfide bonds in the ectodomain of SARS-CoV, using model 5XLR.pdb (in the SARS-CoV-2 model 6VXX.pdb, two of them are missing in the structure). Cysteine residues in the short endodomain (which is not shown in the structure) are post-translationally modified with very hydrophobic palmitoyl chains: CH_3-$(CH_2)_{14}$-[C=O]–S-protein.

Spike glycoprotein binding to its receptor

Figure 6 of this Supplementary Material shows a picture of the contact region between the ACE2 receptor (in green) and the viral RBD (in pink). The idea behind this figure was to compare the experimentally determined structure (available in 6VW1.pdb) with a hypothetical structure that was produced through modelling based on the already known SARS-CoV structure. The modelled structure was presented in a very early publication, before real structural data were available for SARS-CoV-2 (Wan et al., 2020). When comparing **Figure 6** of this Supplementary Material with Figure 1D from Wan et al.'s paper (in which the same amino acid side chains are shown, and the same coloring is used), we conclude that, although both figures are very similar, a number of amino acid side chains in the predicted model do not point in the same direction as in the experimental model. This is again an example confirming that a model always remains purely hypothetical. One can only be precise after having obtained real structural data from experiments.

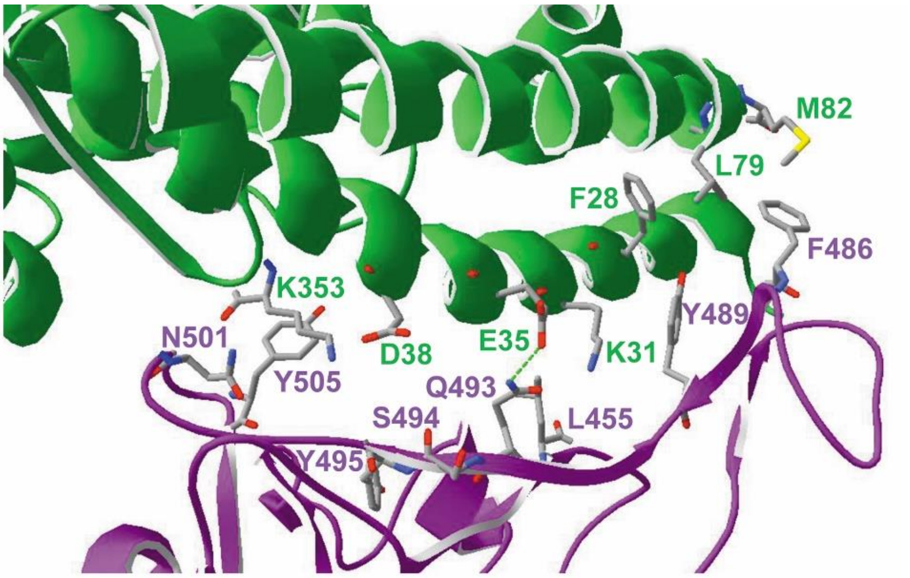

Figure 6. The interface between the ACE2 receptor (green) and the viral RBD (pink), here presented in the same way as in the publication by Wan et al. (2020). This figure is made showing the same side chains and the same colors as in Wan et al.'s paper but using coordinates from 6VW1.pdb. Labels were added manually. One hydrogen bond between an ACE2 receptor and a viral RBD residue is seen as a green dashed line.

To clearly display the amino acid side chains, the option 'Render in solid 3D' is selected under 'Display' in the Toolbar (in this and all following images).

Flexibility in the spike glycoprotein

Figure 7 of this Supplementary Material shows the SARS-CoV-2 spike protein trimer colored for B-factor (see section "Flexibility in the Spike Glycoprotein" in the main paper for an explanation). The region where we find residues having the highest B-factors is the receptor-binding domain, i.e., from residue P^{330} till P^{521} (from light green up to orange). The residue with the highest B-factor in each subunit is Y^{489}.

The three green areas at the corners of the triangle in the bottom-to-top view of **Figure 7B** of this Supplementary Material, which point to somewhat lesser flexibility, are the N-terminal domains of the subunits. The central portion of the spike protein trimer (the S2 part) shows up dark blue, indicating that this part of the trimer would by far be more rigid. The C-terminal end of the spike protein monomers (as far as their structure is available in the model), from the connector domain on, is lighter blue, indicating that the spike

protein might also be moving as a rigid body at the surface of the virion. In **Figure 7C-D** of this Supplementary Material, a picture is shown in which only the RBD backbone and side chains are visible (from residue P^{330} till P^{521}), colored for B-factor, while the other parts of the molecule are shown as ribbons (without backbone and side chains). These ribbons are colored red (chain A), blue (chain B) and green (chain C). The residue with the highest B-factor (Tyr^{489}) is labeled in the DeepView program. It should be noted that a label in DeepView is always placed close to the alpha-carbon atom (Cα) of the residue under consideration, which makes identification very easy.

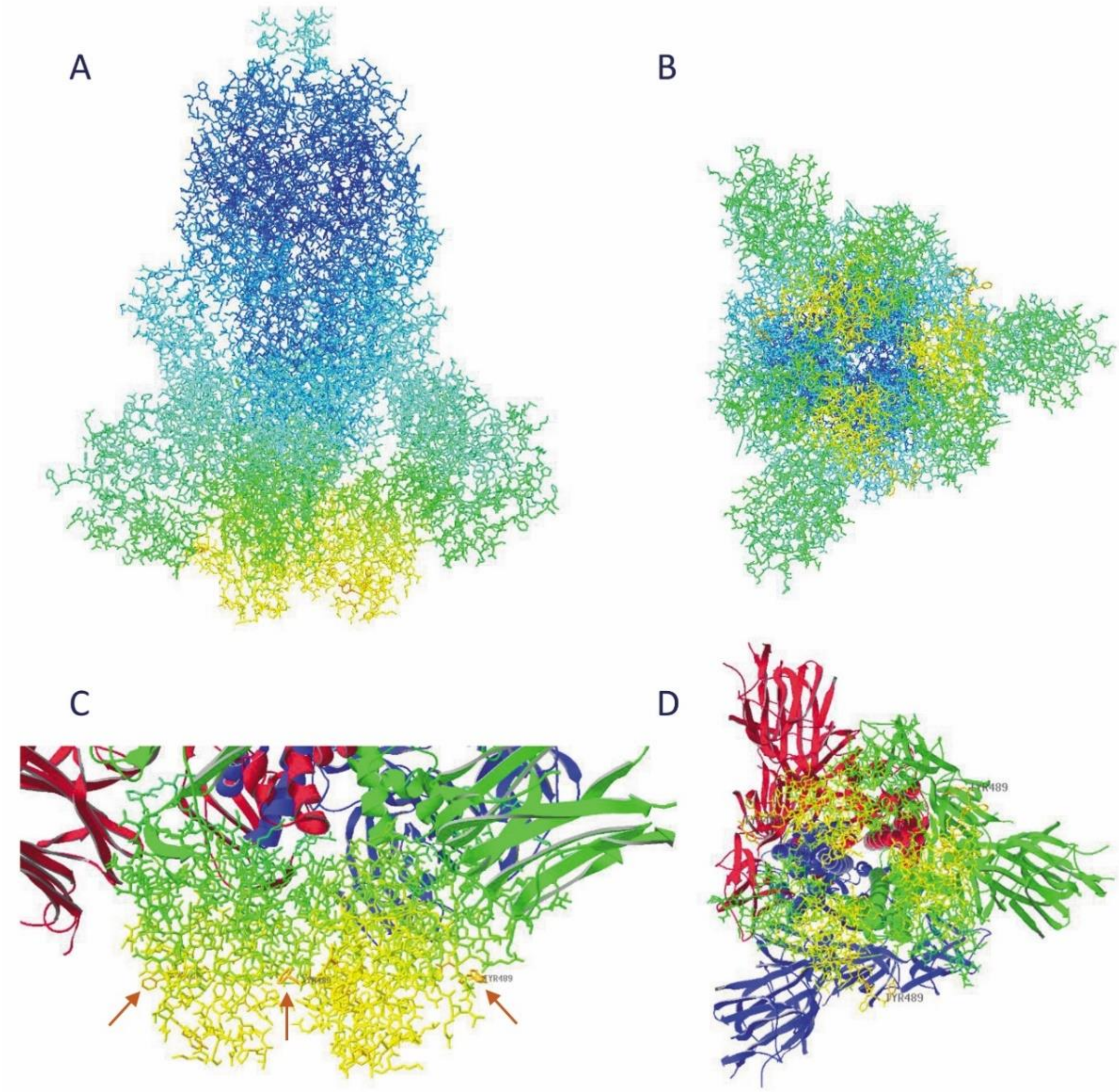

Figure 7. The SARS-CoV-2 spike protein trimer (model 6VXX.pdb, structure determined at 2.8 Å resolution) is colored for B-factor and shown as a side view (**A**) and in bottom-to-top view with the virion behind the protein (**B**). (**C**) A close-up of the lower part of the SARS-CoV-2 trimer that faces the ACE2 receptor (model 6VXX.pdb) as a side view, showing the three very flexible (high B-factors) receptor-binding domains. The Y^{489} residues are labeled and their position indicated with arrows. (**D**) A bottom-to-top view (with the virion at the back) of the spike protein trimer. The RBD backbone and side chains are seen (from residue P^{330} till P^{521}), colored for B-factor, while the other parts of the molecule are shown as ribbons (without backbone and side chains). A-chain red, B-chain blue, C-chain green.

Potential role of the glycocalyx at the onset of a coronavirus infection

As mentioned in the main paper, the SARS-CoV-2 RBD surface in particular is remarkably electropositive. To display the molecular surface with electrostatic potential in DeepView, all groups are selected that should not be included in the surface. The RBD in the model (from T^{333} till P^{527}) is initially shown as ribbons colored for CPK, without backbone and side chains. Ticking the 'Invert Selection' then deselects the whole RBD. Next, in 'Tools' > 'Surface' > 'Compute', the surface of that part of the model that was deselected is calculated and displayed. The following settings should be used in 'Prefs' > 'Surfaces' > tick on 'Electrostatic Potential' and 'Ignore Selected Residues'; 'Plain Lines' are used in the 'General Appearance'. Colors added by the program are from red (negative surface), over white, to blue (positive surface). **Figure 8A** of this Supplementary Material shows that the surface of the RBD has an overall positive charge, which might explain the observed binding of heparan sulfate. **Figure 8B** of this Supplementary Material shows the backbone with all side chains of the RBD, colored according to their properties (basic, acidic, polar non-charged, or hydrophobic), and showing the position of all positively and negatively charged residues that contribute to the surface electrostatic potential. For comparison, the surface of the SARS-CoV spike protein RBD is shown in the inset (**Figure 8A** of this Supplementary Material).

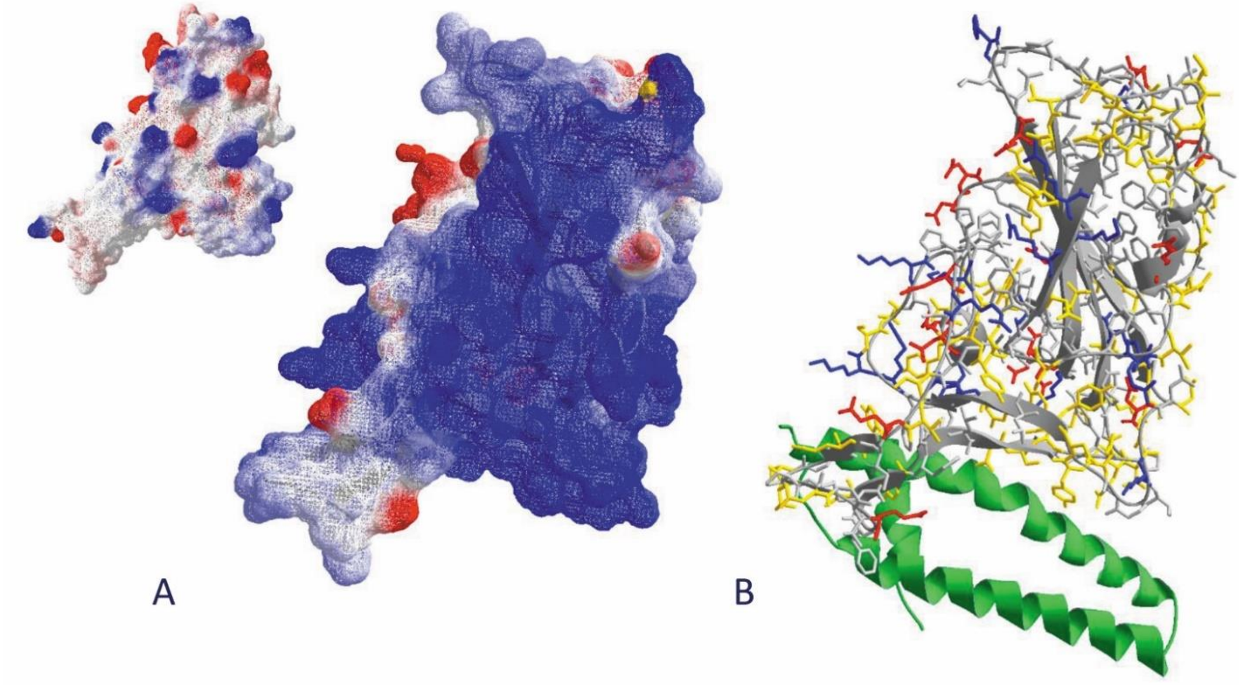

Figure 8. (**A**) The SARS-CoV-2 spike protein RBD (model 6W41.pdb) is displayed as a molecular surface and colored according to its electrostatic potential. The inset (left) represents the SARS-CoV spike RBD (model 6WAR.pdb) in the same orientation, for comparison. (**B**) The same SARS-CoV-2 model in exactly the same orientation without the surface, with backbone and side chains colored according to the amino acid type (basic, acidic, polar non-charged and hydrophobic residues are blue, red, yellow and grey, respectively). Ribbons are added to the RBD in CPK colors (grey). The first part of ACE2 receptor with the first two long helices (S^{19} till G^{104}) is added as green ribbons.

ACE cannot act as a receptor for SARS-CoV-2

ACE2 (angiotensin-converting enzyme-2, EC 3.4.17.23) and ACE (angiotensin-I-converting enzyme, EC 3.4.15.1) are homologous proteins that both exist in different organs in humans. Both ACE2 and ACE are single-pass type-I membrane-bound enzymes with a short C-terminal endodomain. Human amino acid sequences may be found in the UniProtKB database with accession numbers Q9BYF1 for ACE2, and P12821 for ACE.

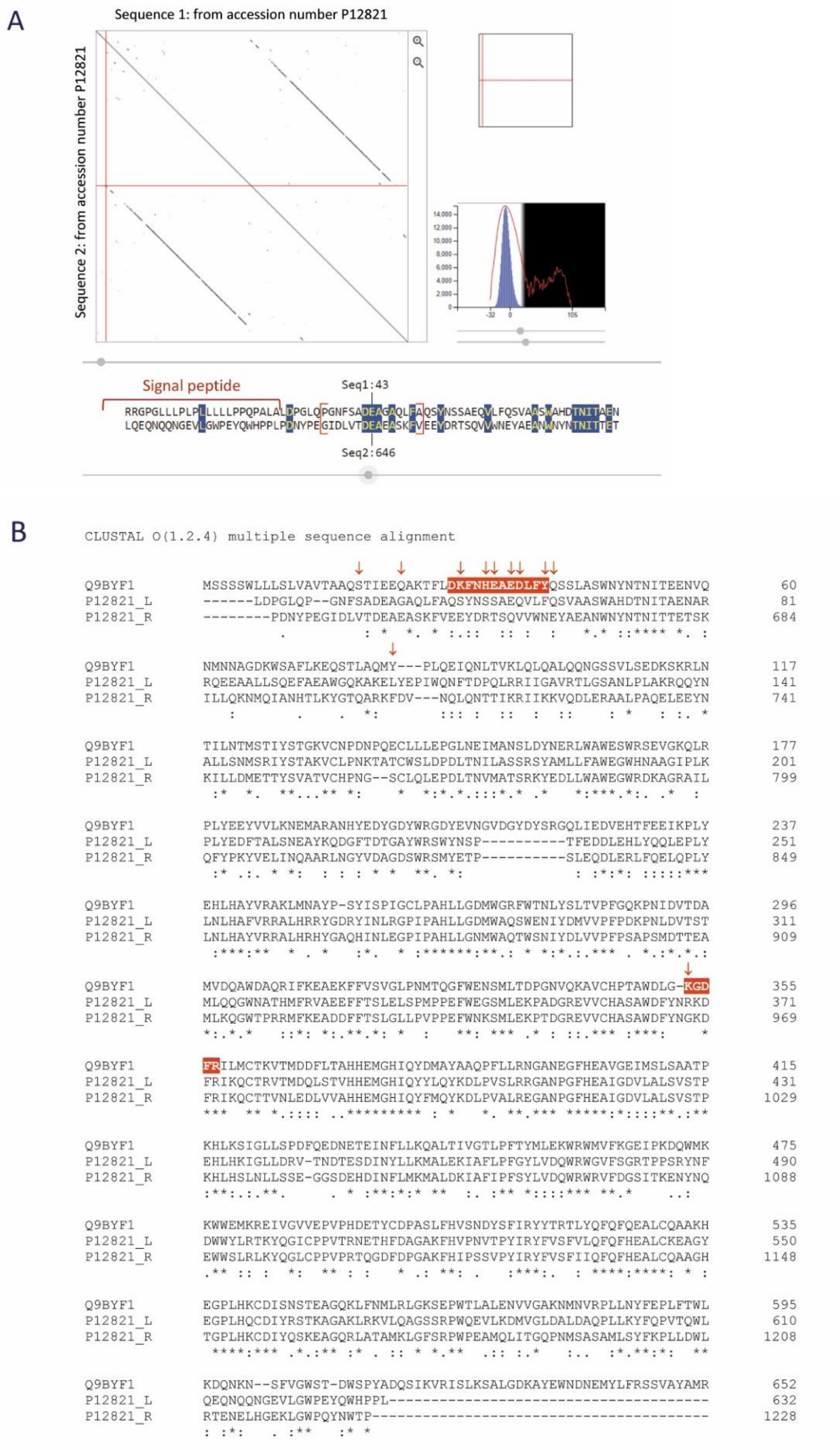

Figure 9. (**A**) The use of Dotlet-JS to identify internal duplications in a sequence, here the amino acid sequence of ACE (accession number P12821). (**B**) Based on the Dotlet result, the ACE protein sequence was divided in two halves (indicated as left, L, and right, R, in the accession number) and, together with the ACE2 sequence (accession number Q9BYF1), aligned using Clustal omega. Residues that are in close proximity to the RBD of the SARS-CoV-2 spike protein are indicated with a red arrow. Residues that are indicated in the NCBI protein database to interact with the SARS-CoV spike glycoprotein are highlighted in red.

The ACE molecule is built from two very similar domains, each of them displaying enzyme activity. The duplication within ACE can easily be analyzed using the program Dotlet-JS (https://dotlet.vital-it.ch/) (Junier and Pagni, 2000), using the protein sequence with accession number P12821. Dotlet is a program that compares a sequence with itself and displays internal duplications as lines additionally to the diagonal line. Corresponding peptides with similar sequences may easily be identified and aligned using Clustal omega (Sievers et al., 2011) or a similar multiple sequence analysis tool, as illustrated in **Figure 9** of this Supplementary Material.

From the multiple sequence alignment, it is obvious that the N- and the C-terminal half of the ACE protein molecule are very similar. Moreover, they also align nicely with the ACE2 amino acid sequence. Upon aligning all three sequences, identities and similarities were calculated to be 29.0% and 31.1%, respectively, and the differences, 39.9%. Only a limited number of gaps were needed to optimize the alignment.

In **Figure 7** of the main paper, we looked in detail to the interface between the SARS-CoV-2 RBD and its known receptor, ACE2. Residues were selected that are located within a distance of 3.2 Å from the opposing chain. Most of the selected receptor residues were found to belong to one of the long ACE2 α-helices (i.e., residues S^{19}, Q^{24}, K^{31}, H^{34}, E^{35}, E^{37}, D^{38}, Y^{41}, Q^{42}), plus Y^{83} and K^{353}. These residues are indicated in the alignment with red arrows. Since they do not belong to a region where the amino acid sequence is well conserved, it is not surprising that ACE does not act as a receptor molecule for SARS-CoV-2.

The overall structural similarities between both ACE domains, and with ACE2 are visualized in **Figure 10** of the main paper.

The much-debated lucrative spike protein mutant $D^{614}G$

As early as February 2020, a point mutation ($D^{614}G$) in the SARS-CoV-2 spike protein emerged, which quickly spread around the world and has by now completely supplanted the original virus. This requires urgent addressing as the mutation seems to make the virus easier transmissible and more deadly. Moreover, it might reduce the efficacy of drugs and vaccination strategies already in the pipeline using the original virus or its components.

Figure 10 of this Supplementary Material gives an impression of the surroundings of residue D^{614}. This residue is located at an internal interface between the individual subunits of the spike protein trimer. Its position in the SARS-CoV-2 spike subunit is indicated in **Figure 4** of the main paper. The distance between the Cα-atoms of D^{614} and the two residues flanking the peptide in which proteolysis S1/S2 occurs is roughly 30 Å. Residue D^{614} is readily open to solvent, which is reflected by its wide accessibility surface (**Figure 10A** of this Supplementary Material). The orientation of this residue's side chain is quite different when a subunit changes from a closed to an open conformation, as well as its relationship with its neighbors. Model 6VSB.pdb that was used to make the figures, has one subunit in open state (chain A) and the other two in closed configuration. The neighbors at 5 Å of A-chain residue D^{614} are F^{592}, G^{593}, Y^{612}, Q^{613}, V^{615}, N^{616} and B-chain residues T^{859} and V^{860}, and a hydrogen bond is formed between the Oδ2 side chain oxygen of D^{614} and the Oγ1 side chain oxygen of T^{859} (green arrow in **Figure 10B** of this Supplementary Material). The neighbors of B-chain residue D^{614} are F^{592}, Y^{612}, Q^{613}, V^{615}, N^{616} and C-chain residues K^{584}, T^{859} and V^{860}. Finally, the neighbors of C-chain residue D^{614} are again F^{592}, Y^{612}, Q^{613}, V^{615}, N^{616} and A-chain residue T^{859}. In contrast, neither in B- nor in C-chain the side chains of residue D^{614} are implicated in hydrogen bond interactions. Moreover, distances between Oγ1 side chain oxygens of T^{859} with the two side chain oxygen atoms of D^{614} differ in the three subunits (as measured in DeepView: from 4.46 and 2.75 Å in chain A, and 6.02 and 4.39 Å in chain B, to 4.65 and 3.38 Å in chain C). From these figures one may conclude that this residue D^{614} indeed occupies a central position with respect to the conformational changes from a closed to an open state.

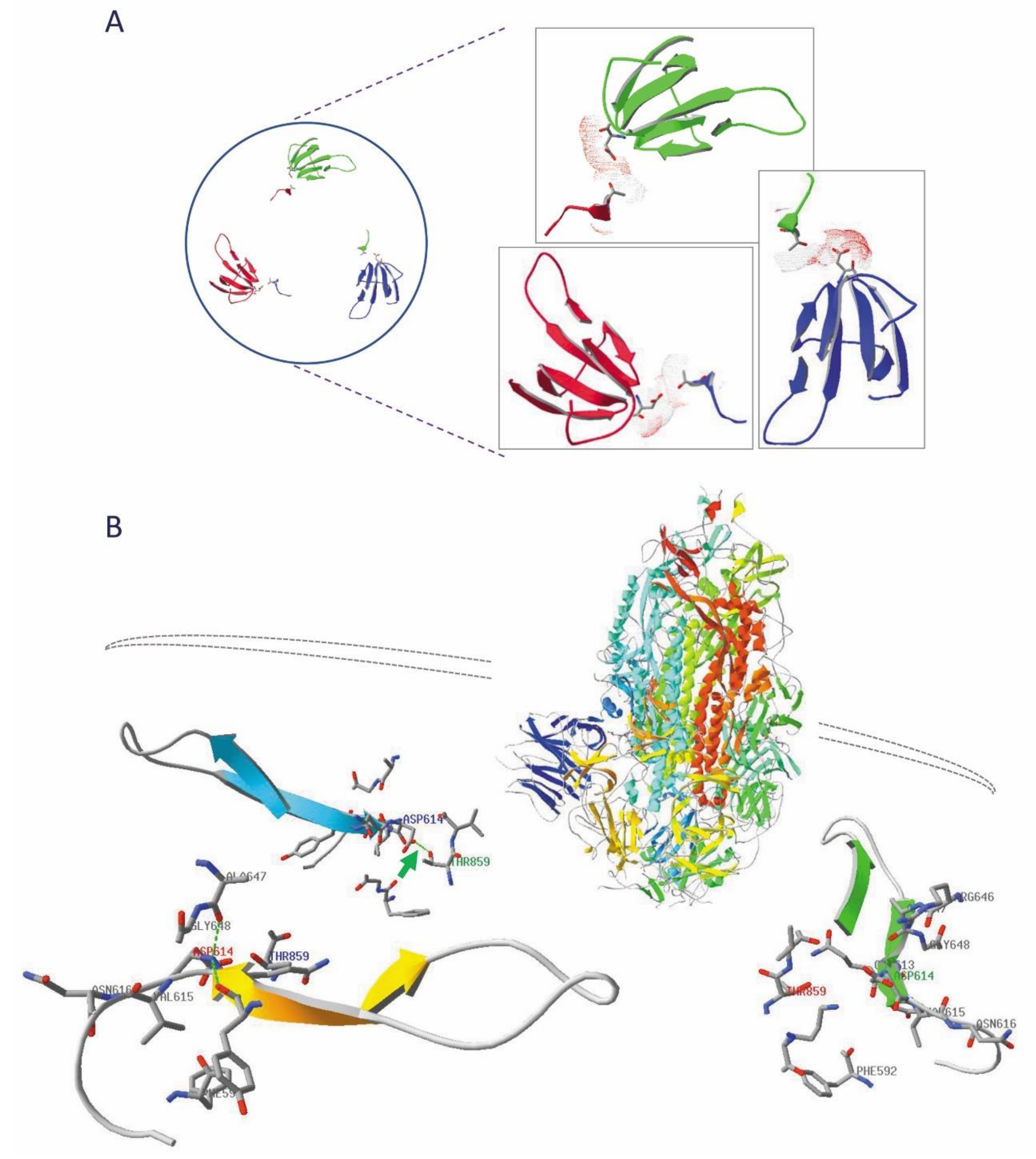

Figure 10. (**A**) Environment of residue D^{614} that was spontaneously mutated to G^{614}. This residue is about 30 Å away from the peptide in which the cleavage S1/S2 occurs (not in the picture). Images were generated from model 6VSB.pdb. Figure on top (encircled) shows a horizontal cut through the spike protein trimer with, in all three subunits, stretches from I^{598} till A^{672} and from Q^{853} till T^{859}, in ribbons and colored red, blue and green for chains A, B and C, respectively. In the enlargements, the side chains of residues D^{614} and T^{859} are shown as well, with their accessibility surfaces. (**B**) A full spike protein trimer is displayed (from model 6VSB.pdb, shown as ribbons, colored for secondary structure succession). Without turning the model, a slice of the structure, localized at the level of the dotted lines, is enlarged by selecting all three D^{614} residues, to which were added residues at a distance of 5 Å. Beta-strands are still colored for secondary structure succession, and labels of residues D^{614} and T^{859} are colored blue, green and red when they belong to chains A, B and C, respectively. Hydrogen bonds are shown as green dotted lines.

Soluble mutated ACE2 analogues as a decoy receptor

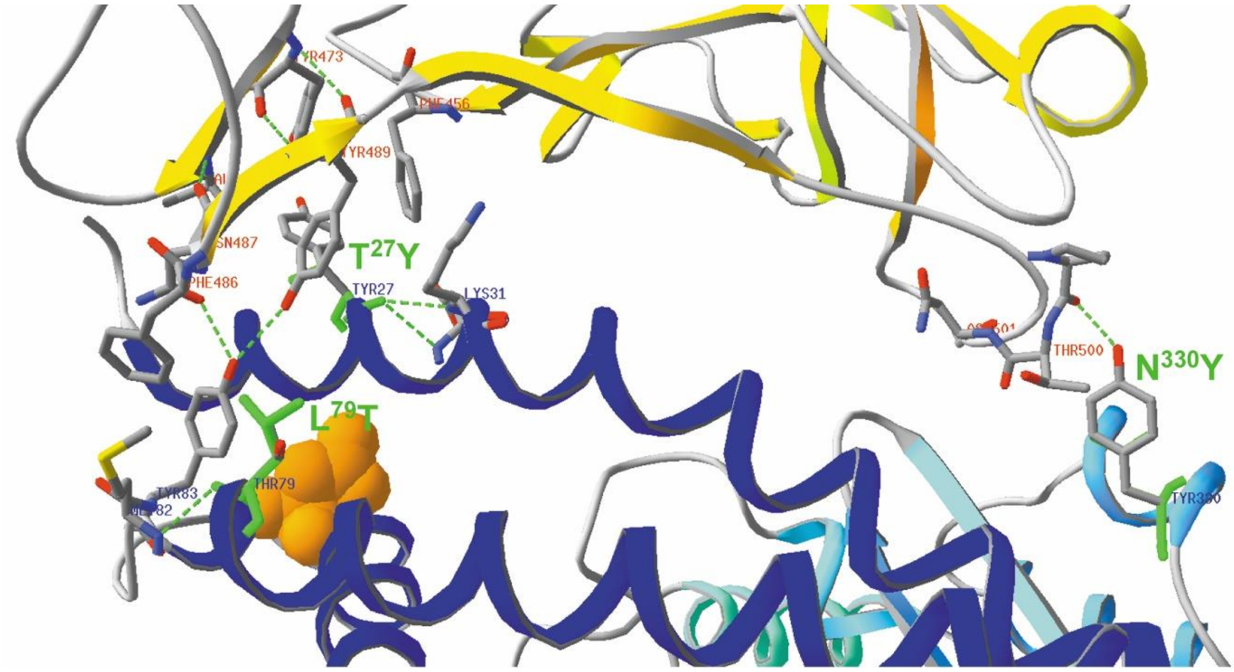

Figure 11. Three residues in ACE2 (in model 6VW1.pdb, chain A) were virtually mutated as follows, after activating the mutation button in the Toolbar: $T^{27}Y$, $L^{79}T$ and $N^{330}Y$. Initially, neighbors at 10 Å around each of the original residues were selected before doing the mutations. The new side chains were found to fit in the ACE2 structure without problems. Then the three mutated side chains were displayed with ribbons of chains A and E (E is the SARS-CoV-2 RBD), which are colored for secondary structure succession (the ribbons are reduced to a width of 1 Å), thereby coloring the ACE2 receptor blue and the SARS-CoV-2 RBD yellowish. Some of the amino acid side chains that are within a distance of 5 Å from the three mutations are finally added to the image and labeled (in blue and red for chain A and E, respectively). The original side chains (T^{27}, L^{79} and N^{330}) are added to the figure and shown in green for comparison. Hydrogen bonds involving the three mutations are shown as green dotted lines. It may be noticed that the DeepView program also automatically changed the names of the three mutated residues. The ACE2 residue N^{90} that carries a N-glycan is shown with its van der Waals surface, colored orange (this is done by giving an orange color to both the backbone and side chain of residue N^{90}). The three mutated residues are also manually labeled in green.

An interesting idea to combat Covid-19 was launched making use of high affinity soluble ACE2 analogs. **Figure 11** of this Supplementary Material gives an impression of a triple ACE2 mutant $T^{27}Y$, $L^{79}T$, $N^{330}Y$. The ACE2 residue T^{27} lies at the interface with the SARS-CoV-2 RBD. A mutation to tyrosine does not disturb the contact region with the RBD, but brings three viral RBD aromatic residues F^{456}, Y^{473} and Y^{489} (plus a hydrophobic residue A^{475}) in its vicinity, which might increase the binding strength because of additional stacking interactions. Residue N^{330} lies at the interface with RBD as well. Its mutation into a tyrosine introduces a new hydrogen bond with the backbone oxygen of RBD's residue P^{499}, which may also contribute to the binding strength. The third mutation $L^{79}T$ is somewhat deeper in the ACE2 molecule, further away from the interface. The position of the N-glycan on residue N^{90} of ACE2 is indicated in **Figure 11** of this Supplementary Material by showing the van der Waals surface of the asparagine (in orange).

Binding of antibodies to the spike glycoprotein

Most of the antibodies that were analyzed in literature compete for binding to the ACE2 receptor, as can be seen from **Figure 12** of this Supplementary Material. **Figure 12A** shows extensive and obvious clashes with the ACE2 receptor when an antibody (the Fab fragment from model 6XC4.pdf in this example) binds to the RBD. The same is true for Fab fragments in models 6XC2, 7BZ5 and 7C01, which all bind to the same region of the RBD. Several studies were published on antibody CR3022, which was isolated from a SARS-CoV

patient. It was found to be directed against the RBD and proven to be neutralizing for SARS-CoV but not for SARS-CoV-2, though it is able to bind to its RBD (see section “Binding of Antibodies to the Spike Glycoprotein” in the main paper). Binding of this Fab fragment to the spike protein RBD is presented in **Figure 12B** of this Supplementary Material, showing only minor clashes with the ACE2 receptor. This is also true for the Fab fragment in model 7BWJ with some, though much less prominent, clashes are observed (see **Figure 12C-D** of this Supplementary Material).

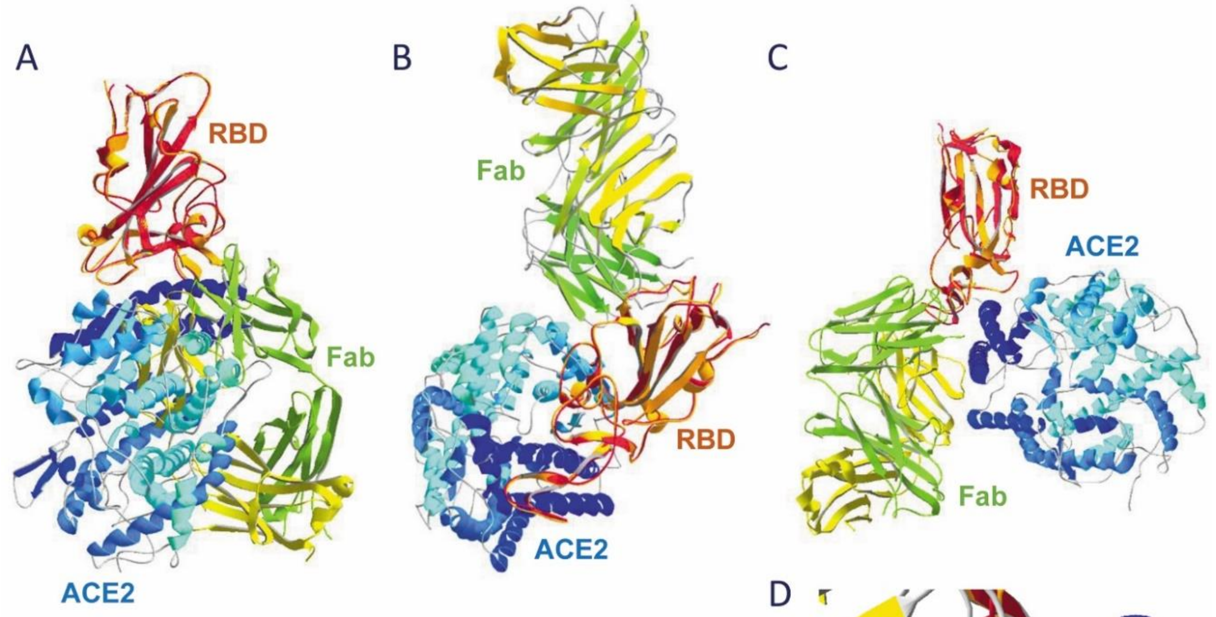

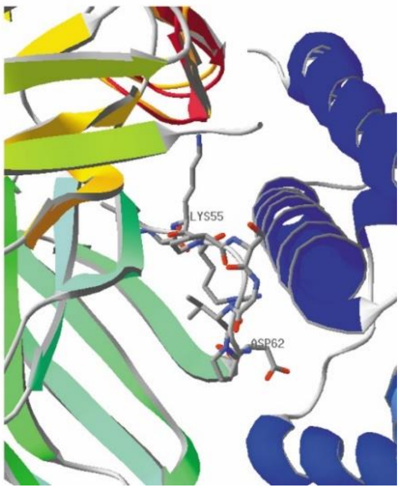

Figure 12. Analysis of overlap in binding between SARS-CoV-2 RBD and classical antibody Fab domains. After making the overlay using the RBD from model 6VW1 (colored orange) and the RBDs from the models with the antibody (colored red), the ACE2 receptor (from model 6VW1.pdb) is colored for secondary structure succession, while the Fab H- and L-chains are colored green and yellow, respectively. (**A**) Figure made using model 6XC4.pdb (Fab from mAb CC12.3 from a Covid-19 patient), showing extensive clashes between the receptor and the Fab domains. (**B**) Figure using the Fab from model 6Z2M.pdb (Fab from mAb CR3022 from a convalescent SARS-CoV patient). (**C**) Figure using model 7BWJ.pdb (Fab from mAb P2B-2F6 from a Covid-19 patient). (**D**) Enlargement of the central part of figure C, showing that there are clashes between residues in the loop V^{53}SKRPSGVPD^{62} of the antibody light chain with the receptor molecule.

Highlighting clashes between two structures in DeepView

After superimposing two structures in DeepView, it is often the intention to analyze whether some of the molecules would give clashes, preventing them from being present in the same space at the same time. In **Figure 13** of this Supplementary Material, it was the aim to find out whether an antibody Fab fragment that binds to the SARS-CoV-2 RBD would prevent binding of that RBD to an ACE2 receptor molecule by competing for the same molecular surface. Therefore, the RBD in model 6VW1.pdb (which is a structure of the RBD bound to the ACE2 receptor) was superimposed with the RBD in the models 6XC4.pdb, 6Z2M.pdb and 7BWJ.pdb (as shown in **Figure 13** of this Supplementary Material). Now, we want to get a detailed view on possible clashes that would occur between the Fab fragments and the ACE2 molecule. This is realized as follows (using model 6XC4.pdb as an example). Both models (6VW1 and 6XC4) are uploaded in DeepView and were superimposed using the RBDs. At this moment, they are still in two different layers in the program.

Then, all chains that we want to look at are selected in both models, i.e., chains E (the RBD) and A (the ACE2 receptor) in model 6VW1, and chains X and Y (the H and L chains of the Fab fragment) in model 6XC4. Now, we will bring these four chains in one single layer. Upon clicking 'Edit' in the Toolbar > 'Create Merged Layer from Selection', a third layer is now created that is named '_merge_' by DeepView and that contains all selected chains (A, E, X and Y). When acting on this new layer, upon ticking 'Select' > 'Residues Making Clashes', all residues that are clashing (but only these) show up. Moreover, they appear with pink dashed lines. **Figure 13A** of this Supplementary Material shows that, indeed, a lot of clashes appear, many of them involving the contact surface between the RBD and the ACE2 receptor. This explains why this antibody has exquisite neutralizing capacity. Appreciably less clashes are seen using model 6Z2M.pdb (**Figure 13B** of this Supplementary Material), and only 6 residues of the ACE2 receptor, with 6 residues (L-chain) and 1 residue (H-chain) of the antibody Fab fragment are making clashes when using model 7BWJ.pdb (**Figure 13C** of this Supplementary Material). Of course, in the latter case, where only very small numbers of clashes are detected, all conclusions need to be considered with due care because the overlay that was made to produce this combined structure might not have been fully accurate.

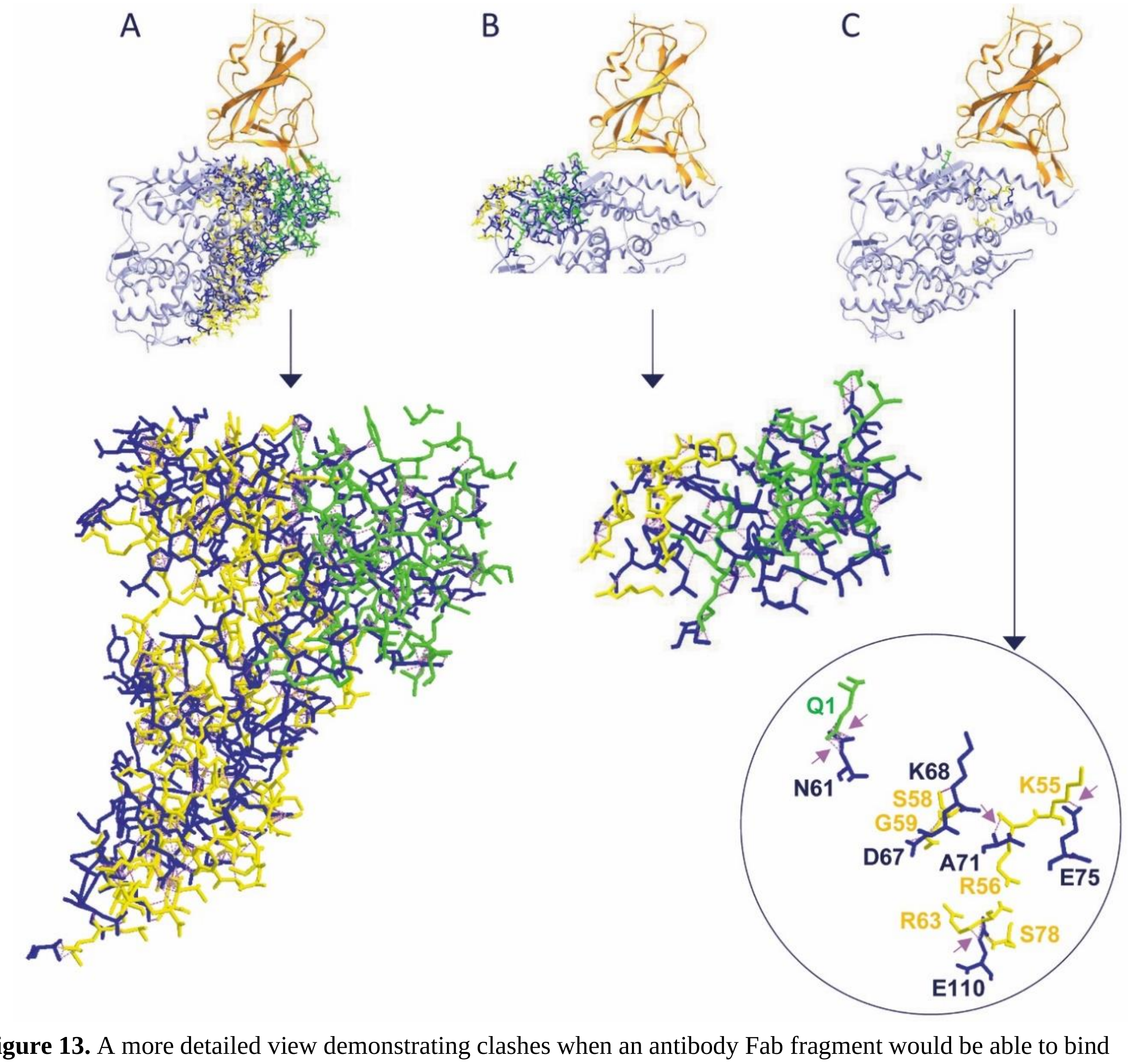

Figure 13. A more detailed view demonstrating clashes when an antibody Fab fragment would be able to bind together with the ACE2 receptor to the SARS-CoV-2 spike protein RBD. On top of all three figures, the RBD is shown as ribbons, colored orange, with underneath the ACE2 receptor as ribbons colored light blue (the width of all helices is reduced to 1 Å). The residues that give a clash between the ACE2 receptor and the Fab fragment are shown with backbone and side chains, which are colored blue, green and yellow for residues that belong to the ACE2 receptor or the Fab heavy and light chain, respectively. Below: an enlargement, in the same orientation, of all clashes (shown by DeepView as pink dashed lines, some are also indicated manually with a pink arrow) and using the same color code for backbones and side chains as above. The following coordinates were used for the Fab fragments: (**A**) model 6XC4.pdb; (**B**) model 6Z2M.pdb; (**C**) model 7BWJ.pdb.

Structural differences in binding of neutralizing antibodies that recognize either the RBD or the NTD

In **Figure 14** of this Supplementary Material, the overall structure of a SARS-CoV-2 spike trimer in complex with Fab fragments binding to the RBD is compared with the structure of the spike protein in complex with Fab fragments that bind to the NTD. The first Fab (**Figure 14A-B** of this Supplementary Material) is a fragment from a neutralizing monoclonal antibody (mAb S309) that was isolated from memory B-cells of a SARS-CoV convalescent patient (Pinto et al., 2020). The second Fab (**Figure 14C-D** of this Supplementary Material) is a fragment from a neutralizing monoclonal antibody (mAb 4A8) that was isolated from a convalescent Covid-19 patient (Chi et al., 2020).

In **Figure 14A-B** of this Supplementary Material, the structure is presented of three antibody Fab fragments bound to the three RBD domains of a spike protein trimer (figure made using model 6WPS.pdb, in which all spike protein subunits are in a closed state). **Figure 14C-E** of this Supplementary Material shows a structure of the SARS-CoV-2 spike trimer in complex with three Fab fragments, each of them obviously binding to a different NTD. In this model (7C2L.pdb), the RBD of chain A (colored orange in **Figure 14** of this Supplementary Material) is in the open conformation, while RBDs of chains B and C are in a closed conformation.

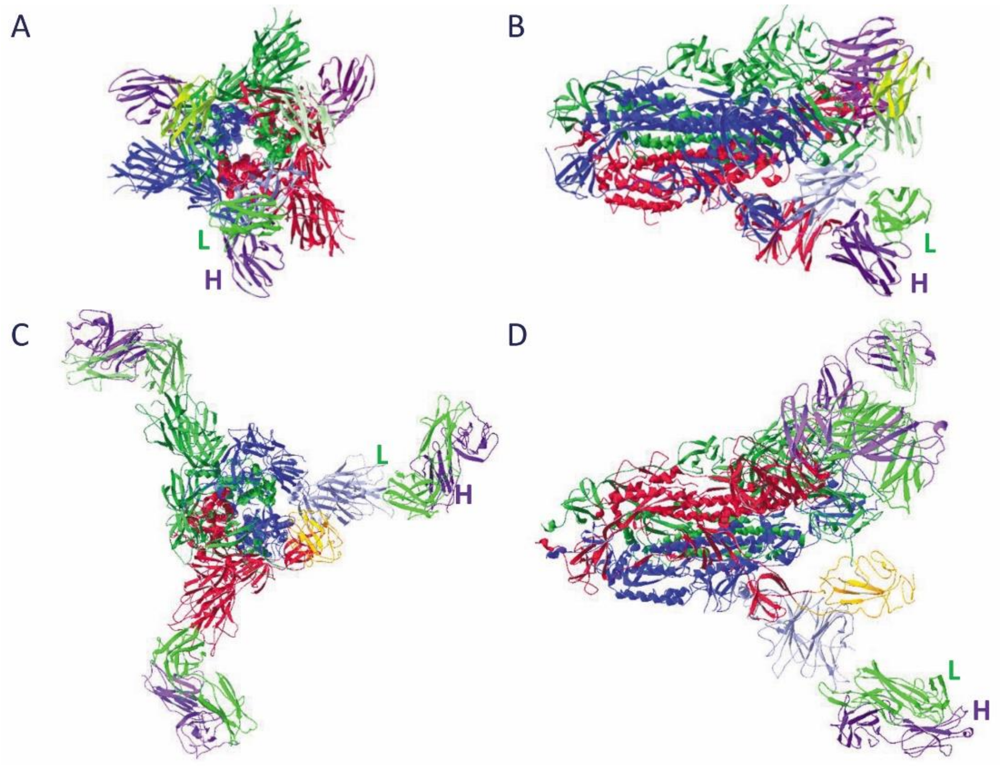

Figure 14. A neutralizing antibody Fab fragment (mAb S309) binding to the RBD of SARS-CoV-2 spike protein. (**A**) A full SARS-CoV-2 spike protein trimer (in closed state) with three Fab molecules bound to the RBD domains, in bottom-to-top view with the virion at the back. (**B**) Same structure, but as a side view (with the virion to the left). Both figures were made using model 6WPS.pdb. The three spike protein subunits are colored red, blue and dark green (chains A, B and E, respectively), with the RBD of the B-chain colored light blue. Antibody heavy and light chains are colored purple and light green, respectively. Both chains of the antibody binding to the RBD of subunit B are manually labeled.
A neutralizing antibody Fab fragment (mAb 4A8) binding to the NTD of SARS-CoV-2 spike protein. (**C**) A full SARS-CoV-2 spike protein structure is displayed, generated from model 7C2L.pdb, in bottom-to-top view (with the virion at the back). (**D**) The same as a side view (with the virion to the left). All chains are shown as ribbons. The spike protein subunit chains A, B and C are colored red, blue and green, respectively, with the RBD of chain A in orange, and the NTD of chain B in light blue. Antibody heavy and light chains are colored purple and light green, respectively. Both chains of the antibody binding to the NTD of subunit B are manually labeled. (**E**) In the enlarged image (next page) the residues that limit the NTD (from Q^{14} till S^{305}) and the RBD (from P^{330} till P^{527}) are added manually. The NTD belongs to chain B, while the RBD is part of chain A.

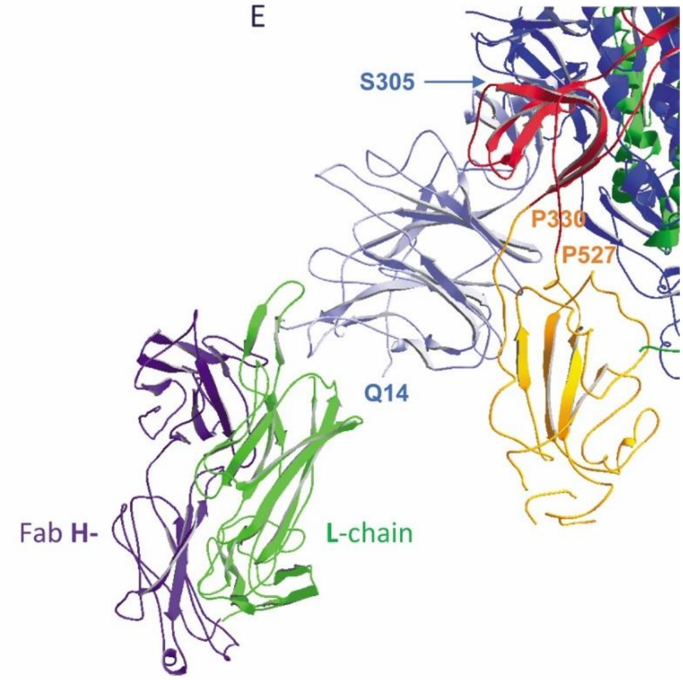

Figure 14. (**E**) *See legend on the previous page*.

Figure 15 of this Supplementary Material shows a detailed view of the interface between the spike protein's NTD and the Fab fragment. All residues within a distance of 3.5 Å are shown with their side chains. Three hydrogen bonds are formed between residues from the spike protein and the Fab (indicated as green dotted lines). It is striking that all paratopes of the Fab that interact with the epitopes on the NTD belong to the heavy chain (colored in green).

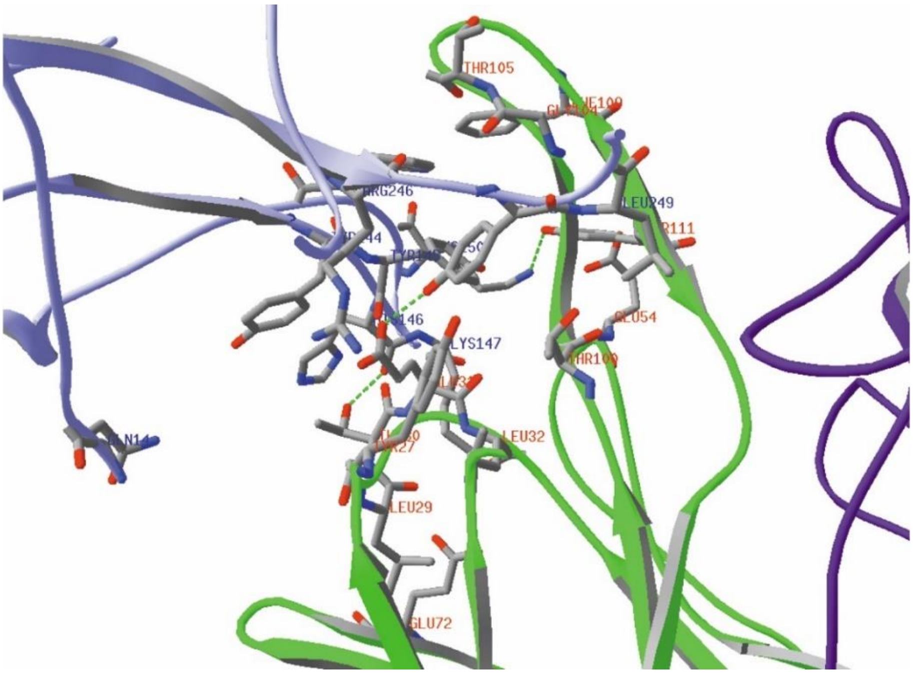

Figure 15. Interaction between the neutralizing antibody Fab fragment and the NTD of SARS-CoV-2 spike protein (figure generated from model 7C2L.pdb). All chains are shown as ribbons (which are reduced to a width of 1 Å). The NTD of spike protein subunit B is colored light blue, while the Fab fragment is colored with its heavy and light chains green and pink, respectively. Residues at the interface are shown with their side chains in CPK colors and labeled (blue for those from the NTD and red for those from the Fab). The N-terminal residue of the NTD (Q^{14}) is shown as well.

How the Fab fragment of antibody CR3022 is able to bind to a SARS-CoV-2 spike protein

The Fab fragment of the mAb CR3022 binds to a different region of the spike protein's RBD than most other neutralizing antibodies (see **Figure 13** in the main paper). It was discussed by Yuan et al. (2020b) that, in order to be able to bind to the spike protein, the latter should have two of its subunits in the open form. **Figure 16** of this Supplementary Material shows that the Fab of CR3022 would be unable to bind to the spike protein trimer when all subunits are in closed state, or when only one is in the open form. Indeed, the Fab is in all cases clashing with different regions in the spike trimer. To produce the images in **Figure 16** of this Supplementary Material, an overlay was made between the RBD in model 6W41.pdb with the RBD in the different chains of model 6YVB.pdb, successively using chain B (which is in open state), and chains A and C (which are in closed state), and with chain B of model 6VXX.pdb.

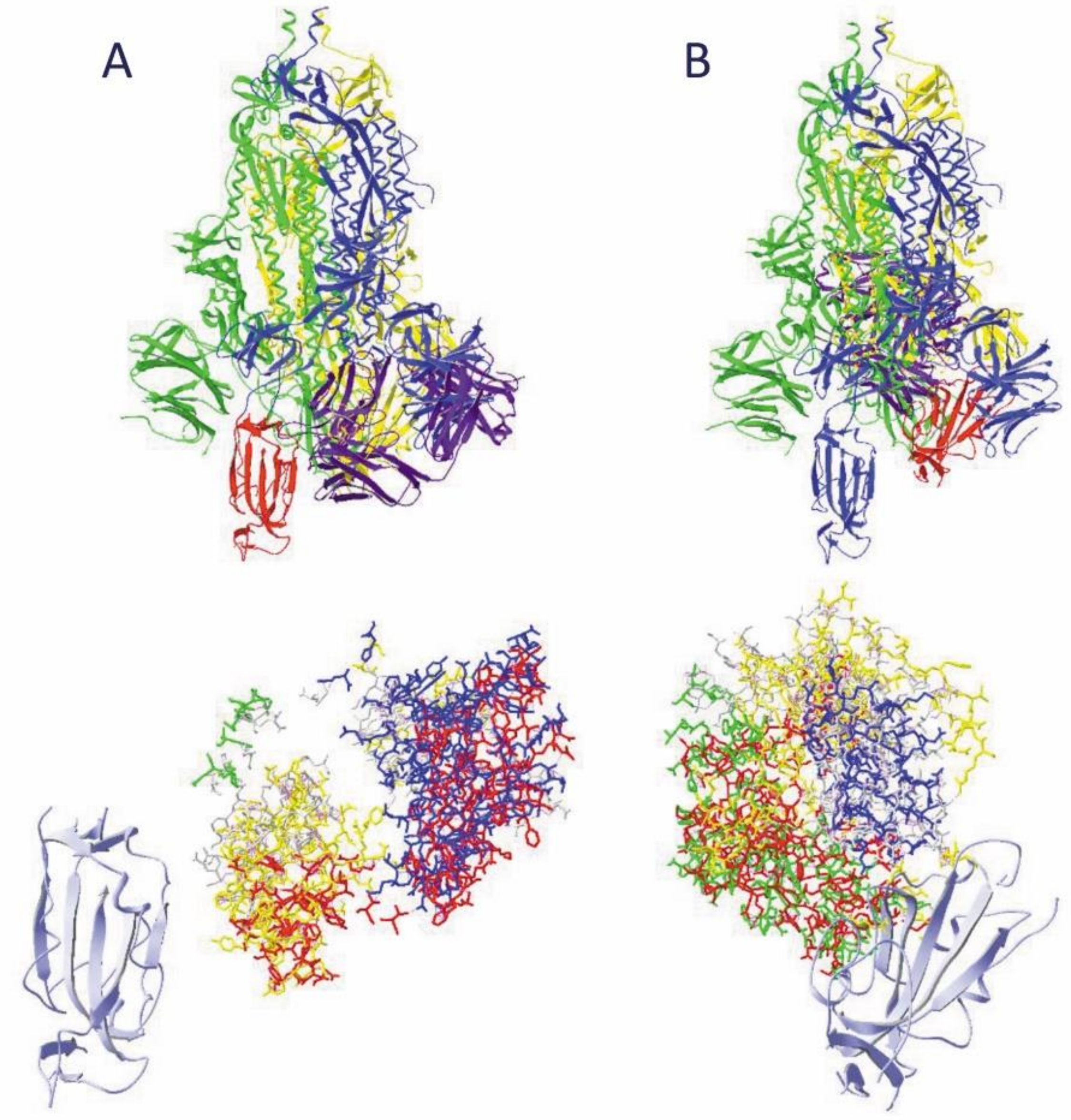

Figure 16. (*continues on the next page*)

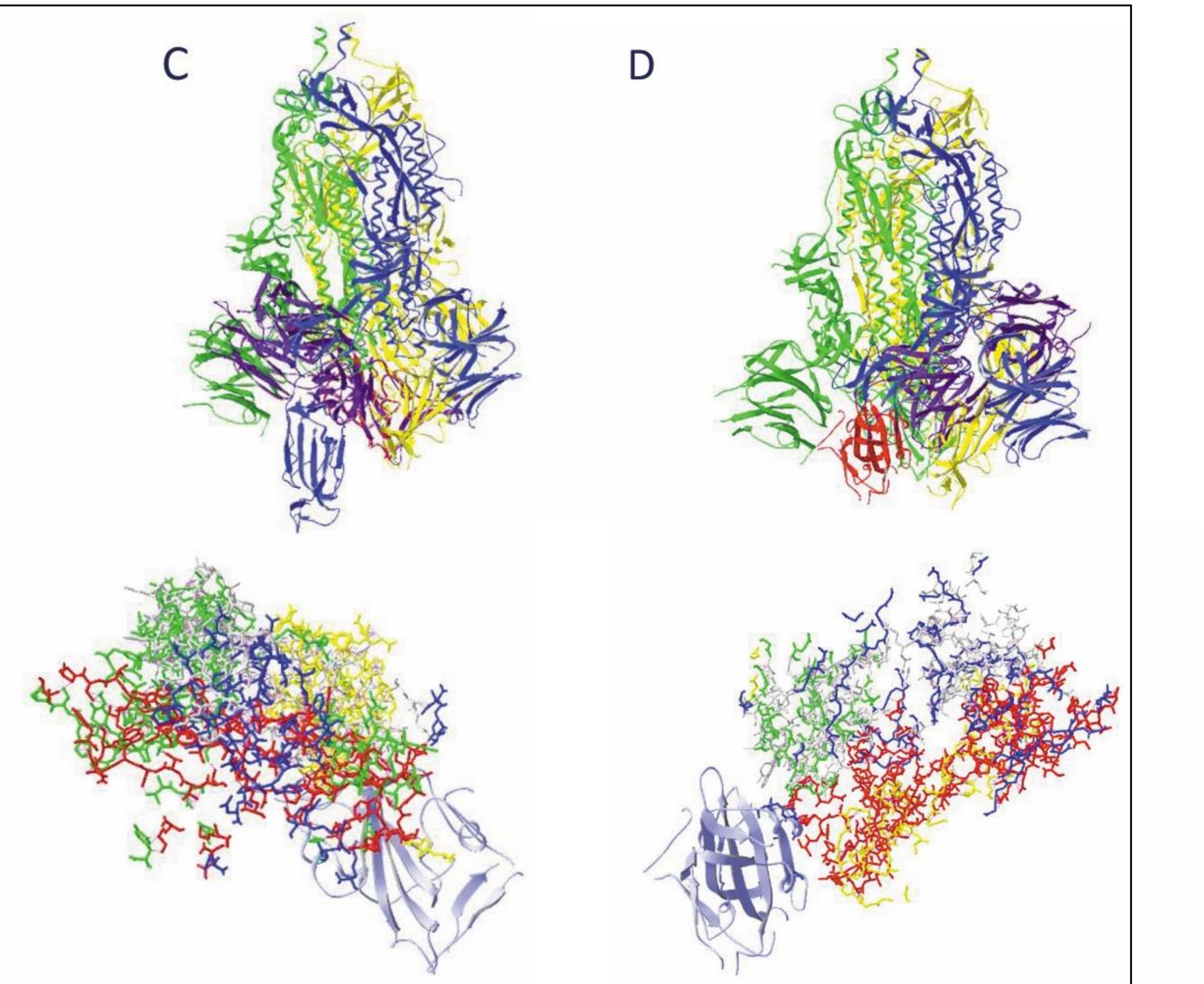

Figure 16. (**A**, **B**, **C**) The SARS-CoV-2 spike protein trimer (from model 6VYB.pdb, chain B in open state and chains A and C in closed state) with an overlay of the Fab from mAb CR3022 (model 6VW1.pdb). The structures on top show the full spike protein, ribbons colored yellow, blue and green (for chains A, B and C, respectively) and the RBD of the chain to which the Fab should be bound in red. The structures underneath show again the RBD (now with ribbons colored light blue) together with residues that would give clashes. The backbones and side chains are colored red, grey, yellow, blue and green when they belong to the Fab H- or L-chain, or the spike protein chains A, B and C, respectively. (**D**) The SARS-CoV-2 spike protein trimer (from model 6VXX.pdb, all chains in closed state) with an overlay of the Fab from mAb CR3022, using the same color codes as above. The structure on top is the full spike trimer, the structure underneath shows again the RBD together with residues that would give clashes. All structures are put in roughly the same orientation.

OTHER VIRAL MEMBRANE PROTEINS

The abundant membrane protein M

The multiple sequence alignment of M proteins from SARS-CoV-2, SARS-CoV and MERS-CoV is shown in **Figure 17** of this Supplementary Material. So far, there are no structural data available for protein M from any of the coronaviruses. Therefore, we can only rely on predictions. The SARS-CoV-2 protein M with accession number QIC53216 was used for the following analyses:

- The presence of a signal peptide, directing the protein to the export route, which starts by allowing its co-translational uptake in the ER lumen, is predicted by the program SignalP to be found at: http://www.cbs.dtu.dk/services/SignalP/ (Nielsen et al., 1997; Armenteros et al., 2019).
- The prediction of transmembrane helices (TM domains) may be done using different programs, which sometimes give varying results. Popular programs are:
  TMHMM (http://www.cbs.dtu.dk/services/TMHMM-2.0/) (Krogh et al., 2001);
  HMMTOP (http://www.enzim.hu/hmmtop/) (Tusnády and Simon, 1998);
  SOSUI (http://harrier.nagahama-i-bio.ac.jp/sosui/sosui_submit.html) (Hirokawa et al., 1998);
  Phobius at the EMBL-EBI server (https://www.ebi.ac.uk/Tools/pfa/phobius/) (Käll et al., 2004). The latter program predicts signal peptides at the same time.
- N-glycosylation is predicted using NetNGlyc, to be found at: http://www.cbs.dtu.dk/services/NetNGlyc/ (Gupta and Brunak, 2002).
- A secondary structure prediction is done by the program JPred4, which may be found at: https://www.compbio.dundee.ac.uk/jpred/ (Drozdetskiy et al., 2015).

Figure 18 of this Supplementary Material shows a prediction of transmembrane helices by the program Phobius (**A**), indicating the presence of three TM domains (in grey). This analysis also confirms the absence of a signal peptide (the probability is zero in **Figure 18A**). The N-terminal domain (19 residues) carries the single N-glycosylation site (indicated with a red arrow). The long C-terminal endodomain (110 residues, of which the first part A^{104}-H^{125} is shown in the sequence at the bottom) is predicted by the program JPred4 to mainly consist of beta-strands, as indicated by the green arrows at the top of the figure (**B**). No coiled-coil regions were predicted by this program. The program IUPred does not predict any disordered regions in this protein.

```
CLUSTAL O(1.2.4) multiple sequence alignment

QIC53216        MADSNGTITVEELKKLLEQWNLVIGFLFLTWICLLQFAYANRNRFLYIIKLIFLWLLWPV    60
AAP13444        -MADNGTITVEELKQLLEQWNLVIGFLFLAWIMLLQFAYSNRNRFLYIIKLVFLWLLWPV    59
AGH58718        -MSNMTQLTEAQIIAVIKDWNFAWSLIFLLITIVLQYGYPSRSMTVYVFKMFVLWLLWPS    59
                   .   :*  ::  ::::**:. .::**    :**:.*  .*.  :*::*:..******

QIC53216        TLACFVLAAVYRINWITGGIAIAMACLVGLMWLSYFIASFRLFARTRSMWSFNPETNILL    120
AAP13444        TLACFVLAAVYRINWVTGGIAIAMACIVGLMWLSYFVASFRLFARTRSMWSFNPETNILL    119
AGH58718        SMALSIFSTVYPIDLASQIISGIVAAVSAMMWISYFVQSIRLFMRTGSWWSFNPETNCLL    119
                ::*  ::::** *:  :  *:  :*.: .:**:***: *:*** ** * ******** **

QIC53216        NVPLHGTILTRPLLESELVIGAVILRGHLRIAGHHLGRCDIKDLPKEITVATSRTLSYYK    180
AAP13444        NVPLRGTIVTRPLMESELVIGAVIIRGHLRMAGHPLGRCDIKDLPKEITVATSRTLSYYK    179
AGH58718        NVPFGGTTVVRPLVEDSTSVTAVVTNGHLKMAGMHFGACDYDRLPNEVTVAKPNVLIALK    179
                ***: ** :.***:*..  : **: .***::**  :* ** . **:*:***. ..*   *

QIC53216        LGASQRVAGDSGFAAYSRYRIGNYKLNTDHSSSSDNIALLVQ                      222
AAP13444        LGASQRVGTDSGFAAYNRYRIGNYKLNTDHAGSNDNIALLVQ                      221
AGH58718        MVKRQSYGTNSGVAIYHRYKAGNYRSPPITADI--ELALLRA                      219
                :   *  . :**.* * **: ***:     :.   ::***
```

Figure 17. Multiple sequence alignment of protein M molecules from SARS-CoV-2, SARS-CoV and MERS-CoV (accession numbers QIC53216, AAP13444 and AGH58718, respectively) using Clustal omega.

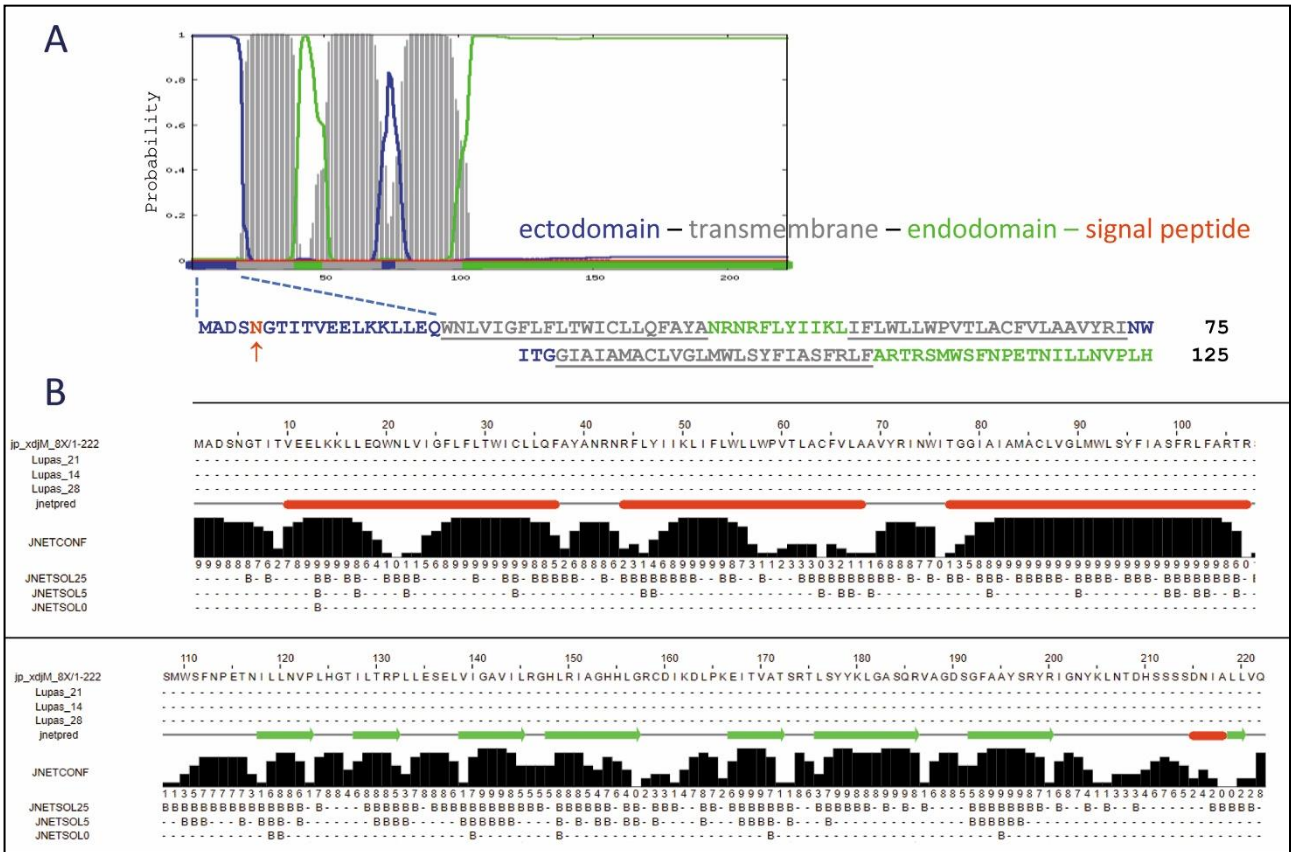

Figure 18. Analysis of SARS-CoV-2 protein M (NCBI accession number QIC53216) using different programs predicting the presence and location of: (**A**) transmembrane helices (using Phobius); the red arrow points to the asparagine residue (N^5 in the sequence NGT) that is potentially N-glycosylated, and (**B**) secondary structure elements including coiled-coil regions (using JPred4). Red cylinders and green arrows represent α-helices and β-strands, respectively. The reliability of the prediction by JPred4 is indicated next to 'JnentConf' for the different helices and strands at each position by a number (1-9), higher numbers representing higher confidence. Coiled-coil prediction is found through the lines indicated as 'Lupas'. 'JnetSol25', 'JnetSol5' and 'JnetSol0' is a prediction of burial of the corresponding amino acid (less than 25% solvent accessibility, less than 5% exposure, 0% exposure, respectively).

The minor membrane component protein E

Each subunit of the homopentameric protein E is generally accepted to be a single-pass membrane protein with a short N-terminal peptide, followed by a TM helix and a longer C-terminal domain, but the exact location of N- and C-termini is still a matter of debate. Different TM helix prediction programs obviously lead to different results (**Figure 19** of this Supplementary Material). The Phobius, TMHMM and HMMTOP programs all predict one single TM helix of about 23 to 26 residues in length in the protein E of the three SARS-CoV-s. Especially the orientation is ambiguous. Indeed, Phobius predicts the N-terminal domain to have a higher probability to be inside the virus than outside (with roughly a 60/40 chance for all three SARS-CoV-s). TMHMM as well attributes to all three proteins a higher probability to have their N-terminal domain in the cytoplasm, while HMMTOP predicts the N-terminus to be inside the virus for SARS-CoV-2 and SARS-CoV but outside the virus for MERS-CoV. The SOSUI server on the other hand predicts two primary TM helices of 23 residues each, only separated from each other by 4-5 amino acid residues.

Figure 20 of this Supplementary Material shows the structure of SARS-CoV-2 protein E (accession number 5X29.pdb). This structure was determined by NMR. In **A** and **B**, all 16 models available are shown, while figures **C** till **F** only show one of the models.

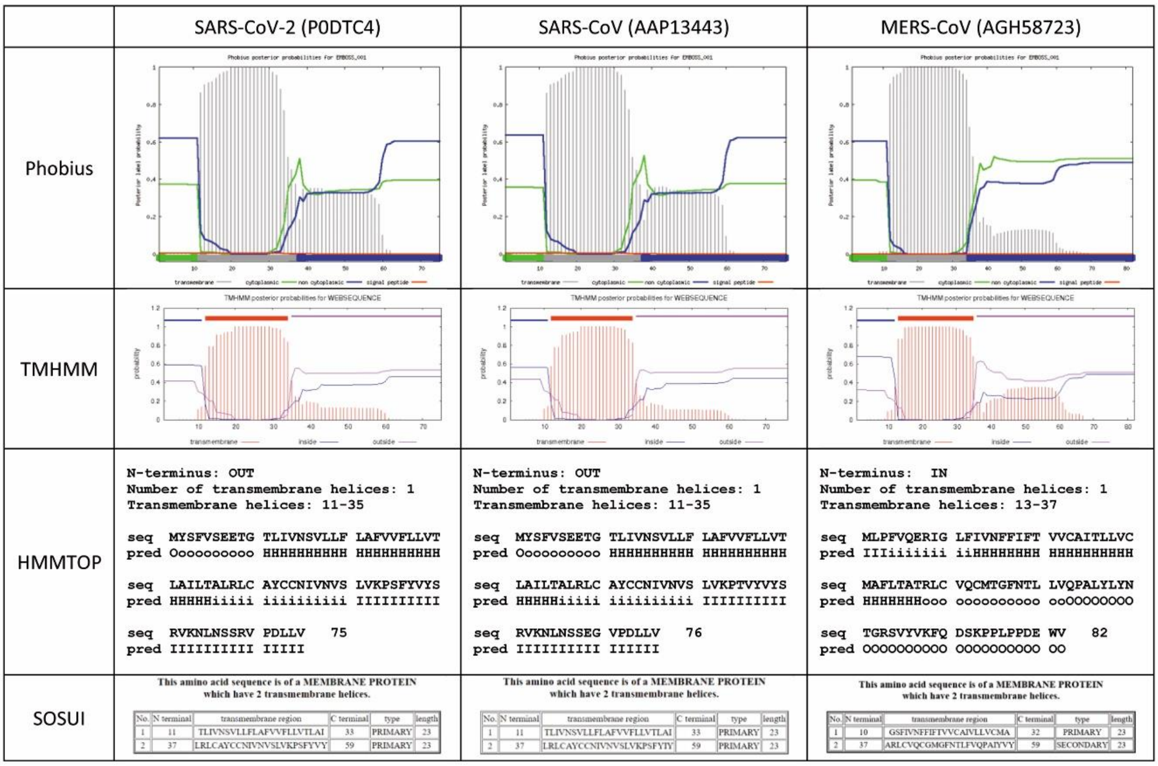

Figure 19. The use of four different TM helix predicting programs on protein E from SARS-CoV-2, SARS-CoV and MERS-CoV. An orientation "in" for a domain indicates that this part of the protein is located in the cytoplasm. The program HMMTOP also predicts whether both regions that are flanking a TM helix in sequence are localized close to the membrane (indicated in small letters) or not in touch with the membrane (capital letters). In SOSUI, "primary" indicates that the peptide would form a TM helix without any help from other TM helices.

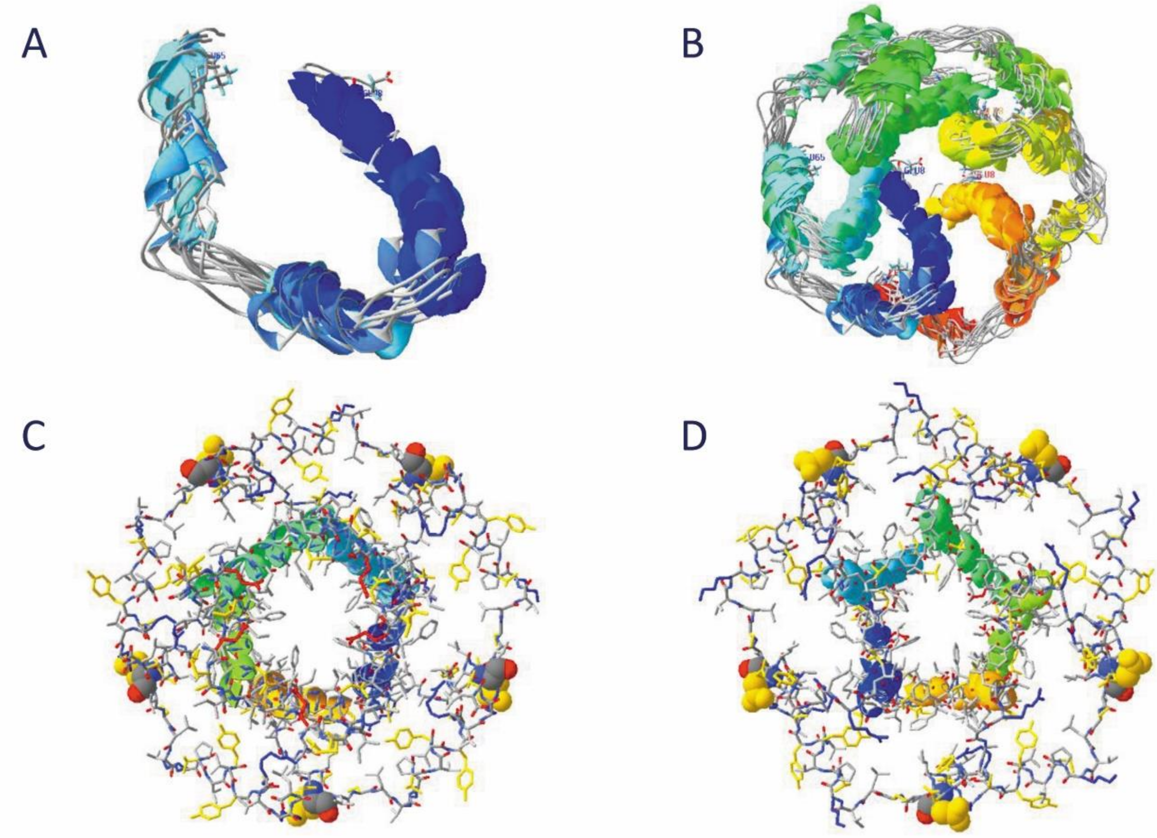

Figure 20. (*continues on the next page*)

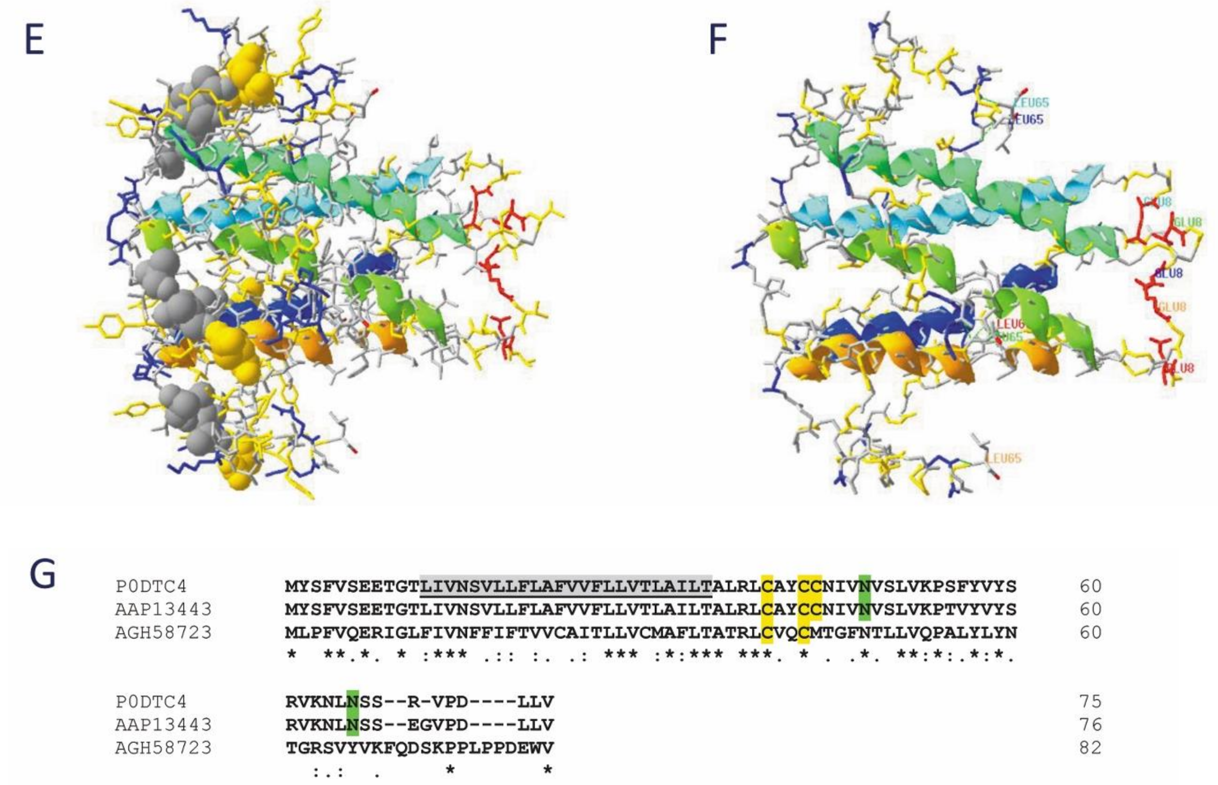

Figure 20. (**A**) The sixteen models (using 5X29.pdb) of chain A, represented as ribbons colored for secondary structure succession. (**B**) The 16 models of all five chains. In the first model of the 16, the N-and C-terminal residues are shown and labeled. In these models, the hydrogen atoms are exceptionally visible (turquoise). (**C-F**) In one of the 16 models, the transmembrane helices (L^{12}-T^{35}) are shown as ribbons colored for secondary structure succession. All amino acids are displayed with their backbone and side chains and colored according to their properties, i.e., hydrophobic, hydrophilic, acidic and basic residues are grey, yellow, red and blue, respectively. Residues N^{48}, one of the potential glycosylation sites, are indicated with their van der Waals surfaces; N^{66} is not part of the structure. In **C** and **D** (flipped by 180°), the viral membrane is supposed to be in the plane of the figure, while **E** and **F** are side views. In picture **E**, also the location of the cysteine residues that may carry palmitoyl chains are indicated with grey-colored van der Waals surfaces. In picture **F**, only the backbones of the five chains are shown, to which are added the side chains of the first and the last residues of each chain. These are labeled blue, turquoise, green, orange and red when belonging to chain A, B, C, D and E, respectively. (**G**) Multiple sequence alignment using Clustal omega of, from top to bottom, protein E from SARS-CoV-2, SARS-CoV and MERS-CoV. The potential N-glycosylation and S-palmitoylation sites are highlighted in green and yellow, respectively. The predicted TM helix is highlighted in grey in the SARS-CoV-2 sequence.

WRAPPING UP THE VIRAL RNA

The soluble nucleocapsid protein N

The nucleocapsid protein N, the only soluble structural protein, is composed of two domains that both bind viral RNA. The N-terminal domain is monomeric, while the C-terminal domain forms dimers. In SARS-CoV-2, as well as in SARS-CoV and in MERS-CoV, both domains are covalently attached to each other through a peptide that is rich in serine- and arginine-residues. To bind the viral RNA, both domains have a high concentration of basic and aromatic residues (**Figures 21** and **22** of this Supplementary Material).

Curiously, the nucleocapsid protein N is predicted to contain extensive stretches of IDRs (**Figure 23** of this Supplementary Material). The presence of IDRs (intrinsically disordered regions) is predicted by the program IUPred, which may be found at: https://iupred2a.elte.hu/ (Mészáros et al., 2018).

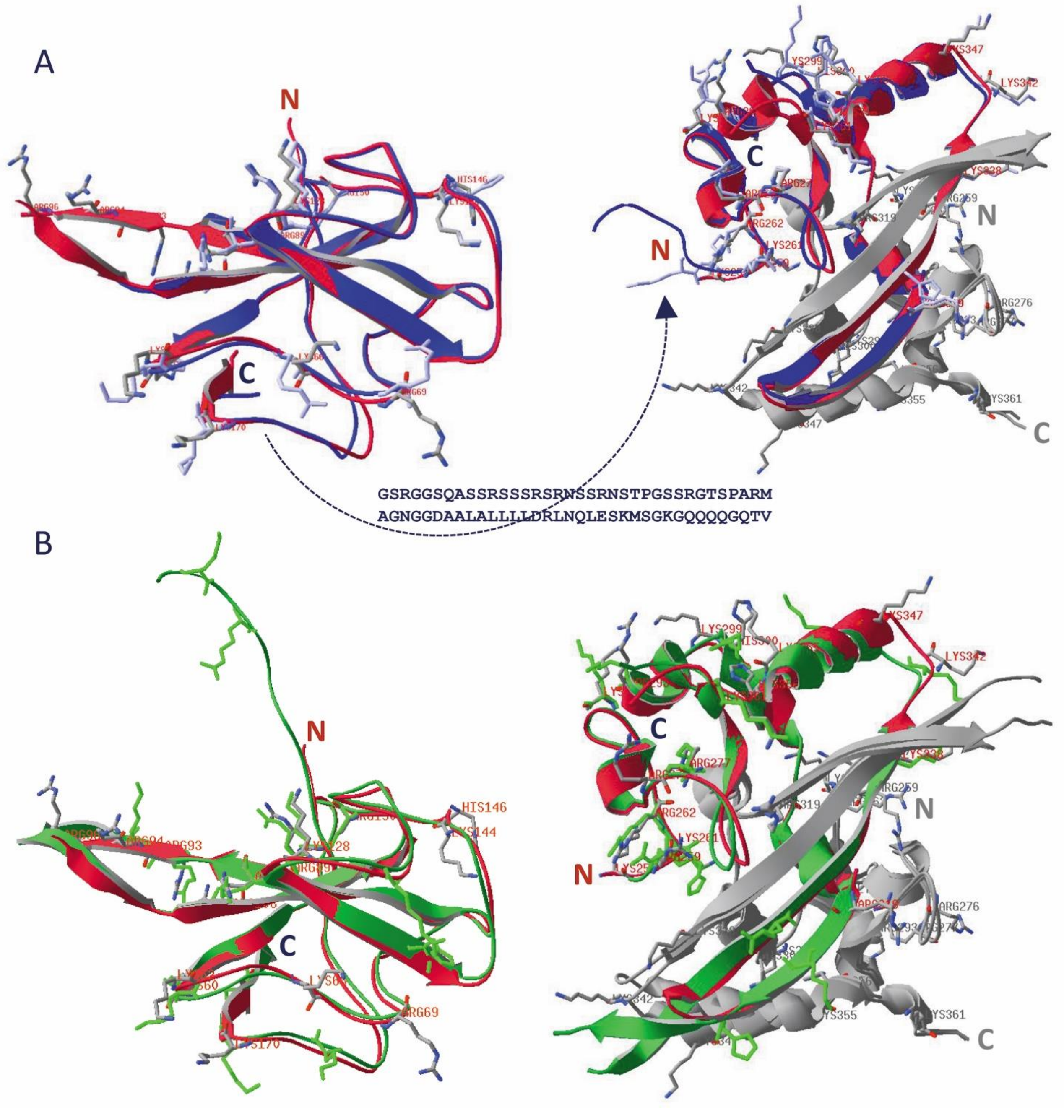

Figure 21. (**A**) Overlay (done manually) of the N-terminal (left) and C-terminal (right) domains of SARS-CoV-2 and SARS-CoV N proteins. Models 6M3M.pdb and 2OG3.pdb (chains A) were used for the N-terminal domain, and models 6WJI.pdb and 2CJ3.pdb (chains A and B) for the C-terminal dimerization domain. Ribbons of the SARS-CoV-2 chains are colored red, and those of chain A the SARS-CoV chain blue, while chain B is colored grey. The side chains of all basic residues are shown in CPK colors and labeled for the SARS-CoV-2 chains, and in light blue for the SARS-CoV chains. The C-terminus of the N-terminal domain is linked to the N-terminus of the C-terminal domain (as indicated with an arrow) by the arginine-serine-rich linker, of which the sequence is shown for the SARS-CoV-2 protein. (**B**) Overlay (done manually) of the N-terminal (left) and C-terminal (right) domains of SARS-CoV-2 and MERS-CoV N proteins. Models 6M3M.pdb and 4UD1.pdb (chains A) were used for the N-terminal domain, and models 6WJI.pdb (chains A and B) and 6G13.pdb (chains A and C) for the C-terminal dimerization domain. Ribbons of the SARS-CoV-2 chains are colored red, and those of chain A the MERS-CoV chain green, while chain B is colored grey. The side chains of all basic residues are shown in CPK colors and labeled for the SARS-CoV-2 chains, and in light blue for the SARS-CoV chains.

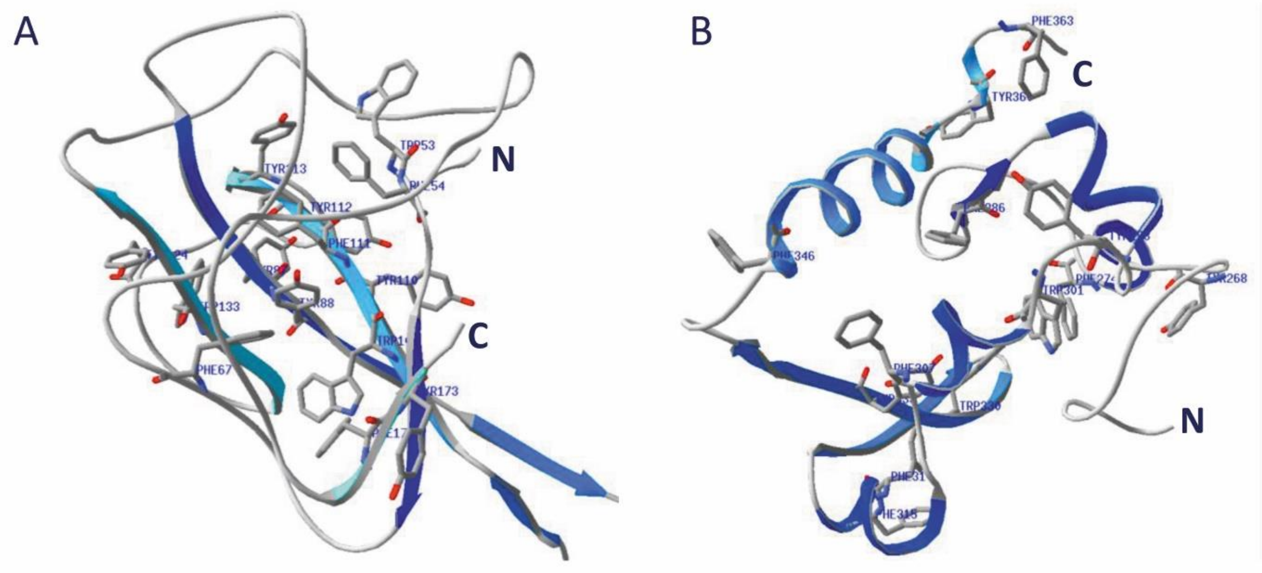

C

```
CLUSTAL O(1.2.4) multiple sequence alignment

P0DTC9          MSDNGPQ-NQRNAPRITFGGPSDSTGSNQNGERSGARSKQRRPQGLPNNTASWFTALTQH      59
AAP13445        MSDNGPQSNQRSAPRITFGGPTDSTDNNQNGGRNGARPKQRRPQGLPNNTASWFTALTQH      60
AGG22549        ------MASPAAPRAVSFADNNDITNTN----LSRGRGRNPKPRAAPNNTVSWYTGLTQH      50
                        .      ::*.. .* *..*     . .* :: :*:. ****.**:*.****

P0DTC9          GKEDLKFPRGQGVPINTNSSPDDQIGYYRRATRRIRGGDGKMKDLSPRWYFYYLGTGPEA      119
AAP13445        GKEELRFPRGQGVPINTNSGPDDQIGYYRRATRRVRGGDGKMKELSPRWYFYYLGTGPEA      120
AGG22549        GKVPLTFPPGQGVPLNANSTPAQNAGYWRRQDRKINTGNG-IKQLAPRWYFYYTGTGPEA      109
                **  * ** *****:*:** * :: **:**  *::. *:* :*:*:******* ******

P0DTC9          GLPYGANKDGIIWVATEGALNTPKDHIGTRNPANNAAIVLQLPQGTTLPKGFYAEGSRGG      179
AAP13445        SLPYGANKEGIVWVATEGALNTPKDHIGTRNPNNNAATVLQLPQGTTLPKGFYAEGSRGG      180
AGG22549        ALPFRAVKDGIVWVHEDGATDAPS-TFGTRNPNNDSAIVTQFAPGTKLPKNFHIEGTGGN      168
                .**: * *:**:**  :** ::*.  :***** *::* * *:  **.***.*: **: *.

P0DTC9          SQASSRSSSRSRNSSRNSTPGSSRGTSPARMA---GNGGDAALALLLLDRLNQLESKMSG      236
AAP13445        SQASSRSSSRSRGNSRNSTPGSSRGNSPARMA---SGGGETALALLLLDRLNQLESKVSG      237
AGG22549        SQSSSRASSVSRNSSRSSSQGSRSGNSTRGTSPGPSGIGAVGGDLLYLDLLNRLQALESG      228
                **:***:** **..**.*: **  *.*    :   .. * ..  ** ** **:*::  **

P0DTC9          KGQQQQGQTVTKKSAAEASKKPRQKRTATKAYNVTQAFGRRGPEQTQGNFGDQELIRQGT      296
AAP13445        KGQQQQGQTVTKKSAAEASKKPRQKRTATKQYNVTQAFGRRGPEQTQGNFGDQDLIRQGT      297
AGG22549        KVKQSQPKVITKKDAAAAKNKMRHKRTSTKSFNMVQAFGLRGPGDLQGNFGDLQLNKLGT      288
                * :*.* :.:***.** *.:* *:***:** :*:.**** *** : ****** :* : **

P0DTC9          DYKHWPQIAQFAPSASAFFGMSRIGMEVTP------SGTWLTYTGAIKLDDKDPNFKDQV      350
AAP13445        DYKHWPQIAQFAPSASAFFGMSRIGMEVTP------SGTWLTYHGAIKLDDKDPQFKDNV      351
AGG22549        EDPRWPQIAELAPTASAFMGMSQFKLTHQNNDDHGNPVYFLRYSGAIKLDPKNPNYNKWL      348
                :  :*****::**:****:***:: :             :* * ****** *:*:::. :

P0DTC9          ILLNKHIDAYKTFPPTEPKKDKKKKADET--QALPQ--------RQKKQQTVTLLPAADL      400
AAP13445        ILLNKHIDAYKTFPPTEPKKDKKKKTDEA--QPLPQ--------RQKKQPTVTLLPAADM      401
AGG22549        ELLEQNIDAYKTFPKKEKKQKAPKEESTDQMSEPPKEHRVQGTQRTRTRPSVQPGPMIDV      408
                 **:::******** .* *:.  *: .    .  *:        * :.: :*   *  *:

P0DTC9          DDFSKQLQQSMSSADSTQA--                                            419
AAP13445        DDFSRQLQNSMSGASADSTQA                                            422
AGG22549        NTD------------------                                            411
                :
```

Figure 22. Abundant presence of aromatic amino acid residues in the N-terminal (**A**) (model 6M3M, chain A) and C-terminal (**B**) (model 6WJI, chain A) domains of SARS-CoV-2 protein N. The ribbons are colored for secondary structure succession. All aromatic residues are shown with their side chains and labeled. The width of the ribbons was reduced to 1 Å to make the side chains on display better visible. The N- and C-termini of the chains are indicated (done manually). (**C**) Multiple sequence alignment of, from top to bottom, protein E from SARS-CoV-2, SARS-CoV and MERS-CoV. Peptides that are underlined in the SARS-CoV-2 sequence are predicted to be intrinsically disordered (see **Figure 23** of this Supplementary Material).

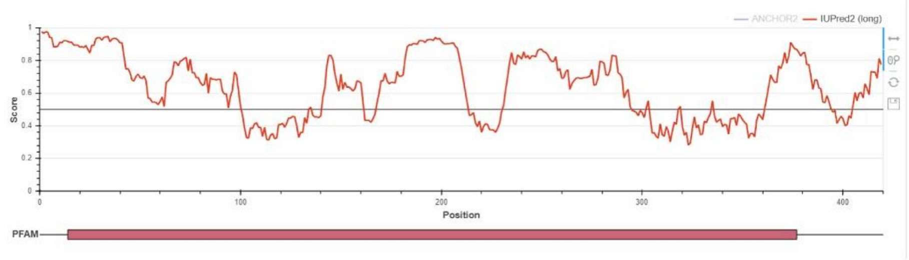

Figure 23. Analysis of disorder in the nucleocapsid protein N from SARS-CoV-2 (accession number P0DTC9) using the program IUPred. Residues that received a score higher than 0.5 (those above the threshold line) are predicted to be disordered.

RECENTLY DISCOVERED SARS-COV-2 VARIANTS OF CONCERN WITH MULTIPLE MUTATIONS

In October 2020, two Sar-CoV-2 variants emerged independently from each other, one in the United Kingdom (UK, variant B.1.1.7) and one in South-Africa (SA, variant 501Y.V2, also known as B.1.351). They display increased transmissibility and have multiple but different mutations in their spike proteins, some of which in parts of the RBD that are located close to the ACE2 receptor binding region. There is evidence that all three mutations ($K^{417}N$, $E^{484}K$ and $N^{501}Y$) confer enhanced affinity to the spike protein for ACE2 receptor binding and, moreover, may abolish key interactions between the RBD and certain classes of anti-spike protein antibodies (Tegally et al., 2020). **Figure 24** of this Supplementary Material highlights the position of the mutations in the UK (**A**) and the SA (**B**) variants, respectively. In addition, a new variant (P1, descendant of lineage B.1.1.28) was further discovered in Brazil that independently acquired a series of mutations, some of which ($K^{417}T$, $E^{484}K$ and $N^{501}Y$) are similarly found in the SA variant (Naveca et al., 2021; Sabino et al., 2021).

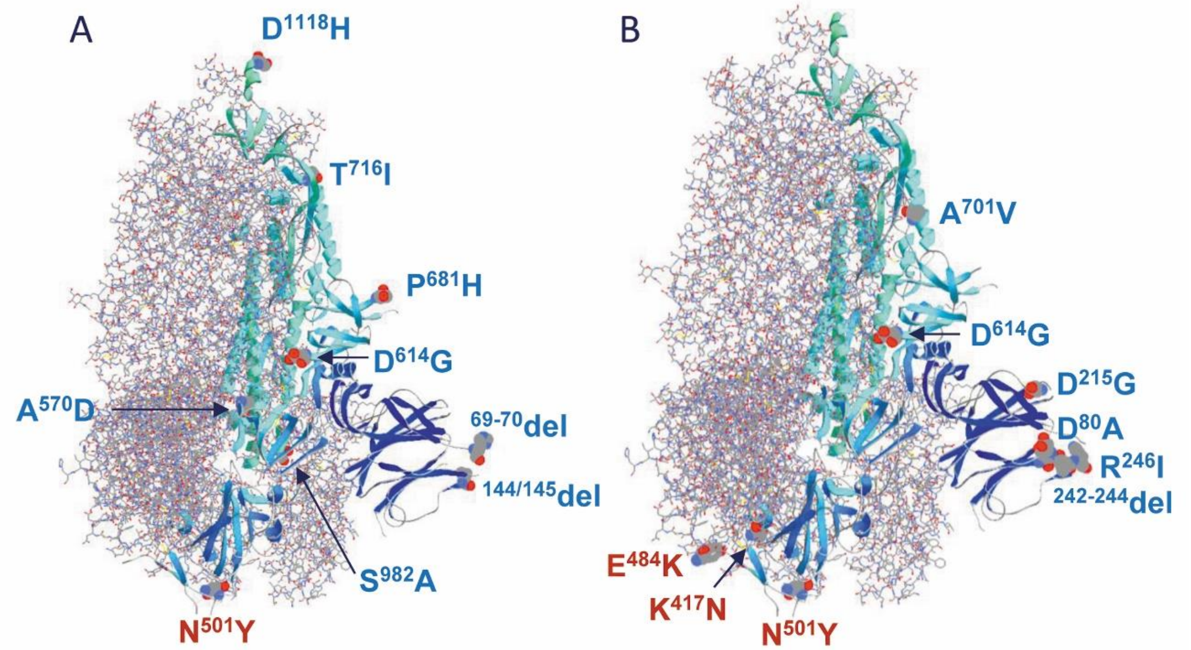

Figure 24. Position of the mutations/deletions in the United Kingdom (**A**) and the South-African (**B**) variants of the SARS-CoV-2 spike proteins that recently emerged. In the spike protein (model 6VXX.pdb), subunit A is represented as ribbons (without showing residues' backbones and side chains), colored for secondary structure succession, while in subunits B and C all residues are displayed with their backbones and side chains in CPK colors. The mutated residues are shown (or an adjacent residue in case the true amino acid itself is missing in the model), only in subunit A, as their van der Waals surface, and labeled manually. Residues belonging to the RBD are labeled in red. Both figures are shown in exactly the same orientation.

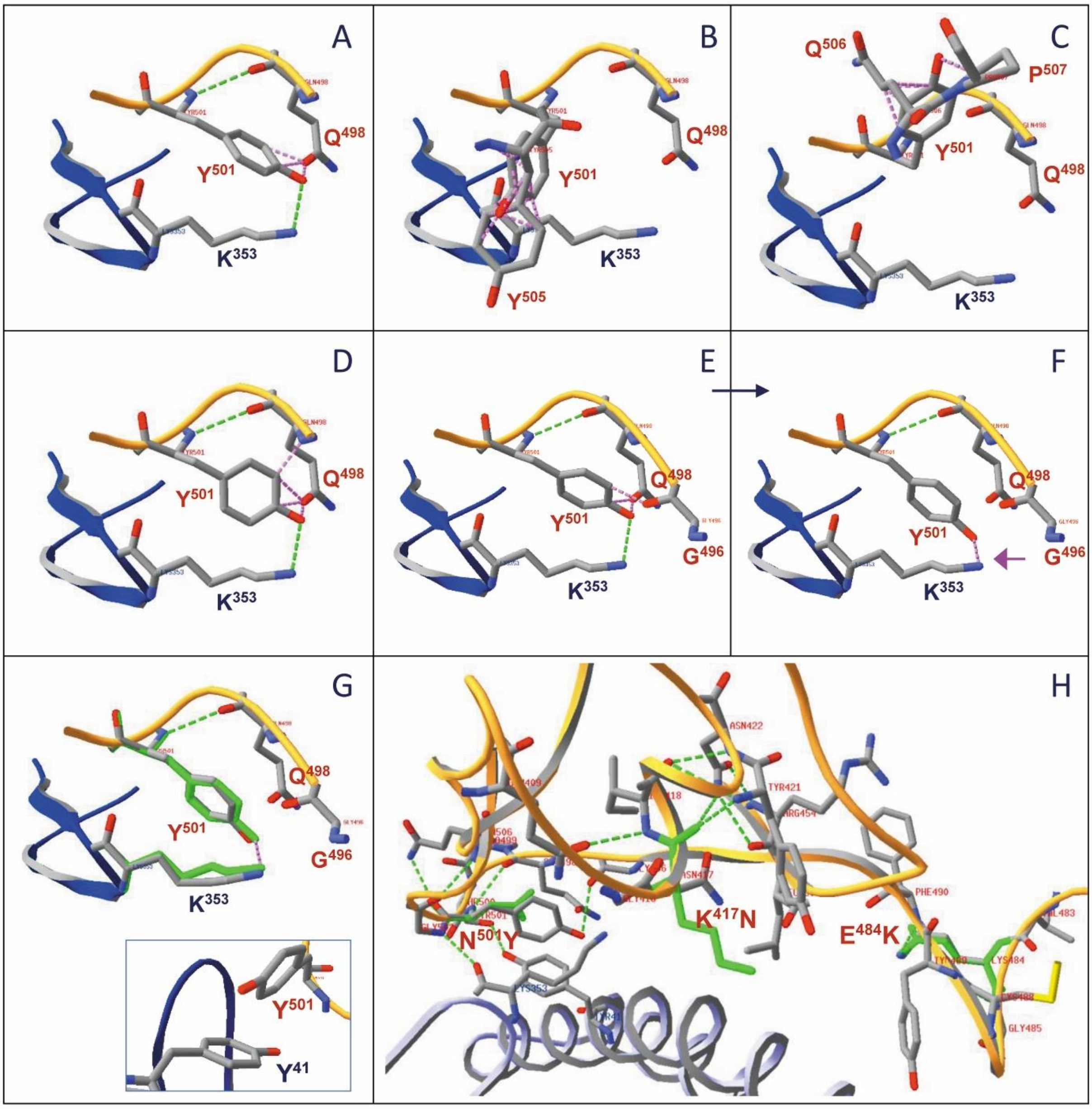

Figure 25. (**A**-**G**) Introduction of the N^{501}Y mutation in the SARS-CoV-2 RBD by DeepView, using model 6M0J.pdf. Clashes (pink dashed lines) are seen between Y^{501} and other residues from the same RBD for all rotamers, i.e.: Q^{498} (**A**), Y^{505} (**B**), Q^{506} and P^{507} (**C**), with Q^{498} (**D**), and with Q^{498} and G^{496} (**E**). In these panels, also the ACE2 residue K^{353} is shown, which is suggested to form a new hydrogen bond with Y^{501} in three of the rotamers. When making use of the torsion angle button, the position of the Y^{501} side chain may slightly be adapted so that no clashes occur anymore with G^{496} and Q^{498}, but now the new hydrogen bond with the ACE2 residue K^{353} became too short (2.13 Å: **F**, pink arrow). Then, the position of the amino acid side chains may be adjusted by using the 'Energy Minimization' button (**G**). Side chains of Y^{501} and K^{353} are shown in green (still showing the clash between them as a pink dashed line) and in CPK colors before and after energy minimization, respectively. The inset shows the relative position of the side chains of the mutated Y^{501} in the RBD and the ACE2 residue Y^{41}, after energy minimization.

(**H**) The position of the three mutations in the South-African variant is shown, with the original residue's backbones and side chains colored green and the ones in the mutants in CPK colors. They are manually labeled. Two extra hydrogen bond linkages are formed between the RBD residues Y^{501} (-OH) and G^{496} (backbone O), and between K^{484} (Nζ) and F^{490} (backbone N), but no extra hydrogen bond becomes apparent in the K^{417}N mutation. Ribbons in this model are colored orange for the RBD and light blue for the ACE2 receptor. Neighbors at 3.5 Å of the three mutated residues are added to the image and labeled (in DeepView), red for those of the RBD and blue for those of the ACE2 chain.

The three mutations in the RBD of the SA variant (of which the $N^{501}Y$ mutation also appears in the UK variant) are analyzed in **Figure 25** of this Supplementary Material, making use of model 6M0J.pdb. However, when creating the common and apparently successful mutation $N^{501}Y$ in the model, some difficulties emerge. Despite the finding that the Y^{501} residue would come close enough to the ACE2 residue K^{353} to allow the formation of a hydrogen bond, all tyrosine side chain rotamers clash with surrounding residues of the RBD, as is shown in panels **A-E**. In these images, all subsequent rotamers of Y^{501} are displayed. The observation that clashes occur in all rotamers indicates that the environment of Y^{501} will have to be somewhat adjusted in the mutant. Unfortunately, no real structural data of the mutation are available so far.

To avoid clashes in the hypothetical model, the torsion angle button in the DeepView Toolbar may be applied on residue Y^{501} (in **Figure 25E** of this Supplementary Material) by dragging the mouse left/right while holding down the ‘1’ or ‘2’ keys, thereby slightly rotating the aromatic side chain about its first (Cα-Cβ) or second (Cβ-Cγ) bond, respectively, thereby pushing the side chain away from Q^{498}. A position could be found in which all clashes with surrounding RBD residues were eliminated, though the newly formed potential hydrogen bond between the side chain -OH of the viral RBD residue Y^{501} and the side chain -Nη of the ACE2 residue K^{353} is now too short (2.13 Å) and becomes a new clash instead (**Figure 25F**). Thereafter, an ‘Energy Minimization’ step was run using the GROMOS96 implementation of DeepView (under ‘Tools’ in the Toolbar). This process takes a little while since calculations need to be made by the program. The result is presented in **Figure 25G**, showing that the positions of Y^{501} (RBD) and K^{353} (ACE2) need only very slight adaptations to create an acceptable overall structure. Finally, also energy calculations may be done by the DeepView program (using ‘Tools’ > ‘Compute Energy (Force Field)’). Each of the structures obtained in the process of making the mutation $N^{501}Y$ was subjected to these calculations and the results are displayed in **Table 2** of this Supplementary Material.

The hypothetical structure of the triple mutant obtained here and presented in **Figure 25H** of this Supplementary Material shows the viral RBD with all neighbors at 3.5 Å of residues N^{417} (i.e., Q^{409}, G^{416}, I^{418}, Y^{421}, N^{422}, R^{454} and L^{455}), K^{484} (i.e., V^{483}, G^{485}, C^{488}, Y^{489} and F^{490}, the latter forming a new hydrogen bond with K^{484}), and Y^{501} (i.e., G^{496}, Q^{498}, P^{499}, T^{500}, G^{502}, Q^{506}, plus the ACE2 residues Y^{41} and K^{353}). Both these ACE2 residues Y^{41} and K^{353} form a hydrogen bond with T^{500} and G^{502} from the RBD, respectively. The mutated residue Y^{501} is making hydrogen bond interactions with G^{496} (through its -OH side chain) and with Q^{498} and with Q^{506}. Moreover, the distance between the centers of the aromatic ring structures of residue Y^{501} in the RBD and Y^{41} in the ACE2 receptor is estimated in DeepView to be around 4.5-4.8 Å. Since they are also nearly perpendicular to each other (see the inset of **Figure 25G** of this Supplementary Material), this should bring about strongly stabilizing π-π interactions (Makwana and Mahalakshmi, 2015), which will contribute to an increased binding strength of a $N^{501}Y$ mutant.

It should be noted that, after running the ‘Energy Minimization’ procedure, hydrogen atoms are automatically made visible in the resulting DeepView model as turquoise lines (**Figure 26A** of this Supplementary Material). Hydrogen bonds are now displayed from the hydrogen bond acceptor atom to the hydrogen attached to the hydrogen bond donor atom, and not anymore to that atom itself. Thereby, hydrogen bond distances, when asked to be shown, become smaller by about 0.7-1 Å. When hydrogen atoms are again removed from the figure (‘Display’ > untick ‘Show Hydrogens’), the bond distances displayed remain however the same, which is visualized in **Figure 26B** of this Supplementary Material.

Table 2. Analysis of energy levels of the different structures while introducing the mutations $N^{501}Y$, followed by $K^{417}N$ and $E^{484}K$ in the RBD of the SARS-CoV-2 spike protein using DeepView.

Structure	Figure	Overall energy level of the whole model* (KJ/mol)	Contribution of some individual residues (KJ/mol)		
			Y^{501}	G^{496}	Q^{498}
Original model (6M0J.pdb)		−36,330	(N^{501}) −170	+24	−169
After mutation $N^{501}Y$	25E	+630,197	+4,579	+333,162	+328,416
After modifying torsion angle in Y^{501} (manually)	25F	−27,172	+4,141	+3,654	−114
After energy minimization	25G	−35,047	+226	+163	−140
After adding mutations $K^{417}N$ and $E^{484}K$	25H	−35,153	+226	+163	−140

*The whole model comprises 587 residues in chain A and 194 residues in chain E.
Extremely high positive, thus unacceptable, values are put in red.

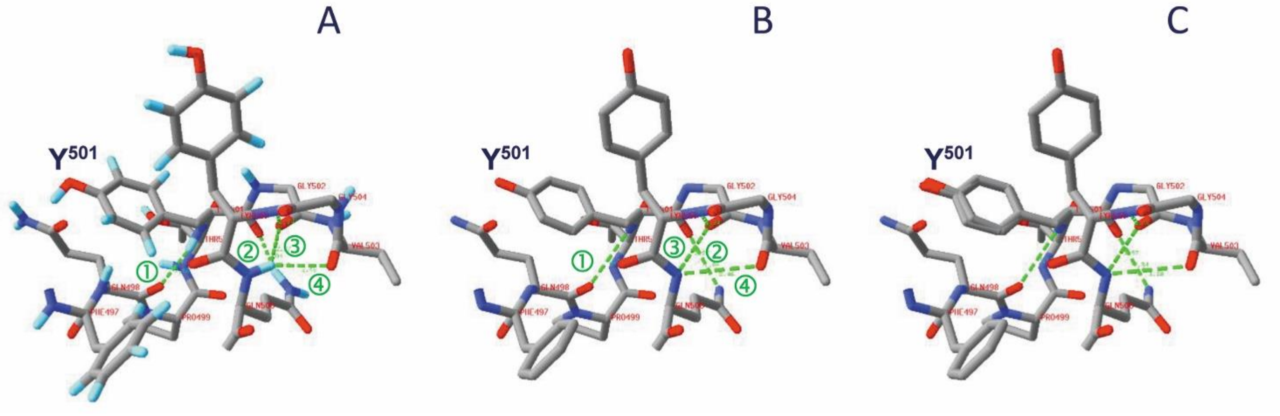

Figure 26. (**A**) Structure of the stretch F[494] till Q[506] in the RBD in model 6M0J.pdb after introduction of the N[501]Y mutation and performing the energy minimization step, upon which hydrogen atoms become visible in DeepView. Four hydrogen bonds are numbered. (**B**) Same model, but hydrogen atoms were removed from the image. The same four hydrogen bonds are numbered in the same way. (**C**) **Figure 26B**, superposed on the model obtained before energy minimization, showing the same four hydrogen bonds. Hydrogen bond distances, as indicated by DeepView, are, in **A** as well as in **B**: ① 2.16 Å, ② 1.84 Å, ③ 2.27 Å, ④ 2.46 Å; in **C** (where hydrogen atoms are not shown by DeepView): ① 3.15 Å, ② 2.84 Å, ③ 2.97 Å, ④ 3.28 Å, respectively.

LOOKING INTO POSSIBLE MUTATIONS USING DEEPVIEW

In this section we will look to the environment of a phenylalanine, residue F[497], in the SARS-CoV-2 spike protein. We use the model 6VW1 to investigate this residue. **Figure 27** of this Supplementary Material shows the location of residue F[497] in the spike protein's RBD, very close to the long helix in the ACE2 receptor protein that contains several residues with which the viral RBD makes contacts. The location of this F[497] is shown in **Figure 27A** and a close-up is displayed in **Figure 27B**.

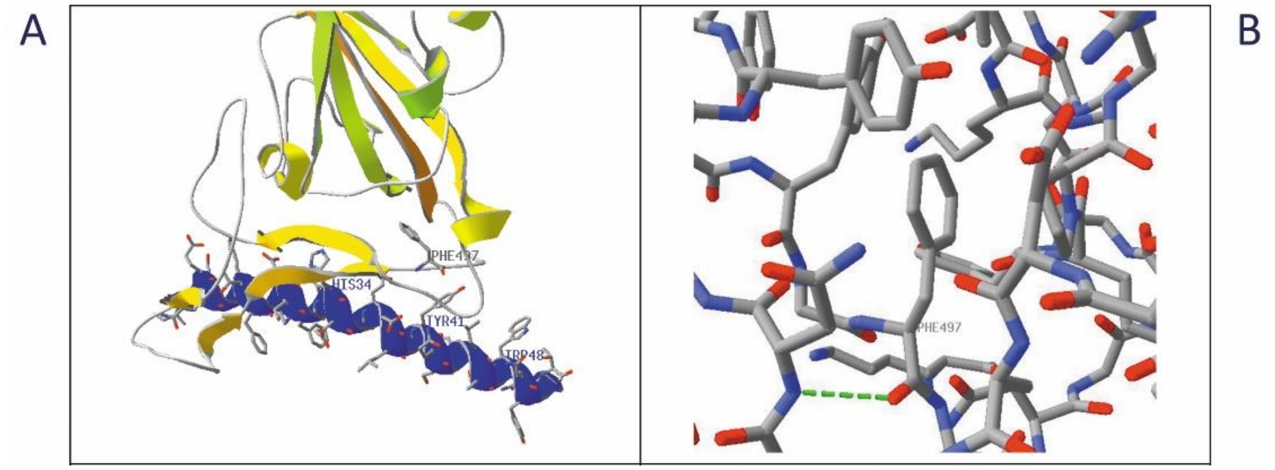

Figure 27. Investigation of the environment of residue F[497] in the SARS-CoV-2 RBD using coordinates from 6VW1.pdb. (**A**) Figure showing the location of this residue in the vicinity of a long helix (colored blue) in the ACE2 receptor. Some residues of this helix that have ring structures facing the RBD are shown with their side chains and labeled in blue. The viral residue F[497] is shown as well, labeled. The β-strands of the RBD are colored for secondary structure succession. (**B**) In this figure, the residue F[497] is put in the center, and surrounding residues at a distance of 10 Å from this phenylalanine were added to the image, which was then zoomed in on F[497].

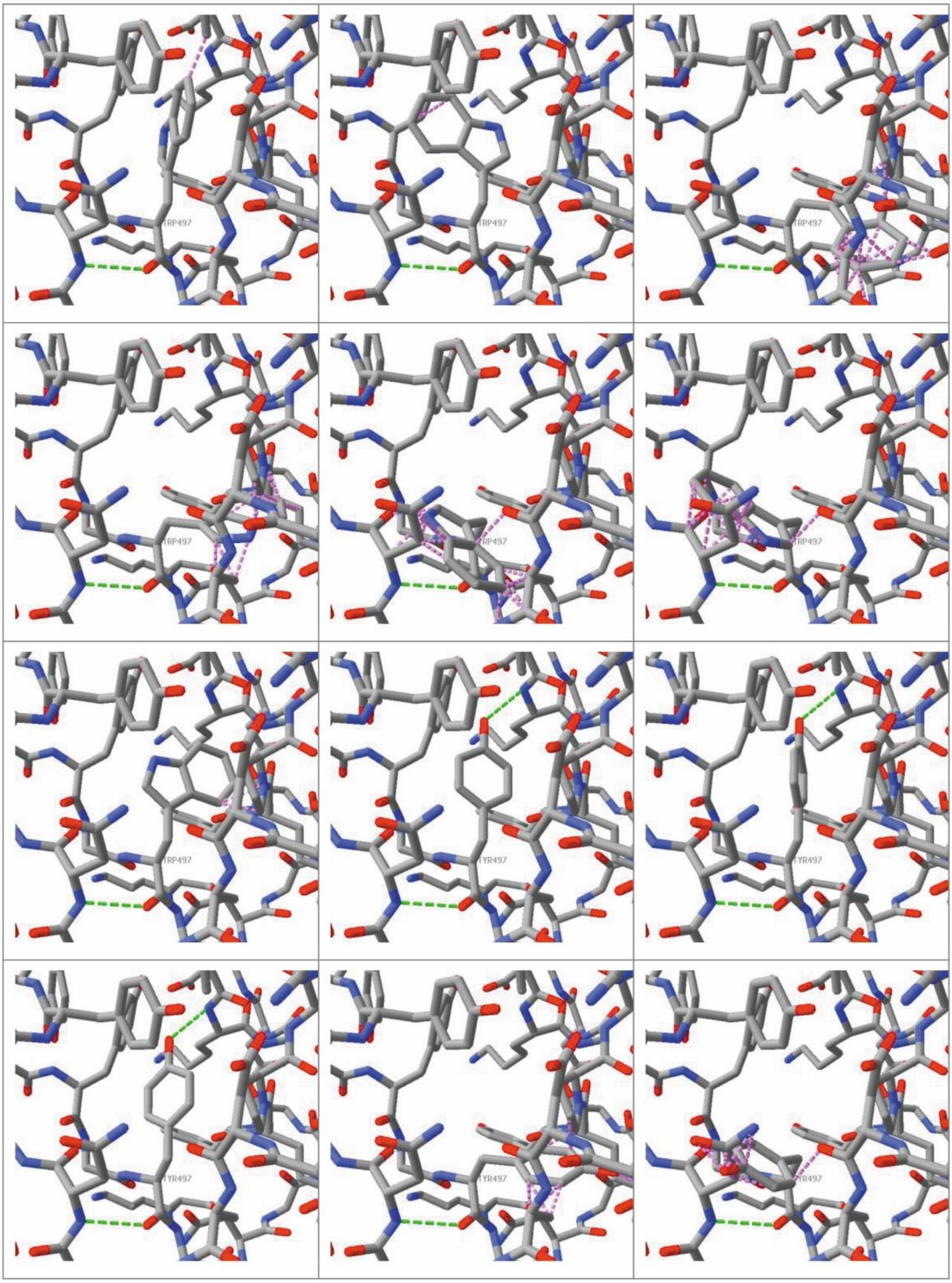

Figure 28. The possible rotamers of a tryptophan and a tyrosine mutation (7 for W and 5 for Y, starting from upper left to lower right) of residue F^{497} in the RBD of the SARS-CoV-2 spike protein, analyzed using model 6VW1.pdb. Hydrogen bonds with residue 497 are shown as green dashed lines. Clashes are shown as pink dashed lines. The residue 497 is labeled in DeepView. In all figures the protein has the same orientation as in **Figure 27B** of this Supplementary Material.

Now we want to investigate, as an example, whether F^{497} could be expected, over time, to mutate into another aromatic residue. To this end, this F^{497} residue was put in DeepView in the center of the picture using model 6VW1.pdb, and surrounding residues at a distance of 10 Å from this phenylalanine were added to the picture, which was then zoomed in on F^{497}. Next, mutations F^{497}W and F^{497}Y were analyzed. For all figures, the orientation of the RBD was not changed. Hydrogen bonds, with the aromatic residue at position 497 only, are shown as green dashed lines. Clashes in the molecule are indicated by pink dashed lines. The result obtained for all rotamers (7 for tryptophan and 5 for tyrosine) is shown in **Figure 28** of this Supplementary Material.

The figure shows that in case of a mutation F^{497}W, all the possible rotamers give clashes (indicated as pink dashed lines) with surrounding residues because of the bulkiness of the tryptophan side chain. Such mutation can thus be expected to interfere with a correct folding of the RBD and to be rejected in Nature. On the other hand, in case of a mutation F^{497}Y, three rotamers do nicely fit in the available space. Moreover, in the three acceptable rotamers an extra hydrogen bond is formed between the Y^{497} side chain -OH and the backbone nitrogen atom of the neighboring K^{403} residue, which may contribute to extra stability of the RBD. This is a mutation that might thus be expected to occur once upon a time in Nature.

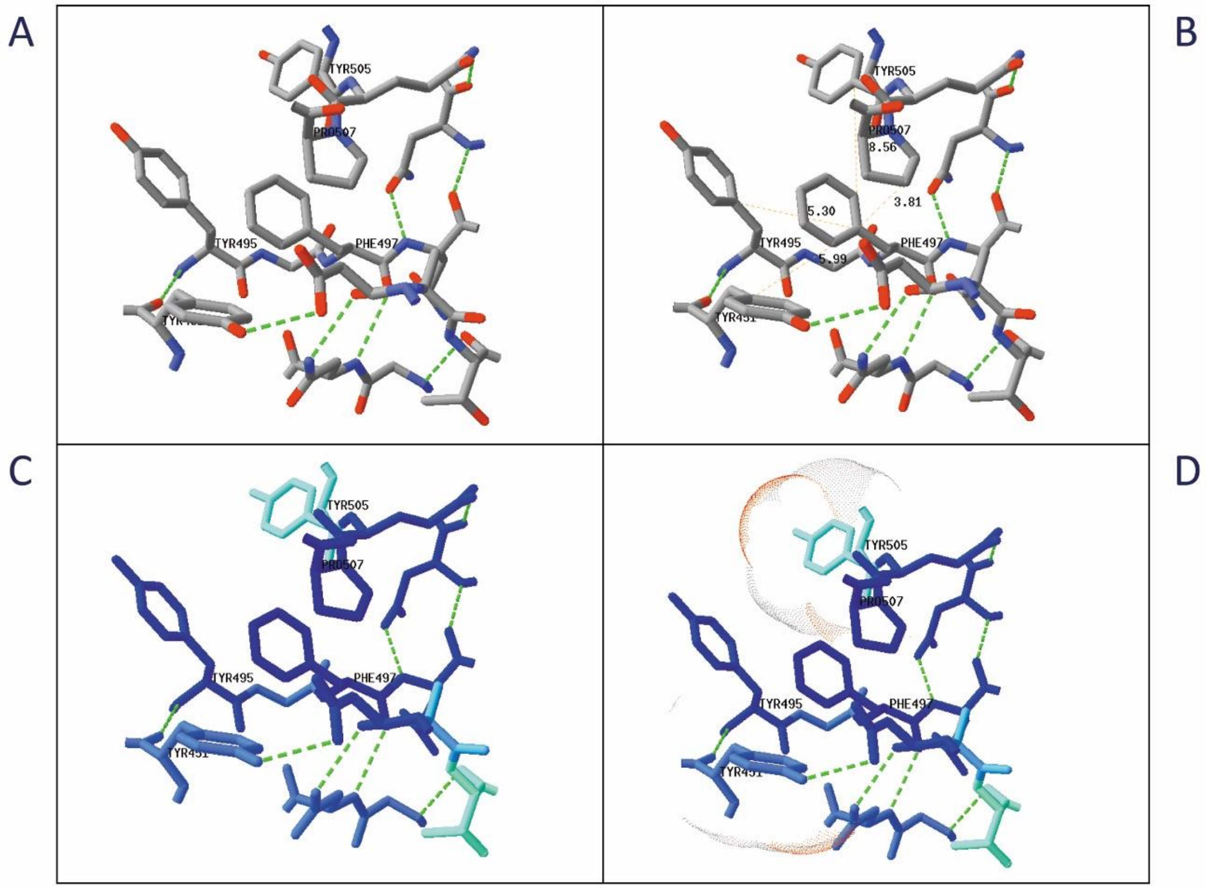

Figure 29. (**A**) Figure showing F^{497}, put in the center and labeled, together with residues that are within a distance of 4 Å (i.e. D^{442}, A^{443}, T^{444}, G^{447}, N^{448}, Y^{451}, Y^{495}, G^{496}, Q^{498}, N^{501}, Y^{505}, Q^{506} and P^{507}, all belonging to chain E). Residues having a ring structure in their side chain are labeled. All hydrogen bonds formed are shown. (**B**) The determination of the distance between the γ-carbon atom of F^{497} and the γC atoms of the ring side chains is presented. (**C**) The same residues are colored for accessibility. (**D**) The accessibility surfaces are added for the residues F^{497}, Y^{451}, Y^{495}, Y^{505}, and P^{507}. All four images A-D show the molecule in the same orientation.

Next, we look to the environment of the same E-chain residue Phe^{497}. With F^{497} still in the center of the picture and labeled, residues that are within a distance of 4 Å from it were added (**Figure 29A** of this Supplementary Material). Four of them have side chains with a ring structure and were labeled. Hydrogen bonds are also shown. As an example, distances are determined between the γ-carbon atom of F^{497} and the γ-carbon atoms of the ring side chains (**Figure 29B**). Also, the nature of the environment of residue F^{497} can be highlighted by

e.g., coloring residues for accessibility (this usually takes a while as the DeepView program needs to make the calculation first). The model reappears in rainbow colors (**Figure 29C**), ranging from dark blue (very inaccessible) to orange (very accessible). The color of each residue is based on the percentage of its surface area that is exposed (accessible) to the surrounding solvent. In addition, accessibility surfaces may further be added to the picture for chosen residues (**Figure 29D**). The solvent-accessible surface lies above each atom at a distance of the van der Waals radius plus 1.4 Å. It includes only the surface with which a spherical solvent molecule with a radius of 1.4 Å (which is approximately the radius of a water molecule) could come into contact. Most residues showing up are hidden inside the protein molecule. Of the labeled residues, only Y^{451} (somewhat lighter blue) and Y^{505} (light blue) are more accessible. This is also obvious after adding their accessibility surface to each of the residues, which is still colored in CPK colors (oxygen red, nitrogen blue, carbon grey) to show which part of the surface is accessible for a water molecule. The accessibility surface of Y^{505} is strikingly wider than that of Y^{451}, but completely absent for F^{497}, Y^{495} and P^{507}, showing that the latter three residues are indeed totally hidden inside the protein's RBD.

USE OF COORDINATES FROM THE PDB DATABASE

The PDB is a repository of structural coordinates of biological macromolecules and may be accessed through NCBI. The files mentioned in **Table 3** of this Supplementary Material were downloaded from the PDB structure database and used for making figures in this paper. The origin of the proteins in these data files is briefly mentioned in footnotes to the table. Most of the structural data were acquired using X-ray crystallography, still today's most popular technique for solving the structure of biological macromolecules. It relies on making high-quality and well-ordered crystals, which is sometimes still cumbersome. The resolution mentioned in the table gives an idea of the level of detail obtained in the structure (the lower the value, the better its quality). NMR spectroscopy determines the structure in solution and is only feasible for small proteins. Using this technique, an "ensemble" of structures is obtained, thus visualizing where the more flexible parts are located in the protein. Finally, cryo-EM (cryo-electron microscopy) is the newest technology. It allows structure determination of very large and complex molecules (even large membrane-integrated complexes) using samples prepared in vitreous ice. In recent years, this technique was much refined and high-resolution structures, comparable to those obtained by X-ray crystallography, may be obtained nowadays. A comparison of the techniques is described by Nwanochie and Uversky (2019). Statistics on the use of these technologies in the field of biomolecular structure determination can be found in: https://www.rcsb.org/stats.

Table 3. List of all pdb files used in the main paper and in the Supplementary Material.

Accession number	Resolution	Method used	Data
6VXX.pdb	2.8 Å	Cryo-EM	[a] SARS-CoV-2 spike ectodomain structure, chains A, B, C (all closed)
6VYB.pdb	3.2 Å	Cryo-EM	[a] SARS-CoV-2 spike ectodomain structure, chains A, B, C (one open: B)
6VSB.pdb	3.46 Å	Cryo-EM	[b] SARS-CoV-2 spike ectodomain structure, chains A, B, C (one open: A)
5XLR.pdb	3.8 Å	Cryo-EM	[c] SARS-CoV spike glycoprotein structure, chains A, B, C (all closed)
5WRG.pdb	4.3 Å	Cryo-EM	[c] SARS-CoV spike glycoprotein structure, chains A, B, C (one open)
5X5C.pdb	4.1 Å	Cryo-EM	[d] MERS-CoV Spike Glycoprotein structure, chains A, B, C (one open: C)
5X5F.pdb	4.2 Å	Cryo-EM	[d] MERS-CoV Spike Glycoprotein structure, chains A, B, C (two open: A, C)
6VW1.pdb	2.68 Å	X-ray	[e] SARS-CoV-2 receptor-binding domain (chains E, F)<br>complexed with its receptor human ACE2 (chains A, B)
4KR0.pdb	2.70 Å	X-ray	[f] Binding between human coronavirus MERS-CoV (chain B)<br>and its receptor CD26 (Dipeptidyl Peptidase 4) (chain A)
1R42.pdb	2.2 Å	X-ray	[g] Human angiotensin-converting enzyme-related carboxypeptidase ACE2 (chain A)
4BXK.pdb	2.2 Å	X-ray	[h] Human angiotensin-converting enzyme ACE, residues 30-657 (chains A, B)
4APH.pdb	1.99 Å	X-ray	[h] Human angiotensin-converting enzyme ACE, residues 642-1230 (chain A)
6LXT.pdb	2.9 Å	X-ray	[i] Structure of post fusion core of 2019-nCoV S2 subunit (chains A, B, C, D, E, F)
6XRA.pdb	3.0 Å	Cryo-EM	[j] SARS-CoV-2 spike protein in post-fusion state, chains A, B, C
6Z2M.pdb	2.71 Å	X-ray	[k] Ternary complex SARS-CoV-2 RBD (chains A,E), with CR3022 Fab<br>fragment of a classical antibody (chains B,H heavy and C,L light chains)<br>and H11-D4 nanobody (chains D,F)
6W41.pdb	3.08 Å	X-ray	[k] Structure of SARS-CoV-2 RBD (chain C)<br>in complex with human antibody CR3022 (chains H, L)

6YZ5.pdb	1.8 Å	X-ray	[l] Structure of SARS-CoV-2 RBD (chain E) in complex with nanobody H11-D4 (chain F)
6XC2.pdb	3.11 Å	X-ray	[m] Structure of SARS-CoV-2 RBD (chains A, Z) in complex with neutralizing antibody CC12.1 (chains H, X and L, Y)
6XC4.pdb	2.34 Å	X-ray	[m] Structure of SARS-CoV-2 RBD (chains A, Z) in complex with neutralizing antibody CC12.3 (chains H, X and L, Y)
7BWJ.pdb	2.85 Å	X-ray	[n] Structure of SARS-CoV-2 RBD (chain E) complexed with a neutralizing antibody (chains H, L)
7BZ5.pdb	1.84 Å	X-ray	[o] Structure of SARS-CoV-2 RBD (chain A) complexed with a neutralizing antibody (chains H, L)
7C01.pdb	2.88 Å	X-ray	[p] Structure of potent human neutralizing Fab (chains H, C and L, D) targeting SARS-CoV-2 RBD (chains A, B)
6WPS.pdb	3.1 Å	Cryo-EM	[q] Structure of the SARS-CoV-2 spike glycoprotein, closed (chains A, B, E) in complex with the S309 neutralizing antibody Fab fragments (chains H, L; C, D; F, G)
7C2L.pdb	3.1 Å	Cryo-EM	[r] Structure of SARS-CoV-2 spike protein (chains A, B, C) in complex with neutralizing antibody 4A8 Fab fragments against NTD (chains H, L; I, M; J, N)
5X29.pdb	–	NMR	[s] Structure of SARS-CoV protein E construct in LMPG micelles (chains A, B, C, D, E); 16 models
6M3M.pdb	2.7 Å	X-ray	[t] Structure of SARS-CoV-2 nucleocapsid protein N: N-terminal RNA binding domain (chains A, B, C, D)
2OG3.pdb	1.85 Å	X-ray	[u] Structure of SARS-CoV nucleocapsid protein N: N-terminal RNA binding domain (chain A)
4UD1.pdb	2.48 Å	X-ray	[v] Structure of MERS-CoV nucleocapsid protein N: N-terminal RNA binding domain (chains A, B, C, D, E)
6WJI.pdb	2.05 Å	X-ray	[w] Structure of SARS-CoV-2 nucleocapsid phosphoprotein N: C-terminal dimerization domain (chains A-B, C-D, E-F)
2CJR.pdb	2.5 Å	X-ray	[x] Structure of SARS-CoV nucleocapsid phosphoprotein N: C-terminal dimerization domain (chains A-B, C-D, E-F, G-H)
6G13.pdb	1.97 Å	X-ray	[y] Structure of MERS-CoV nucleocapsid phosphoprotein N: C-terminal dimerization domain (chains A-C, B-D)
6M0J.pdb	2.45 Å	X-ray	[z] Structure of the SARS-CoV-2 spike receptor-binding domain (chain E) bound to the human ACE2 receptor (chain A)

[a] SARS-CoV-2 S protein's ectodomain (with a C-terminal His-tag and some mutations, e.g., a $G^{682}SAS^{685}$ substitution at the furin cleavage site for stability reasons) was produced in HEK293 (human embryo kidney) cells (Walls et al., 2020).

[b] SARS-CoV-2 S protein's ectodomain (with a C-terminal His-tag and some mutations, e.g., a $G^{682}SAS^{685}$ substitution at the furin cleavage site for stability reasons) was produced in human FreeStyle293F cells (Wrapp et al., 2020b).

[c] SARS-CoV S protein's ectodomain (with a C-terminal Strep-tag) was produced in Sf9 (insect) cells (Gui et al., 2017).

[d] MERS-CoV S S protein's ectodomain (with a C-terminal His-tag) was produced in Sf9 or insect (High Five) cells (Yuan et al., 2017).

[e] all proteins (with a C-terminal His-tag) were expressed in and purified from Sf9 insect cells (Shang et al., 2020b).

[f] both His-tagged CD26 and MERS-CoV RBD proteins were expressed in insect (High Five) cells (Lu et al., 2013).

[g] ACE2 protein was expressed in insect Sf9 cells (Towler et al., 2004).

[h] the N- and C-domains of ACE were expressed and purified from CHO (Chinese-hamster ovary) cells (Douglas et al., 2014; Masuyer et al., 2012).

[i] coding sequences of HR1 (residues 910–988) and HR2 (residues 1162–1206) domains of SARS-CoV-2 S2 subunits were tandemly linked though a 6-residue linker (SGGRGG) and expressed in *E. coli* cells (Xia et al., 2020).

[j] SARS-CoV-2 spike protein was produced in human Expi293F cells and purified; some spike proteins lost their S1 domains, from which S2 domains were isolated (Cai et al., 2020).

[k] RBDs were cloned and expressed (with a C-terminal His-tag) in High Five insect cells; CR3022 Fab fragments were cloned and expressed in human Expi293F cells (Yuan et al., 2020b).

[l] RBDs were cloned and expressed (with a C-terminal His-tag) in HEK293T (human embryonic kidney) cells; nanobody H11-D4 was isolated from a llama VHH library (Huo et al., 2020).

[m] SARS-CoV-2 RBD (with C-terminal His-tag) was purified from insect (High Five) cells; Fab fragments were cloned and expressed in ExpiCHO (Chinese hamster ovary) cells (Yuan et al., 2020a).

[n] SARS-CoV-2 RBD (with C-terminal His-tag) was purified from insect (Sf9) cells; Fab fragments were cloned and expressed in human Expi293F cells (Ju et al., 2020).

[o] SARS-CoV-2 RBD was expressed and purified from *E. coli* cells; Fab fragments were cloned and expressed in HEK293T (human embryonic kidney) cells (Wu et al., 2020).

[p] SARS-CoV-2 RBD (with C-terminal His-tag) was purified from HEK293T (human embryonic kidney) cells; Fab fragments were isolated from PBMCs of patients recovering from infections with SARS-CoV-2 (Shi et al., 2020).

[q] SARS-CoV-2 S glycoprotein ectodomain was produced in HEK293F cells; recombinant antibody S309 was expressed in ExpiCHO cells (Pinto et al., 2020).

[r] SARS-CoV-2 extracellular domain of protein S was expressed using HEK 293F mammalian cells; recombinant mAbs were produced in human Expi293F cells (Chi et al., 2020).
[s] SARS-CoV protein E was expressed in and purified from *E. coli* cells (Surya et al., 2018).
[t] SARS-CoV-2 N-terminal domain of protein N was expressed in and purified from *E. coli* cells (Kang et al., 2020).
[u] SARS-CoV N-terminal domain of protein N was expressed in and purified from bacterial cells (Saikatendu et al., 2007).
[v] MERS-CoV N-terminal domain of protein N was expressed in and purified from *E. coli* cells (Papageorgiou et al., 2016).
[w] (no reference).
[x] SARS-CoV C-terminal domain of protein N was expressed in and purified from *E. coli* cells (Chen et al., 2007).
[y] MERS-CoV C-terminal domain of protein N was expressed in and purified from *E. coli* cells (Nguyen et al., 2018).
[z] SARS-CoV-2 RBD and human ACE2 were expressed in and purified from Sf9 insect cells (Lan et al., 2020).

References

Armenteros, J.J.A., Tsirigos, K.D., Sønderby, C.K., Petersen, T.N., Winther, O., Brunak, S., et al. (2019). SignalP 5.0 improves signal peptide predictions using deep neural networks. Nature Biotechnol., 37, 420-423. https://doi.org/10.1038/s41587-019-0036-z.

Cai, Y., Zhang, J., Xiao, T., Peng, H., Sterling, S.M., Walsh Jr., R.M., et al. (2020). Distinct conformational states of SARS-CoV-2 spike protein. Science 369, 1586-1592. https://doi.org/10.1126/science.abd4251.

Chen, C-Y., Chang, C-k., Chang, Y-W., Sue, S-C., Bai, H-I., Riang, L., et al. (2007). Structure of the SARS coronavirus nucleocapsid protein RNA-binding dimerization domain suggests a mechanism for helical packaging of viral RNA. J. Mol. Biol. 368, 1075-1086. https://doi.org/10.1016/j.jmb.2007.02.069.

Chi, X., Yan, R., Zhang, J., Zhang, G., Zhang, Y., Hao, M., et al. (2020). A neutralizing human antibody binds to the N-terminal domain of the Spike protein of SARS-CoV-2. Science 369, 650-655. https://doi.org/10.1126/science.abc6952.

Douglas, R.G., Sharma, R.K., Masuyer, G., Lubbe, L., Zamora, I., Acharya, K.R., et al. (2014). Fragment-based design for the development of N-domain-selective angiotensin-1-converting enzyme inhibitors. Clin. Sci. 126, 305-313. https://doi.org/10.1042/cs20130403.

Drozdetskiy, A., Cole, C., Procter, J., and Barton, G.J. (2015). JPred4: a protein secondary structure prediction server. Nucleic Acids Res. 43(W1), W389-394. https://doi.org/10.1093/nar/gkv332.

Guex, N., and Peitsch, M.C. (1997). SWISS-MODEL and the Swiss-PdbViewer: an environment for comparative protein modeling. Electrophoresis 18, 2714-2723. https://doi.org/10.1002/elps.1150181505.

Guex, N., Peitsch, M.C., and Schwede, T. (2009). Automated comparative protein structure modeling with SWISS-MODEL and Swiss-PdbViewer: A historical perspective. Electrophoresis 30, S162-S173. https://doi.org/10.1002/elps.200900140.

Gui, M., Song, W., Zhou, H., Xu, J., Chen, S., Xiang, Y. and Wang X. (2017). Cryo-electron microscopy structures of the SARS-CoV spike glycoprotein reveal a prerequisite conformational state for receptor binding. Cell Res. 27, 119-129. https://doi.org/10.1038/cr.2016.152.

Gupta, R., and Brunak, S. (2002). Prediction of glycosylation across the human proteome and the correlation to protein function. Pacific Symp. Biocomput. 7, 310-322. PMID: 11928486.

Hirokawa, T., Boon-Chieng, S., and Mitaku, S. (1998). SOSUI: classification and secondary structure prediction system for membrane proteins. Bioinformatics 14, 378-379. https://doi.org/10.1093/bioinformatics/14.4.378.

Huo, J., Le Bas, A., Ruza, R.R., Duyvesteyn, H.M.E., Mikolajek, H., Malinauskas, T., et al. (2020). Neutralizing nanobodies bind SARS-CoV-2 spike RBD and block interaction with ACE2. Nature Struct. Mol. Biol. https://doi.org/10.1038/s41594-020-0469-6.

Ju, B., Zhang, Q., Ge, J., Wang, R., Sun, J., Ge, X., et al. (2020). Human neutralizing antibodies elicited by SARS-CoV-2 infection. Nature 584, 115-119. https://doi.org/10.1038/s41586-020-2380-z.

Junier, T., and Pagni, M. (2000). Dotlet: diagonal plots in e Web browser. Bioinformatics 16, 178-179. https://doi.org/10.1093/bioinformatics/16.2.178.

Käll, L., Krogh, A., and Sonnhammer, E.L. (2004). A combined transmembrane topology and signal peptide prediction method. J. Mol. Biol. 338, 1027-1036. https://doi.org/10.1016/j.jmb.2004.03.016.

Kang, S., Yang, M., Hong, Z., Zhang, L., Huang, Z., Chen, X., et al. (2020). Crystal structure of SARS-CoV-2 nucleocapsid protein RNA binding domain reveals potential unique drug targeting sites. Acta Pharm. Sinica B. 10, 1228e1238. https://doi.org/10.1016/j.apsb.2020.04.009.

Krogh, A., Larsson, B., von Heijne, G., Sonnhammer, E.L. (2001). Predicting transmembrane protein topology with a hidden Markov model: application to complete genomes. J. Mol. Biol. 305, 567-580. https://doi.org/10.1006/jmbi.2000.4315.

Lan, J., Ge, J., Yu, J., Shan, S., Zhou, H., Fan, S., et al. (2020). Structure of the SARS-CoV-2 spike receptor-binding domain bound to the ACE2 receptor. Nature 581, 215-220. https://doi.org/10.1038/s41586-020-2180-5.

Lu, G., Hu, Y., Wang, Q., Qi, J., Gao, F., Li, Y., et al. (2013). Molecular basis of binding between novel human coronavirus MERS-CoV and its receptor CD26. Nature 500, 227-231. https://doi.org/10.1038/nature12328.

Makwana, K.M., and Mahalakshmi, R. (2015). Implications of aromatic-aromatic interactions: from protein structures to peptide models. Prot. Sci. 24, 1920-1933. https://doi.org/10.1002/pro.2814.

Masuyer, G., Schwager, S.L., Sturrock, E.D., Isaac, R.E., and Acharya, K.R. (2012). Molecular recognition and regulation of human angiotensin-I converting enzyme (ACE) activity by natural inhibitory peptides. Sci. Rep. 2, 717. https://doi.org/10.1038/srep00717.

Mészáros, B., Erdős, G., and Dosztányi, Z. (2018). IUPred2A: context-dependent prediction of protein disorder as a function of redox state and protein binding. Nucl. Acids Res. 46(W1), W329-W337. https://doi.org/10.1093/nar/gky384.

Naveca, F., Nascimento, V., Souza, V., Corado, A., Nascimento, F., Silva, G., et al. (2021). Phylogenetic relationship of SARS-CoV-2 sequences from Amazonas with emerging Brazilian variants harboring mutations E484K and N501Y in the Spike protein. https://virological.org/t/phylogenetic-relationship-of-SARS-cov-2-sequences-from-amazonas-with-emerging-brazilian-variants-harboring-mutations-e484k-and-n501y-in-the-spike-protein/585/1.

Nguyen, T.H.V., Lichière, J., Canard, B., Papageorgiou, N., Attoumani, S., Ferrona, F., and Coutard, B. (2018). Structure and oligomerization state of the C-terminal region of the Middle East respiratory syndrome coronavirus nucleoprotein. Acta Cryst. D75, 8-15. https://doi.org/10.1107/S2059798318014948.

Nielsen, H., Engelbrecht, J., Brunak, S., and von Heijne, G. (1997). Identification of prokaryotic and eukaryotic signal peptides and prediction of their cleavage sites. Protein Eng. 10, 1-6. https://doi.org/10.1093/protein/10.1.1.

Nwanochie, E., and Uversky, V.N. (2019). Structure determination by single-particle cryo-electron microscopy: only the sky (and intrinsic disorder) is the limit. Int. J. Mol. Sci. 20, 4186. https://doi.org/10.3390/ijms20174186.

Papageorgiou, N., Lichière, J., Baklouti, A., Ferron, F., Sévajol, M., Canard, B., and Coutard, B. (2016). Structural characterization of the N-terminal part of the MERS-CoV nucleocapsid by X-ray diffraction and small-angle X-ray scattering. Acta Cryst. D72, 192-202. http://dx.doi.org/10.1107/S2059798315024328.

Pinto, D., Park, Y.J., Beltramello, M., Walls, A.C., Tortorici, M.A., Bianchi, S., et al. (2020). Cross-neutralization of SARS-CoV-2 by a human monoclonal SARS-CoV antibody. Nature 583, 290-295. https://doi.org/10.1038/s41586-020-2349-y.

Sabino, E.C., Buss, L.F., Carvalho, M.P.S., Prete Jr, C.A., Crispim, M.A.E., Fraiji, N.A., et al. (2021). Resurgence of COVID-19 in Manaus, Brazil, despite high seroprevalence. The Lancet. https://doi.org/10.1016/S0140-6736(21)00183-5.

Saikatendu, K.S., Joseph, J.S., Subramanian, V., Neuman, B.W., Buchmeier, M.J., Stevens, R.C., and Kuhn, P. (2007). Ribonucleocapsid formation of Severe Acute Respiratory Syndrome Coronavirus through molecular action of the N-terminal domain of N protein. J. Virol. 81, 3913-3921. https://doi.org/10.1128/JVI.02236-06.

Shang, J., Ye, G., Shi, K., Wan, Y., Luo, C., Aihara, H., et al. (2020b). Structural basis of receptor recognition by SARS-CoV-2. Nature 581, 221-224. https://doi.org/10.1038/s41586-020-2179-y.

Shi, R., Shan, C., Duan, X., Chen, Z., Liu, P., Song, J., et al. (2020). A human neutralizing antibody targets the receptor-binding site of SARS-CoV-2. Nature 584, 120-124. https://doi.org/10.1038/s41586-020-2381-y.

Sievers, F., Wilm, A., Dineen, D., Gibson, T.J., Karplus, K., Li, W., et al. (2011). Fast, scalable generation of high-quality protein multiple sequence alignments using Clustal Omega. Mol. Syst. Biol. 7, 539. https://doi.org/10.1038/msb.2011.75.

Surya, W., Li, Y., and Torres, J. (2018). Structural model of the SARS coronavirus E channel in LMPG micelles. Biochim. Biophys. Acta (Biomembranes) 1860, 1309-1317. https://doi.org/10.1016/j.bbamem.2018.02.017.

Tegally, H., Wilkinson, E., Giovanetti, M., Iranzadeh, A., Fonseca, V., Giandhari, J., et al. (2020). Emergence and rapid spread of a new severe acute respiratory syndrome-related coronavirus 2 (SARS-CoV-2) lineage with multiple spike mutations in South Africa. medRxiv preprint. https://doi.org/10.1101/2020.12.21.20248640.

Towler, P., Staker, B., Prasad, S.G., Menon, S., Tang, J., Parsons, T., et al. (2004). ACE2 X-ray structures reveal a large hinge-bending motion important for inhibitor binding and catalysis. J. Biol. Chem. 279, 17996-8007. https://doi.org/10.1074/jbc.M311191200.

Tusnády, G.E., and Simon, I. (1998). Principles governing amino acid composition of integral membrane proteins: application to topology prediction. J. Mol. Biol. 283, 489-506. http://doi.org/10.1006/jmbi.1998.2107.

Walls, A.C., Park, Y-J., Tortorici, M.A., Wall, A., McGuire, A.T., and Veesler, D. (2020). Structure, function, and antigenicity of the SARSCoV-2 spike glycoprotein. Cell 180, https://doi.org/10.1016/j.cell.2020.02.058.

Wan, Y., Jian Shang, J., Graham, R., Baric, R.S., and Li, F. (2020). Receptor recognition by the novel coronavirus from Wuhan: an analysis based on decade-long structural studies of SARS coronavirus. J. Virology 94 (7), e00127-20. https://doi.org/10.1128/JVI.00127-20.

Wrapp, D., Wang, N., Corbett, K.S., Goldsmith, J.A., Hsieh, C-L., Abiona, O., et al. (2020b). Cryo-EM structure of the 2019-nCoV spike in the prefusion conformation. Science 367, 1260-1263. http://doi.org/10.1126/science.abb2507.

Wu, Y., Wang, F., Shen, C., Peng, W., Li, D., Zhao, C., et al. (2020). A noncompeting pair of human neutralizing antibodies block COVID-19 virus binding to its receptor ACE2. Science 368, 1274-1278. http://doi.org/10.1126/science.abc2241.

Xia, S., Liu, M., Wang, C., Xu, W., Lan, Q., Feng, S., et al. (2020). Inhibition of SARS-CoV-2 (previously 2019-nCoV) infection by a highly potent pan-coronavirus fusion inhibitor targeting its spike protein that harbors a high capacity to mediate membrane fusion. Cell Res. 30, 343-355. https://doi.org/10.1038/s41422-020-0305-x.

Yuan, Y., Cao, D., Zhang, Y., Ma, J., Qi, J., Wang, Q., et al. (2017). Cryo-EM structures of MERS-CoV and SARS-CoV spike glycoproteins reveal the dynamic receptor binding domains. Nat. Commun. 8, 15092. https://doi.org/10.1038/ncomms15092.

Yuan, M., Liu, H., Wu, N.C., Lee, C-C.D., Zhu, X., Zhao, F., et al. (2020a). Structural basis of a shared antibody response to SARS-CoV-2. Science 369, 1119-1123. https://doi.org/10.1126/science.abd2321.

Yuan, M., Wu, N.C., Zhu, X., Lee, C-C.D., So, R.T.Y., Lv, H., et al. (2020b). A highly conserved cryptic epitope in the receptor binding domains of SARS-CoV-2 and SARS-CoV. Science 368, 630-633. https://doi.org/10.1126/science.abb7269.
